# Supplementary material for: Uncovering combination therapies for immune-mediated inflammatory diseases through systems biology analysis on longitudinal patient data
Source: Cell Rep Med. 2026 Sep 11;7(10):103026. doi: 10.1016/j.xcrm.2026.103026 (PMC13710146; doi:10.1016/j.xcrm.2026.103026)
Supplement: Supplementary file 1 [file mmc1.pdf]

Cell Reports Medicine, Volume *7*

Supplemental information

Uncovering combination therapies for immune-mediated inflammatory diseases through systems biology analysis on longitudinal patient data

Sergio H. Martínez-Mateu, Yolanda Guillén, Edgar Angelats, María A. López-Lasanta, Stefania Madonna, Laura Jiménez-Gracia, Ivan Rodriguez-Nunez, Damiana Álvarez-Errico, Adrià Aterido, Raül Tortosa, Max Ruiz, Pau Serra, Juan D. Cañete, Jose M. Carrascosa, Eugeni Domènech, Javier P. Gisbert, Jesús Tornero, IMID-Consortium, Britta Siegmund, Giampiero Girolomoni, Ernest H.S. Choy, Richard M. Myers, Holger Heyn, Pere Santamaria, Sara Marsal, and Antonio Julià

SUPPLEMENTAL FIGURES

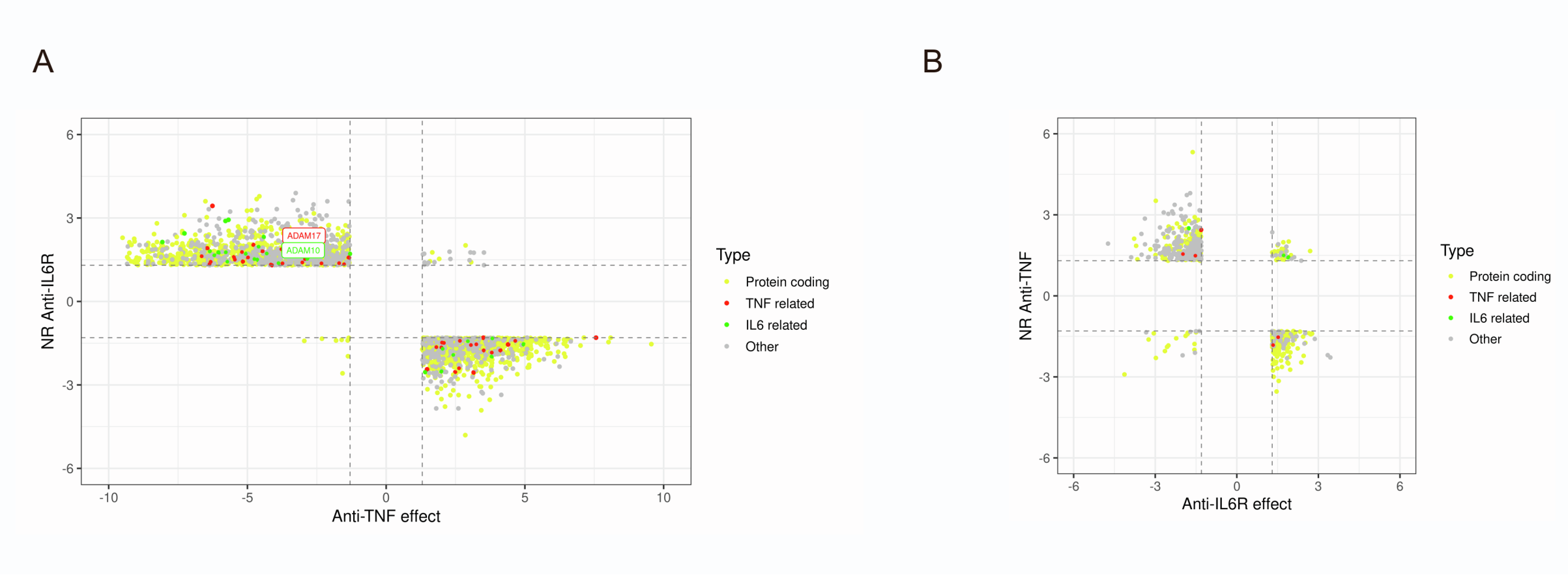

Figure S1. MNRS analysis shows antiIL6R-antiTNF complementarity in RA patients. Plots comparing the non-response signature of one drug (y-axis) with the longitudinal effect of the complementary drug (x-axis). Gene values are shown as log10(P-value) of differential gene expression with the sign of the fold change. (A) Anti-IL6R non-response signature against anti-TNF longitudinal effect. (B) Anti-TNF non-response signature against anti-IL6R longitudinal effect. Colours indicate if the gene is from TNF signalling pathway (red), from IL-6 signalling pathway (green) or, otherwise, if it’s a protein-coding gene (yellow) or non-coding gene (grey). Related to Figure 2.

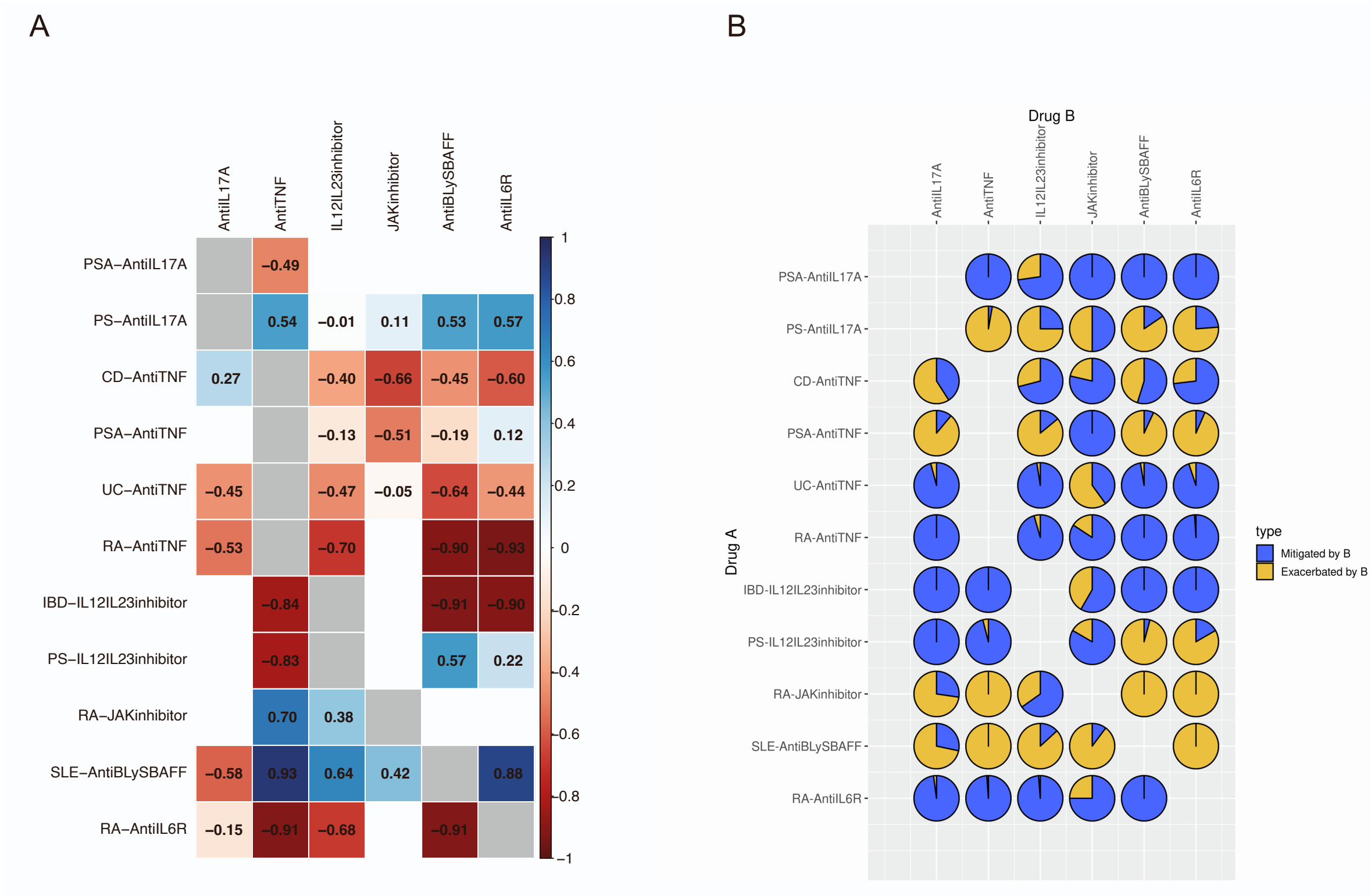

Figure S2. Mitigation of non-response (MNRS) analysis at the pathway level. (A) Pathway-level correlation coefficients between non-response signatures (rows) and drug effect signatures (columns). Pearson correlations were computed using only pathways significant at FDR<0.05 for both signatures. Correlations were not computed if there were less than 20 significant pathways (blank squares). (B) Non-response signature reversal percentages. Pathways associated to non-response (FDR<0.05) in the drug-disease cohort (rows) are categorized based on the longitudinal effect of drug B (columns). Antagonized by B: pathways that are reversed by drug B effect. Exacerbated by B: pathways that are exacerbated by drug B effect. Related to Figure 2.

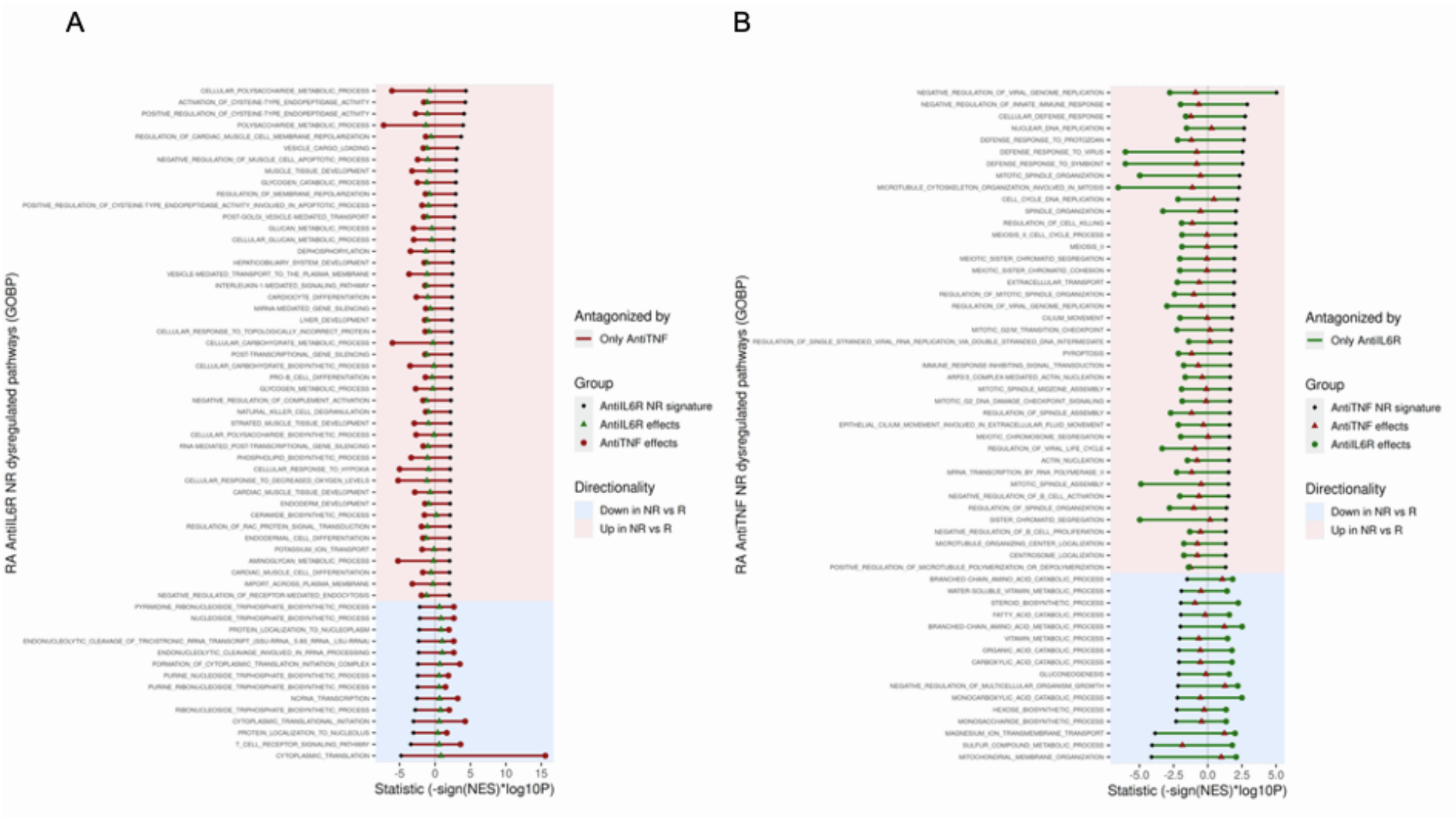

Figure S3. Pathway level characterization of the complementarity between anti-TNF and anti-IL6R in RA. Gene set enrichment analysis (GSEA) was performed using differential gene expression signatures as input. The enrichment statistic is defined shown as log10(P-value) of the enrichment test with the sign of the NES. (A) Pathways significantly associated to anti-IL6R non-response ($p<0.01$) that are significantly modulated by anti-TNF ($p<0.05$) but not by anti-IL6R ($p>0.05$). (B) Pathways significantly associated with anti-TNF non-response ($p<0.05$) that are significantly modulated by anti-IL6R ($p<0.05$) but not by anti-TNF ($p>0.05$). Black diamonds: pathways associated to non-response to therapies at baseline. Green dots/triangles: pathway association to anti-IL6R longitudinal effect. Red dots/triangles: pathway association to ant-TNF longitudinal effect. Related to Figure 2.

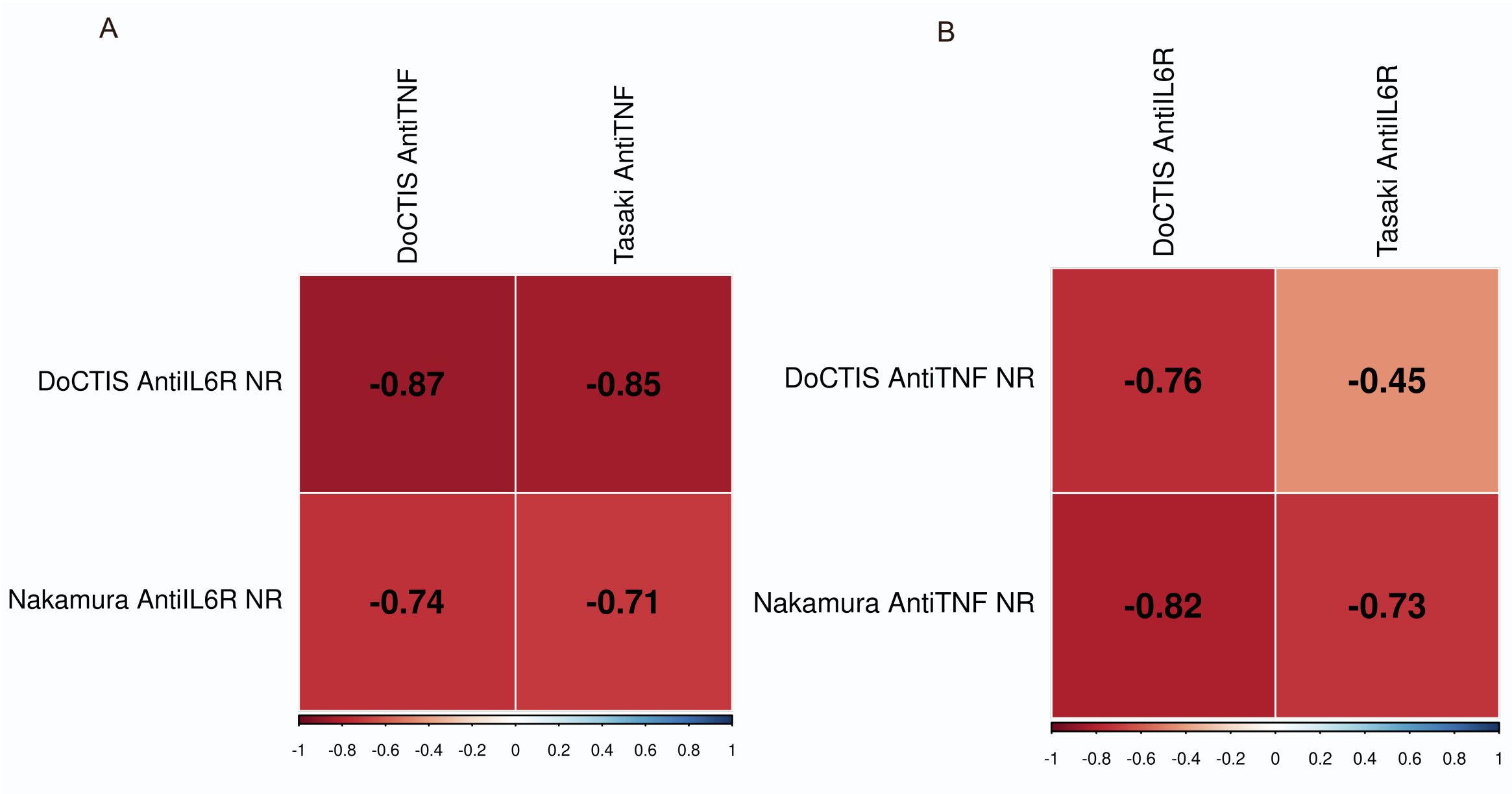

Figure S4. Replication of MNRS complementarity between anti-TNF and anti-IL6R combination in RA. Correlation coefficients between non-response signatures (rows) and drug effect signatures (columns). (A) Non-response signatures to anti-IL6R (rows) from Nakamura (GSE78068) and our study (DoCTIS) against the anti-TNF effect signatures (columns) from Tasaki (GSE93272), DoCTIS RA patients and DoCTIS multi-cohort analysis (global). (B) Non-response signatures (rows) to anti-TNF therapy from Nakamura (GSE78068) and DoCTIS against anti-IL6R treatment effect signatures (columns) from Tasaki (GSE93272) and DoCTIS RA patients. Related to Figure 2.

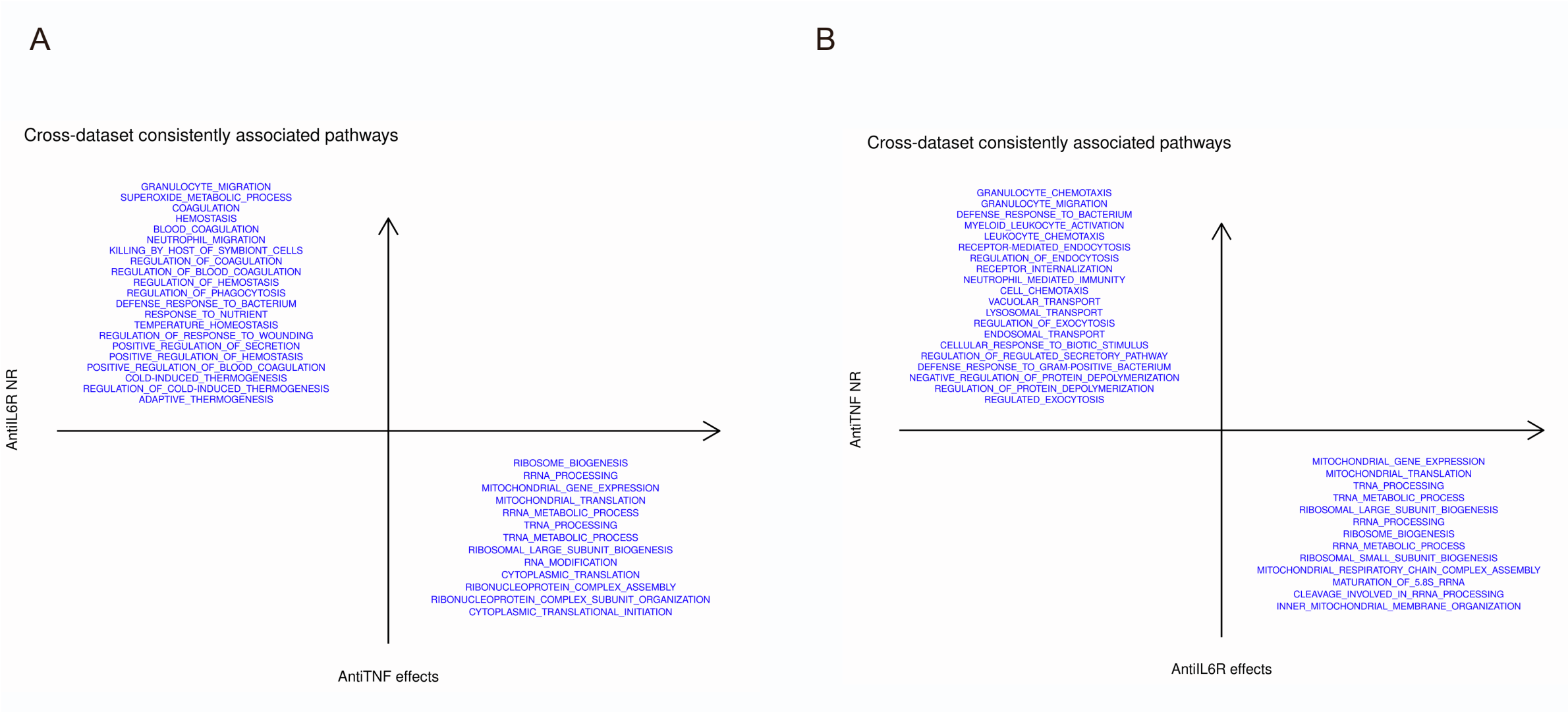

Figure S5. Pathway level analysis of the MNRS validation. Pathways consistently associated across datasets are displayed in four quadrants, based on the direction of their association. (A) Pathways that are consistently associated with anti-IL6R non-response in RA across Nakamura (GSE78068) and DoCTIS, and modulated by anti-TNF therapy in DoCTIS (global) and Tasaki (GSE93272). (B) Anti-TNF non-response signatures in RA from Nakamura (GSE78068) and DoCTIS compared to anti-IL6R treatment effects in DoCTIS RA patients and Tasaki (GSE93272). Related to Figure 2.

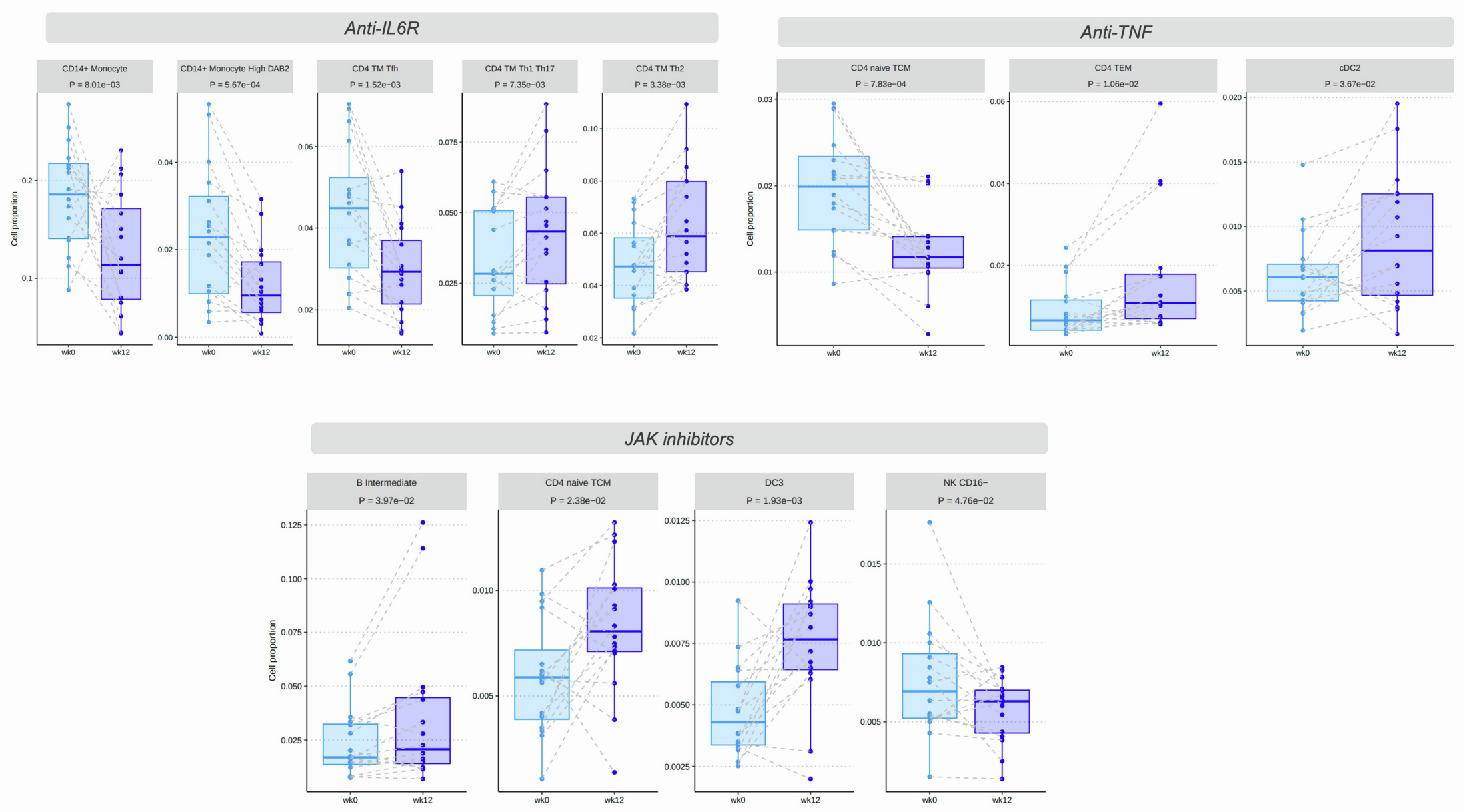

Figure S6. Proportion of cell types before and after therapy. Per each cohort, only cell types showing significant changes before and after therapy (P-value < 0.05) are represented. Related to Figure 3

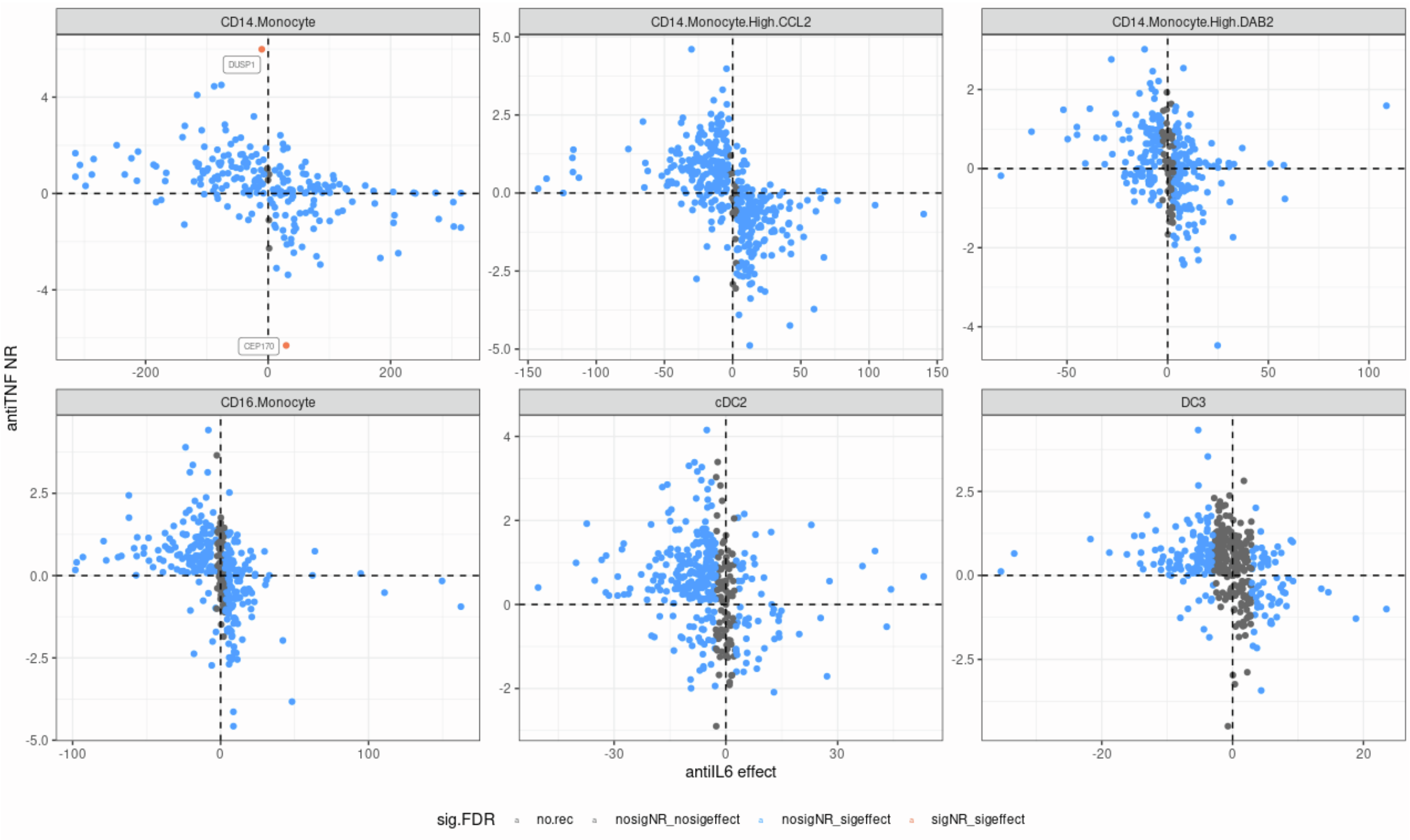

Figure S7. MNRS single cell analysis: anti-TNF non-response vs anti-IL6R longitudinal effect. X axis: anti-IL6R effect signature. Y axis: anti-TNF non-response signature. Values are shown as log10(P-value) of differential gene expression with the sign of the fold change. Only cell types in which the MNRS is significant are shown. Differentially expressed genes ($P\text{-value}_{adjusted} < 0.05$) are labeled. Related to Figure 3.

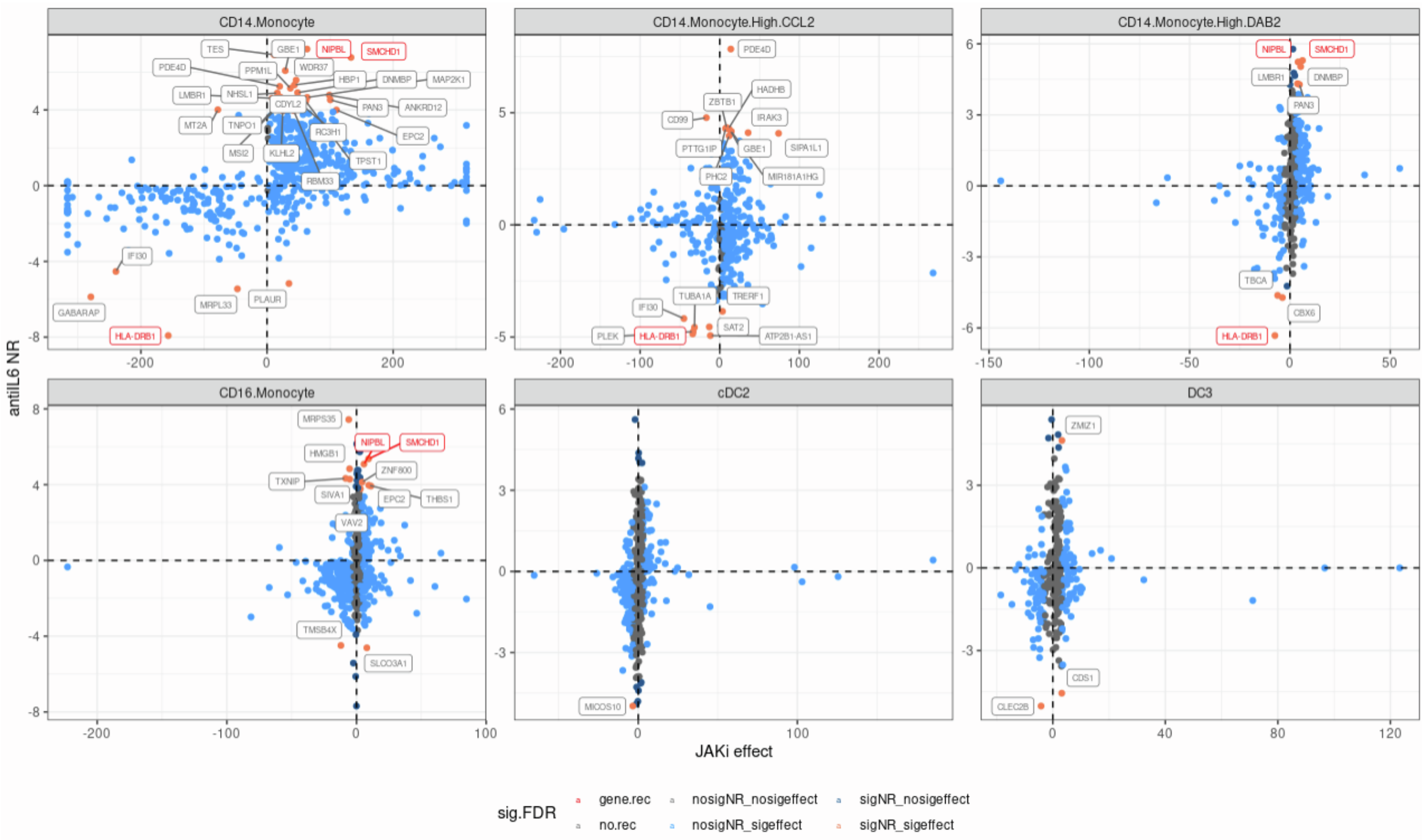

Figure S8. MNRS single cell analysis: anti-IL6R NR vs JAKi effect. X axis: JAKi effect signature. Y axis: anti-IL6R NR signature. Values are shown as log10(P-value) of differential gene expression with the sign of the fold change. Only cell types in which Fisher's exact test is significant are shown. Significantly differentially expressed genes (DEGs) (P-value adj < 0.05) are labeled. Genes differentially expressed in the maximum number of cell types are highlighted in red. Related to Figure 3.

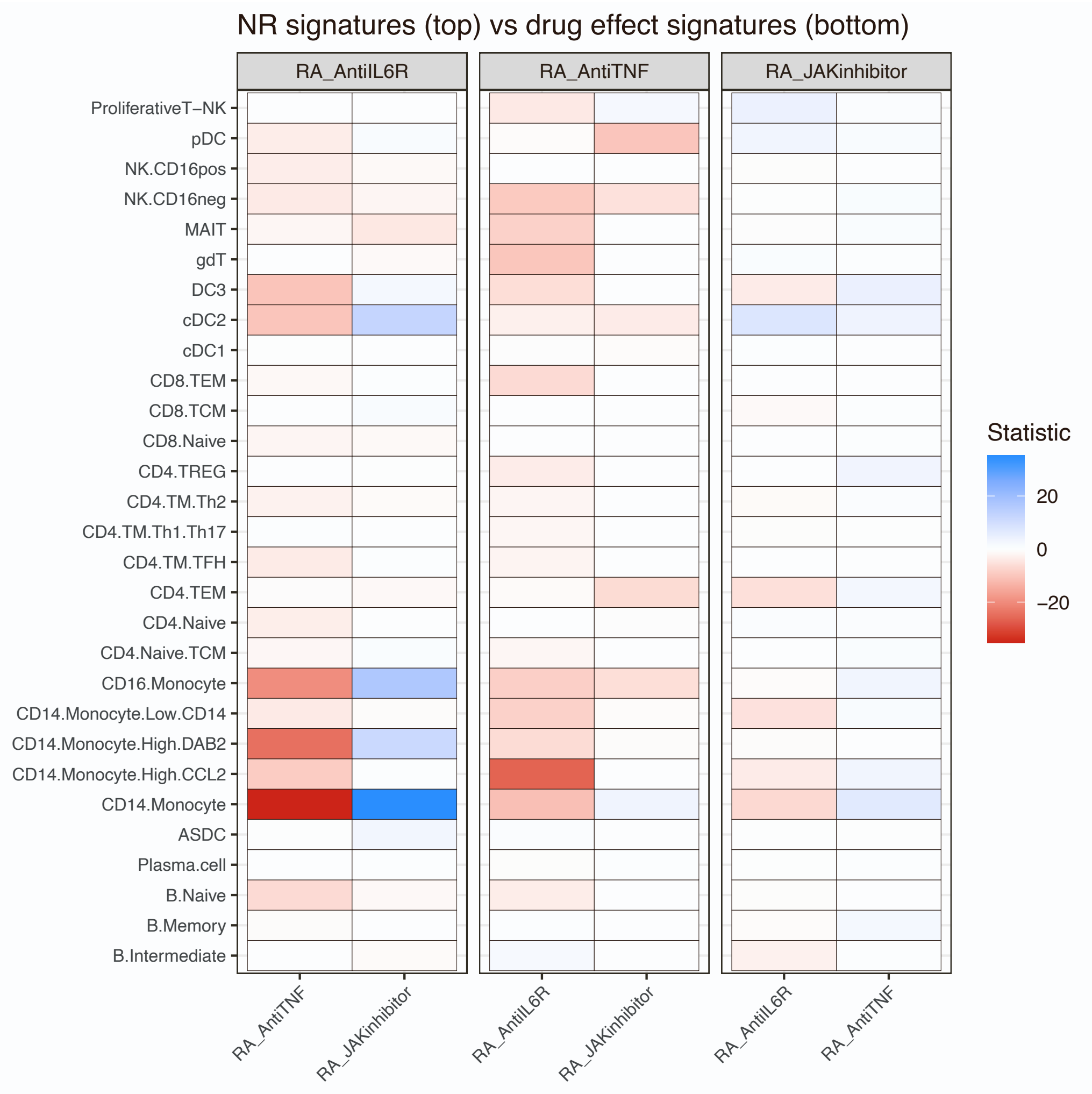

Figure S9. MNRS analysis per cell type on scRNAseq data from RA patients. Significant genes (P<0.05) were classified into four categories based on the directionality of the non-response and longitudinal effect association and the Fisher's test was performed to evaluate enrichment in the antagonization categories. The statistic is the logarithm of the P-value of the Fisher's exact test with sign given by the sign of the logarithm of the odds ratio. Non-response signatures are indicated on the top; effect signatures are indicated on the bottom. Related to Figure 3.

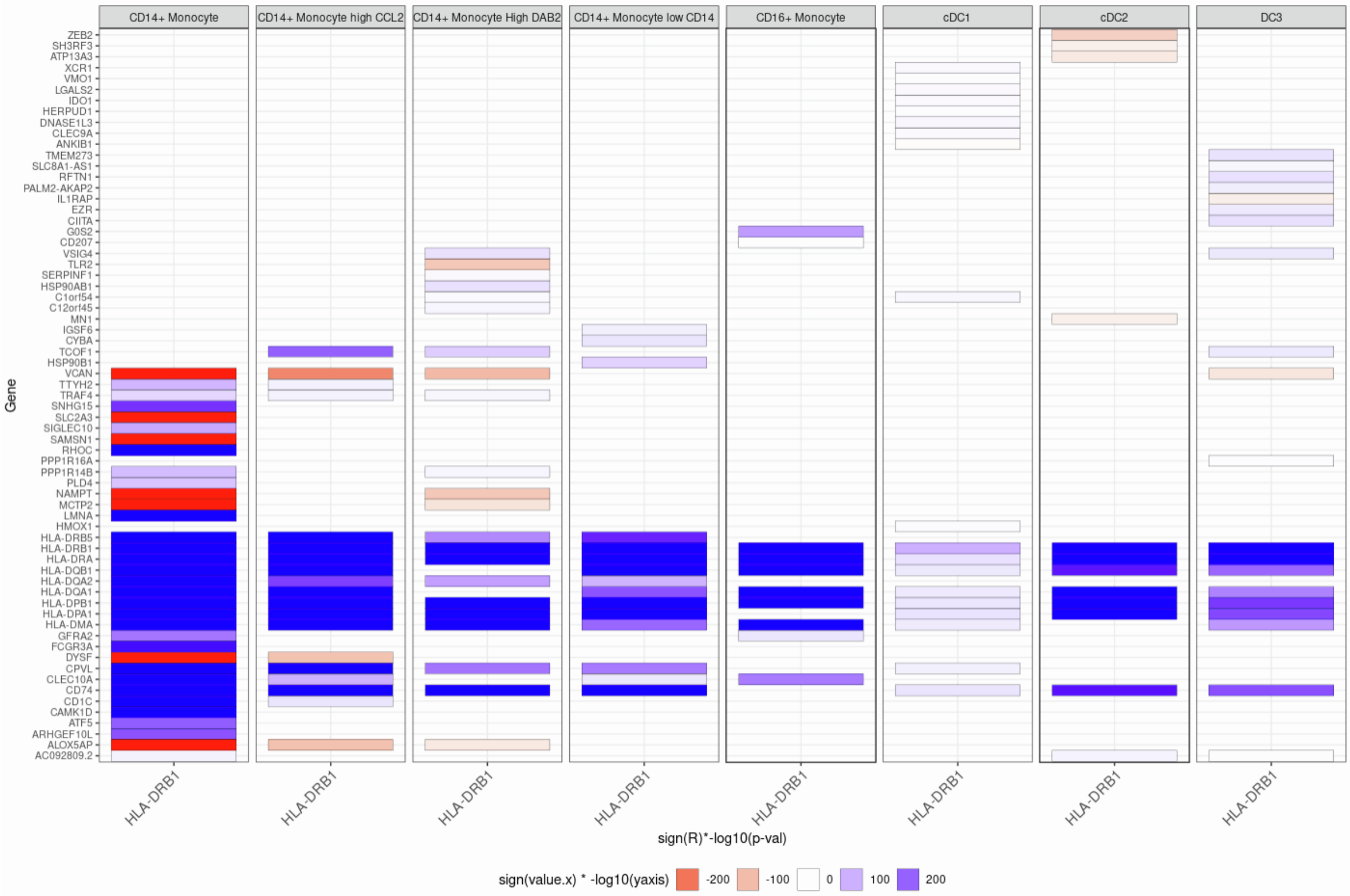

Figure S10. HLA-DRB1 co-expressed genes in myeloid cell subtypes. Gene modules were inferred using CSCORE. All the genes from the HLA-DRB1 module types are included. Significant correlations with HLA-DRB1 (P-value < 0.05, CSCORE estimate > 0.2 (blue) or <-0.2 (red) expression within each cell are shown. Related to Figure 4.

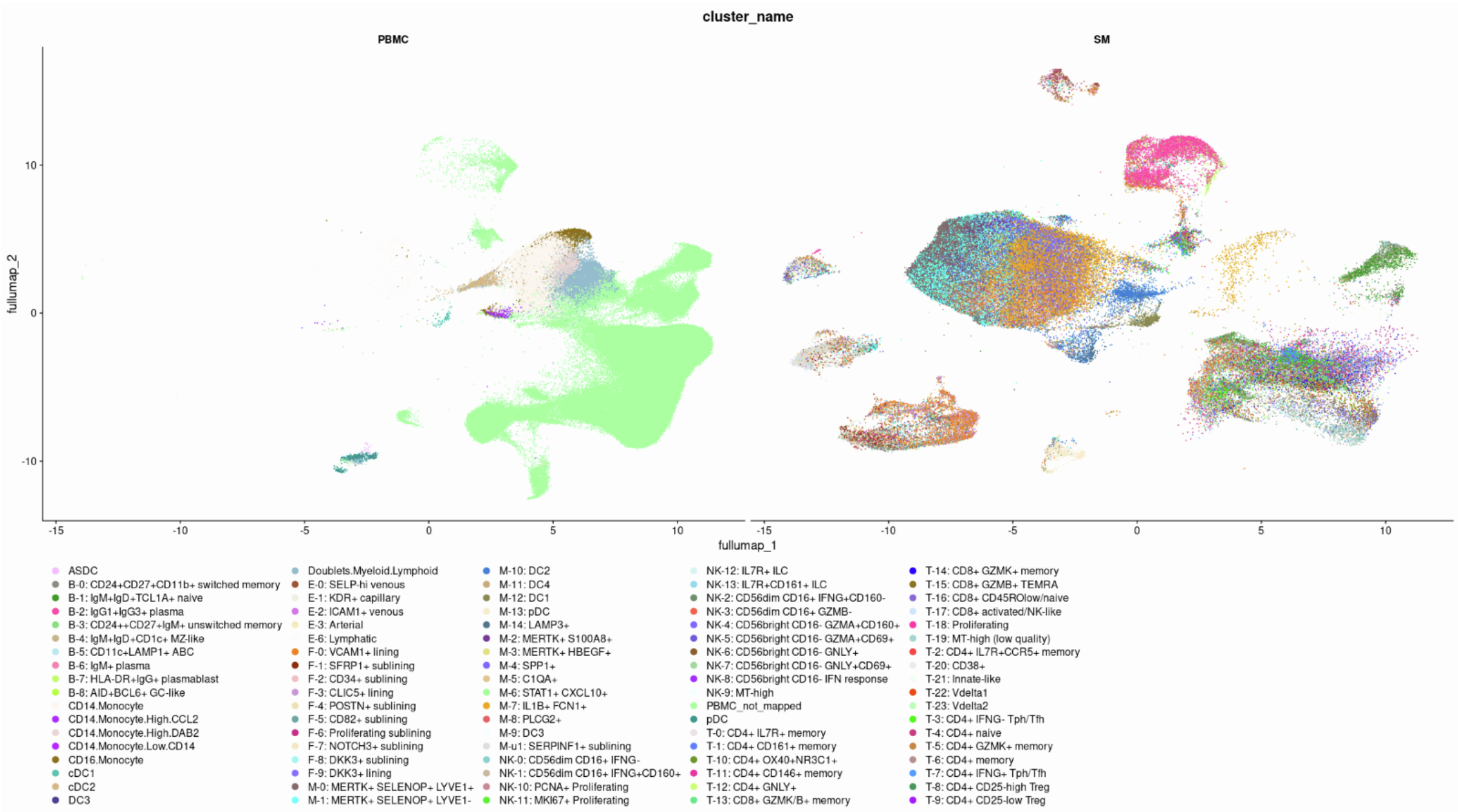

Figure S11. UMAP of scRNA-Seq data of paired PBMCs and synovial membrane samples. PBMCs are represented on the left panel, and synovial membrane cells on the right panel. Myeloid cells from PBMCs were annotated using our PBMC dataset. SM cells were mapped against the Accelerating Medicines Partnership atlas of the RA synovium [23]. Related to Figure 4.

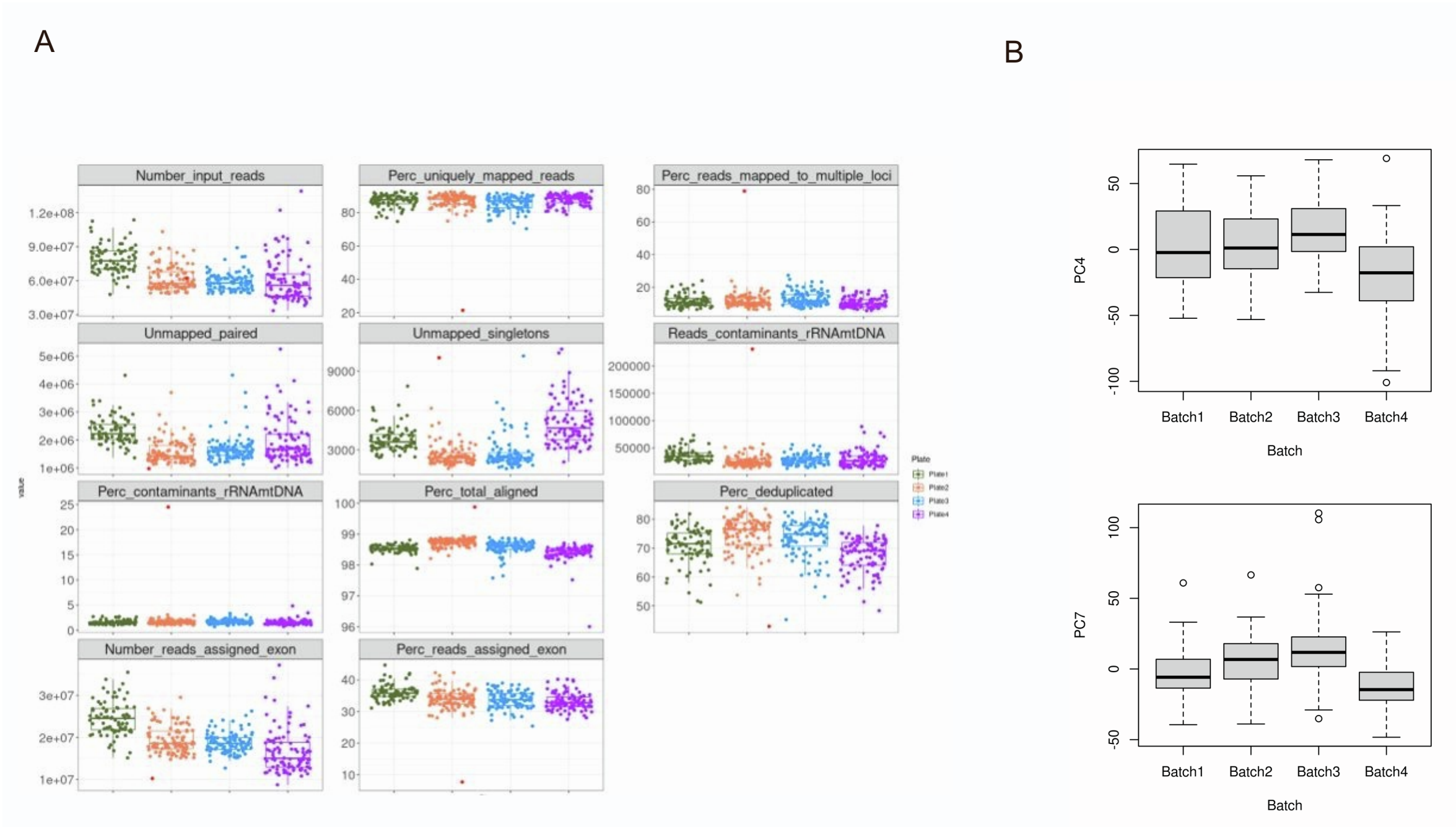

Figure S12. Bulk RNAseq quality control. (A) Association between bulk RNA-Seq parameters and sequencing batch (plate). *Number_input_reads*: Number of input filtered/trimmed reads. *Perc_uniquely_mapped_reads*: Percentage of reads mapped to unique regions of human genome. *Perc_reads_mapped_to_multiple_loci*: Percentage of reads mapping to multiple loci. *Unmapped_paired*: Paired reads that did not map to the human genome. *Unmapped_singletons*: Singleton sequences that did not map to the human genome. *Reads_contaminants_rRNAmtDNA*: Unmapped reads corresponding to rRNA or mtDNA. *Perc_contaminants_rRNAmtDNA*: Percentage of unmapped reads corresponding to rRNA or mtDNA. *Perc_total_aligned*: Sum of % uniquely and % multiple loci mapping reads. *Perc_deduplicated*: Percentage of reads deduplicated. *Number_reads_assigned_exon*: Number of reads assigned to annotated exons (featureCounts). *Perc_reads_assigned_exon*: Percentage of aligned reads mapped to annotated exons (featureCounts). One sample showed a high fraction of rRNA and mtDNA contamination as well as a low fraction of reads mapping to unique regions (red dot). This sample was successfully resequenced. (B) Principal components 4 (4% of explained variance) and 7 (2% of explained variance) of the global bulk gene expression matrix showed a significant association with the sequencing batch and were included as covariates in the differential expression analysis. Related to STAR Methods.

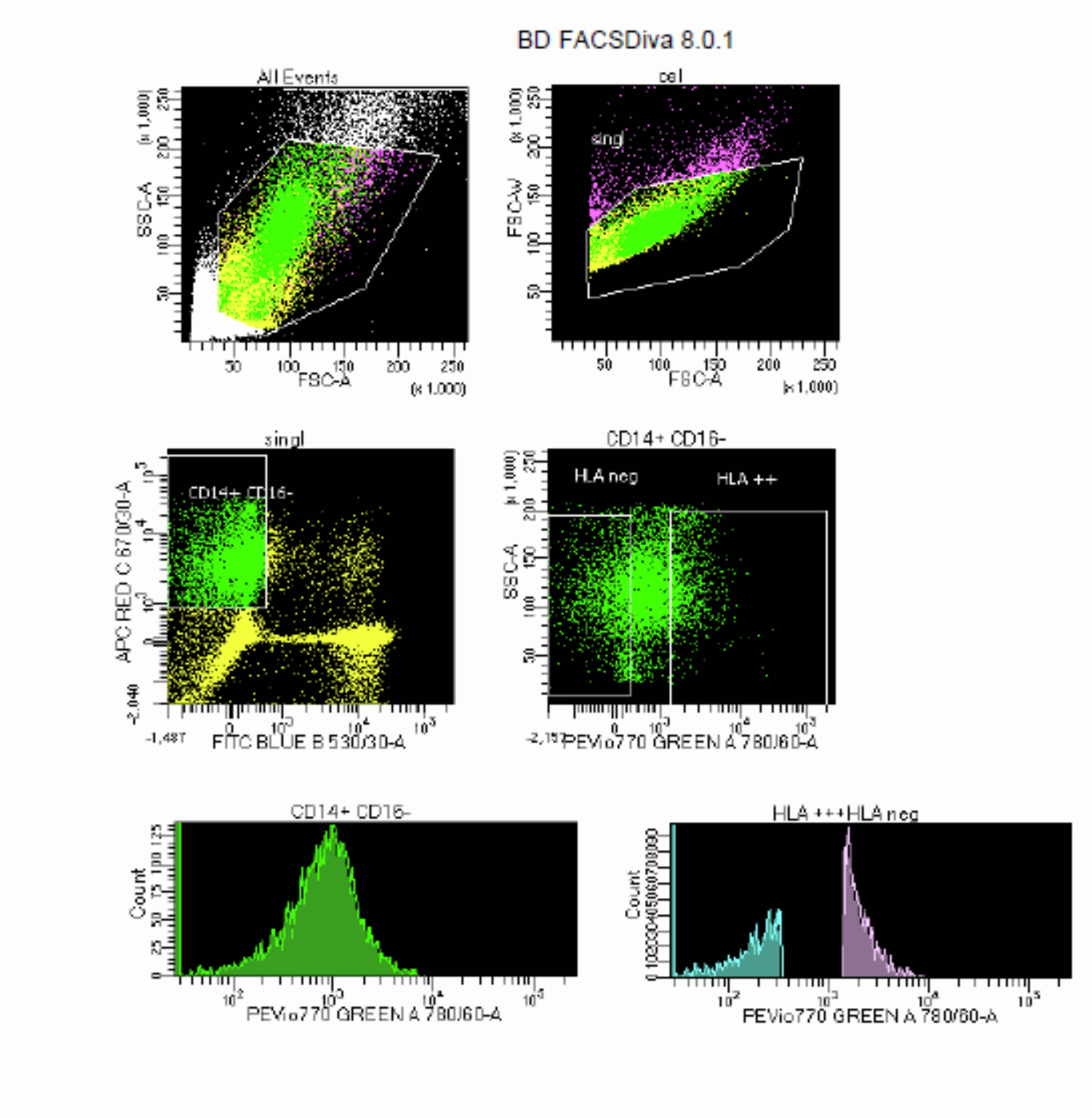

Figure S13. Monocyte-enriched cell suspensions obtained from fresh PBMCs were stained with anti-CD14-APC, anti-CD16-FITC and HLA-DRB1-PE-Vio. HLADRB1^{Hi} and HLADRB1-negative CD14+/CD16- were separated by cell sorting. Gates for separation according to HLADRB1 expression level are shown in the bottom right histogram; HLA-DRB1 negative and HLA++ (i.e. HLA-DRB1^{Hi}), colored as blue and violet, respectively. Related to STAR Methods.

SUPPLEMENTAL TABLES

Table S1. Baseline characteristics of the responders and non-responders from each disease-drug cohort. Related to Figure 1.

Table S1.A: PsA Anti-IL7A

	PSA Anti IL17A		
**Variable**	**R** N = 8	**NR** N = 8	**p-value**[1]
**Sex Female, n (%)**	6 (75%)	7 (88%)	>0.99
**Age, Mean (SD)**	49 (6)	55 (11)	0.43
**BMI, Mean (SD)**	24.0 (4.8)	27.7 (5.6)	0.16
**Cigarette Frequency, n (%)**			>0.99
Daily	2 (25%)	1 (13%)	
Occasionally	0 (0%)	0 (0%)	
Never	6 (75%)	7 (88%)	
**Disease Duration Years, Mean (SD)**	9 (8)	18 (11)	0.083
**Days To Follow-up, Mean (SD)**	94 (14)	86 (6)	0.063
**Concomitant GCs, n (%)**	4 (50%)	6 (75%)	0.61
**Baseline Activity, Median (Q1, Q3)**	4.99 (4.89, 5.64)	4.66 (4.11, 5.11)	0.16

[1]Fisher's exact test; Wilcoxon rank sum test; Wilcoxon rank sum exact test

Table S1.B: SLE Anti-BlyS/BAFF

	SLE Anti BLyS/BAFF		
**Variable**	**R** N = 8	**NR** N = 8	**p-value**[1]
**Sex Female, n (%)**	8 (100%)	8 (100%)	
**Age, Mean (SD)**	35 (13)	44 (13)	0.25
**BMI, Mean (SD)**	27.0 (6.0)	23.3 (3.9)	0.25
**Cigarette Frequency, n (%)**			0.47
Daily	2 (25%)	0 (0%)	
Occasionally	0 (0%)	0 (0%)	
Never	6 (75%)	8 (100%)	
**Disease Duration Years, Mean (SD)**	9 (9)	11 (7)	0.46
**Days To Follow-up, Mean (SD)**	84 (12)	104 (27)	0.14
**Concomitant GCs, n (%)**	6 (75%)	7 (88%)	>0.99
**Baseline Activity, Median (Q1, Q3)**	9.50 (8.00, 10.50)	7.00 (4.50, 8.50)	0.090

[1]NA; Wilcoxon rank sum test; Fisher's exact test

Table S1.C: RA Anti-IL6R

	RA Anti IL6R		
**Variable**	**R** N = 8	**NR** N = 8	**p-value**[1]
**Sex Female, n (%)**	8 (100%)	6 (75%)	0.47
**Age, Mean (SD)**	55 (12)	55 (11)	>0.99
**BMI, Mean (SD)**	24.5 (10.7)	28.4 (6.3)	0.65
**Cigarette Frequency, n (%)**			0.46
Daily	0 (0%)	2 (29%)	
Occasionally	0 (0%)	0 (0%)	
Never	7 (100%)	5 (71%)	
**Disease Duration Years, Mean (SD)**	11 (6)	14 (12)	>0.99
**ACPA+, n (%)**	6 (75%)	8 (100%)	0.47
**RF+, n (%)**	7 (88%)	8 (100%)	>0.99
**Erosive, n (%)**	0 (0%)	0 (0%)	
**Days To Follow-up, Mean (SD)**	87 (15)	89 (12)	0.92
**Concomitant GCs, n (%)**	6 (75%)	6 (75%)	>0.99

Variable	RA Anti IL6R<br>R<br>N = 8	 NR<br>N = 8	 p-value[1]
**Concomitant MTX, n (%)**	3 (38%)	3 (38%)	>0.99
**Baseline Activity, Median (Q1, Q3)**	5.45 (4.63, 6.28)	5.56 (4.52, 6.02)	>0.99

[1]Fisher's exact test; Wilcoxon rank sum test; Wilcoxon rank sum exact test; NA

Table S1.D: UC Anti-TNF

Variable	UC Anti TNF<br>R<br>N = 8	 NR<br>N = 8	 p-value[1]
**Sex Female, n (%)**	4 (50%)	4 (50%)	>0.99
**Age, Mean (SD)**	39 (15)	46 (19)	0.34
**BMI, Mean (SD)**	22.7 (3.5)	26.3 (6.2)	0.23
**Cigarette Frequency, n (%)**			>0.99
Daily	2 (25%)	1 (13%)	
Occasionally	0 (0%)	1 (13%)	
Never	6 (75%)	6 (75%)	
**Disease Duration Years, Mean (SD)**	6.0 (2.8)	7.0 (7.8)	0.84
**Days To Follow-up, Mean (SD)**	99.1 (2.8)	98.6 (8.6)	0.18
**Concomitant GCs, n (%)**	2 (25%)	2 (25%)	>0.99
**Baseline Activity, Median (Q1, Q3)**	6.50 (5.50, 7.50)	8.00 (4.50, 9.50)	0.63

[1]Fisher's exact test; Wilcoxon rank sum test; Wilcoxon rank sum exact test

Table S1.E: PsA Anti-TNF

Variable	PSA Anti TNF<br>R<br>N = 8	 NR<br>N = 8	 p-value[1]
**Sex Female, n (%)**	4 (50%)	6 (75%)	0.61
**Age, Mean (SD)**	50 (11)	49 (12)	>0.99
**BMI, Mean (SD)**	27.0 (2.8)	24.8 (4.8)	0.54
**Cigarette Frequency, n (%)**			>0.99
Daily	1 (13%)	1 (13%)	
Occasionally	1 (13%)	0 (0%)	
Never	6 (75%)	7 (88%)	
**Disease Duration Years, Mean (SD)**	8.3 (5.7)	3.1 (2.8)	0.052
**Days To Follow-up, Mean (SD)**	85 (9)	88 (9)	>0.99
**Concomitant GCs, n (%)**	3 (38%)	4 (50%)	>0.99
**Baseline Activity, Median (Q1, Q3)**	4.29 (4.04, 4.68)	4.59 (3.40, 5.65)	0.96

[1]Fisher's exact test; Wilcoxon rank sum test; Wilcoxon rank sum exact test

Table S1.F: PS IL12IL23 inhibitor

Variable	PS IL12IL23 inhibitor<br>R<br>N = 8	 NR<br>N = 8	 p-value[1]
**Sex Female, n (%)**	4 (50%)	2 (25%)	0.61
**Age, Mean (SD)**	45 (10)	48 (12)	0.49
**BMI, Mean (SD)**	25.3 (3.8)	28.5 (3.3)	0.10
**Cigarette Frequency, n (%)**			0.20
Daily	3 (38%)	0 (0%)	
Occasionally	0 (0%)	0 (0%)	
Never	5 (63%)	8 (100%)	

	PS IL12IL23 inhibitor		
**Variable**	**R**<br>N = 8	**NR**<br>N = 8	**p-value**[1]
**Disease Duration Years, Mean (SD)**	22 (12)	17 (8)	0.67
**Days To Follow-up, Mean (SD)**	103 (21)	106 (13)	0.92
**Concomitant GCs, n (%)**	0 (0%)	0 (0%)	
**Baseline Activity, Median (Q1, Q3)**	21 (13, 24)	13 (11, 21)	0.46

[1]Fisher's exact test; Wilcoxon rank sum test; Wilcoxon rank sum exact test; NA

Table S1.G: CD Anti TNF

	CD Anti TNF		
**Variable**	**R**<br>N = 8	**NR**<br>N = 8	**p-value**[1]
**Sex Female, n (%)**	5 (63%)	5 (63%)	>0.99
**Age, Mean (SD)**	44 (16)	46 (10)	0.79
**BMI, Mean (SD)**	25.0 (7.4)	23.7 (3.2)	>0.99
**Cigarette Frequency, n (%)**			>0.99
Daily	4 (50%)	4 (50%)	
Occasionally	2 (25%)	1 (13%)	
Never	2 (25%)	3 (38%)	
**Disease Duration Years, Mean (SD)**	7 (7)	11 (11)	0.52
**Days To Follow-up, Mean (SD)**	103 (12)	104 (9)	0.67
**Concomitant GCs, n (%)**	4 (50%)	2 (25%)	0.61
**Baseline Activity, Median (Q1, Q3)**	191 (158, 217)	148 (86, 170)	0.13

[1]Fisher's exact test; Wilcoxon rank sum test; Wilcoxon rank sum exact test

Table S1.H: RA JAK inhibitor

	RA JAK inhibitor		
**Variable**	**R**<br>N = 8	**NR**<br>N = 8	**p-value**[1]
**Sex Female, n (%)**	7 (88%)	8 (100%)	>0.99
**Age, Mean (SD)**	54 (12)	44 (15)	0.10
**BMI, Mean (SD)**	25.4 (5.1)	26.4 (6.2)	0.72
**Cigarette Frequency, n (%)**			>0.99
Daily	1 (13%)	2 (25%)	
Occasionally	0 (0%)	0 (0%)	
Never	7 (88%)	6 (75%)	
**Disease Duration Years, Mean (SD)**	10 (11)	13 (9)	0.37
**ACPA+, n (%)**	7 (100%)	8 (100%)	
**RF+, n (%)**	8 (100%)	8 (100%)	
**Erosive, n (%)**	0 (0%)	0 (0%)	
**Days To Follow-up, Mean (SD)**	90 (11)	84 (7)	0.30
**Concomitant GCs, n (%)**	5 (63%)	7 (88%)	0.57
**Concomitant MTX, n (%)**	1 (13%)	3 (38%)	0.57
**Baseline Activity, Median (Q1, Q3)**	5.50 (5.20, 5.84)	6.35 (4.91, 6.85)	0.37

[1]Fisher's exact test; Wilcoxon rank sum test; Wilcoxon rank sum exact test; NA

Table S1.I: RA Anti TNF

	RA Anti TNF		
**Variable**	**R**<br>N = 8	**NR**<br>N = 8	**p-value**[1]
**Sex Female, n (%)**	8 (100%)	8 (100%)	

RA Anti TNF			
**Variable**	**R**<br>N = 8	**NR**<br>N = 8	**p-value**[1]
**Age, Mean (SD)**	48 (16)	51 (11)	0.64
**BMI, Mean (SD)**	25.3 (4.6)	27.9 (5.3)	0.37
**Cigarette Frequency, n (%)**			>0.99
Daily	2 (25%)	3 (38%)	
Occasionally	0 (0%)	0 (0%)	
Never	6 (75%)	5 (63%)	
**Disease Duration Years, Mean (SD)**	7.8 (7.0)	8.1 (5.6)	0.87
**ACPA+, n (%)**	8 (100%)	8 (100%)	
**RF+, n (%)**	8 (100%)	7 (88%)	>0.99
**Erosive, n (%)**	0 (0%)	0 (0%)	
**Days To Follow-up, Mean (SD)**	89 (13)	88 (17)	0.83
**Concomitant GCs, n (%)**	8 (100%)	8 (100%)	
**Concomitant MTX, n (%)**	5 (63%)	2 (25%)	0.31
**Baseline Activity, Median (Q1, Q3)**	4.56 (4.08, 5.74)	5.07 (3.82, 6.54)	>0.99

[1]NA; Wilcoxon rank sum test; Fisher's exact test

Table S1.J: PS Anti IL17A

PS Anti IL17A			
**Variable**	**R**<br>N = 11	**NR**<br>N = 5	**p-value**[1]
**Sex Female, n (%)**	5 (45%)	4 (80%)	0.31
**Age, Mean (SD)**	53 (14)	58 (12)	>0.99
**BMI, Mean (SD)**	28.7 (5.0)	30.0 (5.0)	0.83
**Cigarette Frequency, n (%)**			0.59
Daily	2 (18%)	2 (40%)	
Occasionally	2 (18%)	0 (0%)	
Never	7 (64%)	3 (60%)	
**Disease Duration Years, Mean (SD)**	30 (21)	13 (10)	0.15
**Days To Follow-up, Mean (SD)**	96 (16)	90 (18)	0.58
**Concomitant GCs, n (%)**	0 (0%)	0 (0%)	
**Baseline Activity, Median (Q1, Q3)**	8.00 (6.00, 10.20)	6.90 (5.30, 7.50)	0.46

[1]Fisher's exact test; Wilcoxon rank sum test; Wilcoxon rank sum exact test; NA

Table S1.K: IBD IL12IL23 inhibitor

IBD IL12IL23 inhibitor			
**Variable**	**R**<br>N = 7	**NR**<br>N = 9	**p-value**[1]
**Sex Female, n (%)**	4 (57%)	4 (44%)	>0.99
**Age, Mean (SD)**	48 (10)	51 (19)	0.79
**BMI, Mean (SD)**	25.5 (5.8)	25.1 (3.6)	0.87
**Cigarette Frequency, n (%)**			>0.99
Daily	0 (0%)	0 (0%)	
Occasionally	0 (0%)	1 (11%)	
Never	7 (100%)	8 (89%)	
**Disease Duration Years, Mean (SD)**	6 (4)	15 (15)	0.24
**Days To Follow-up, Mean (SD)**	103 (11)	104 (7)	>0.99
**Concomitant GCs, n (%)**	1 (14%)	1 (11%)	>0.99
**Baseline Activity, Median (Q1, Q3)**	148 (7, 216)	126 (9, 193)	0.71

[1]Fisher's exact test; Wilcoxon rank sum test; Wilcoxon rank sum exact test

Table S7. Sensitivity analysis results for the MNRS correlation between Anti-TNF and Anti-IL6R. Concomitant OGC = Concomitant treatment with oral glucocorticoids. ConcomitantMTX = Concomitant treatment with Methotrexate. Related to Figure 2.

NR Sig. Adjustment	AntiIL6R NR - AntiTNF Effect r (95%CI)	AntiTNF NR - AntiIL6R Effect r (95%CI)
Default	-0.87 (-0.88, -0.86)	-0.76 (-0.79, -0.72)
SexAge	-0.87 (-0.88, -0.86)	-0.79 (-0.82, -0.77)
ConcomitantMTX	-0.86 (-0.87, -0.85)	-0.52 (-0.59, -0.45)
DaysToFollowup	-0.87 (-0.88, -0.86)	-0.74 (-0.78, -0.71)
ConcomitantOGC	-0.87 (-0.88, -0.86)	-
Disease DurationYears	-0.87 (-0.88, -0.86)	-0.77 (-0.8, -0.73)

Table S13. Clinical information of patients used for the in vitro characterization of anti-TNF effect in PBMCs (Parse single-cell RNASeq). Concomitant GCs = Concomitant treatment with oral glucocorticoids. Related to Figure 5.

	**RA**
**SexFemale, n (%)**	6 (100%)
**Age, Mean (SD)**	52.8 (13.4)
**BMI, Mean (SD)**	24.31 (4.8)
**ACPA positive, n (%)**	6 (100%)
**RF positive, n (%)**	5 (83.3%)
**Cigarette Frequency, n (%)**	
Daily	2 (33.3%)
Occasionally	0 (0%)
Never	4 (66.7%)
**Disease Duration Years, Mean (SD)**	4.6 (3.8)
**Concomitant GCs, n (%)**	6 (100%)
**Disease Activity, Median (Q1, Q3)**	4.65 (4.47, 7.60)

SUPPLEMENTAL INFORMATION

Data S1. Detailed patient eligibility and response criteria

For all cohorts, general patient requirements included having:

- Body Mass Index < 36
- Standardized diagnosis of the corresponding IMID and no other IMID or severe comorbidities such as cancer, cardiovascular disease or diabetes
- No more than one failure to previous biological therapies

For RA-AntiIL6R:

- Considered drugs: Tocilizumab, Sarilumab
- Minimum disease activity score at baseline: DAS28=3.2
- Response criteria: responders were good responders by the EULAR criteria , non-responders were non-responders by the EULAR criteria
- Patients were not considered if they had been previously treated with Rituximab

For RA-AntiTNF:

- Considered drugs: Adalimumab, Certolizumab, Etanercept and Golimumab
- Minimum disease activity score at baseline: DAS28=3.2
- Response criteria: responders were good responders by the EULAR criteria [61], non-responders were non-responders by the EULAR criteria
- Patients were not considered if they had been previously treated with Rituximab, or if they were negative for both rheumatoid factor and ACPA

For RA-JAKi:

- Considered drugs: Tofacitinib, Baricitinib
- Minimum disease activity score at baseline: DAS28=3.2
- Response criteria: responders were good responders by the EULAR criteria [61], non-responders were non-responders by the EULAR criteria
- Patients were not considered if they had been previously treated with Rituximab, or if they were negative for both rheumatoid factor and ACPA

For PsA-AntiTNF:

- Considered drugs: Adalimumab, Certolizumab, Etanercept, Golimumab, Infliximab
- Minimum disease activity score at baseline: DAS28=3.2
- Response criteria: responders were good responders by the EULAR criteria [61], non-responders were non-responders by the EULAR criteria
- Patients were not considered if they had been previously treated with Abatacept

For PsA-AntiIL17A:

- Considered drugs: Secukinumab, Ixekizumab
- Minimum disease activity score at baseline: DAS28=3.2
- Response criteria: responders were good responders by the EULAR criteria [61], non-responders were non-responders by the EULAR criteria
- Patients were not considered if they had been previously treated with abatacept

For PS-AntiIL12/23:

- Considered drugs: Ustekinumab
- Minimum disease activity score at baseline: PASI = 8
- Response criteria: responders met the PASI90 criteria [62], non-responders did not meet PASI75 criteria.

For PS-AntiIL17:

- Considered drugs: Brodalumab, Ixekizumab, Secukinumab
- Response criteria: responders met the PASI75 criteria [62], non-responders did not meet PASI75 criteria.

For CD-AntiTNF:

- Considered drugs: Infliximab, Adalimumab
- Patients should have an inflammatory disease behaviour, and have colonic or ileo-colonic disease location
- Minimum disease activity score at baseline: CDAI [63] of 50
- Response criteria: responders had a decrease in CDAI of at least 70 points and at least 25% in subjects with baseline score greater than or equal to 220

For UC-AntiTNF:

- Considered drugs: Infliximab, Adalimumab
- Minimum disease activity score at baseline: MAYO score [64] of 3
- Response criteria: responders needed to have a decrease of at least 2 points in the MAYO clinical score with MAYO stools score of 0 and global score less than or equal to 1 at follow-up

For IBD-AntiIL12/23:

- Considered drugs: Ustekinumab
- Diagnosis of Crohn's disease or Ulcerative Colitis
- Minimum disease activity score at baseline: MAYO score [64] of 3 for UC and CDAI [63] of 50 for CD
- Response criteria: CD responders had a decrease in CDAI of at least 70 points and at least 25% in subjects with baseline score greater than or equal to 220. UC responders needed to have a decrease of at least 2 points in the MAYO clinical score with MAYO stools score of 0 and global score less than or equal to 1 at follow-up

For SLE-AntiBLyS/BAFF:

- Considered drugs: Belimumab
- Minimum disease activity score at baseline: SELENA-SLEDAI [65] of 4
- Response criteria: responders met the SRI4 criteria [66], non-responders needed to have an improvement less than or equal to 2 points in SELENA-SLEDAI
- Males were excluded

List of participants of the IMID-Consortium

MªAngeles Aguirre Zamorano; Rheumatology Department, Hospital Universitario Reina Sofia; Córdoba, Spain.

Mercedes Alperiz; Rheumatology Department, Hospital Universitario Central de Asturias; Asturias, Spain.

José Luís Andreu; Rheumatology Department, Hospital Universitario Puerta de Hierro; Madrid, Spain.

Manuel Barreiro; Gastroenterology Department, University Hospital of Santiago de Compostela; Santiago de Compostela, Spain.

Isabel Belinchón; Dermatology Department, Hospital General Universitario de Alicante; Alicante, Spain.

Diego Benavente; Rheumatology Department, Hospital Universitari de Bellvitge; Barcelona, Spain.

Francisco Blanco; Rheumatology Department, Complejo Hospitalario Universitario de A Coruña; A Coruña, Spain.

Sara Blanco; Dermatology Department, Hospital Universitario de Salamanca; Salamanca, Spain.

Ricardo Blanco; Rheumatology Department, Hospital Universitario Marqués de Valdecilla; Santander, Spain.

Natalia Borruel; Gastroenterology Department, Hospital Universitari Vall d'Hebron; Barcelona, Spain.

Luis Bujanda; Gastroenterology Department, Hospital Universitario de Donostia; San Sebastián, Spain.

José Luís Cabriada; Gastroenterology Department, Hospital Universitario de Galdakao; Bilbao, Spain.

Jaime Calvo Alén; Rheumatology Department, Hospital Universitario Araaba; Alava, Spain.

Juan Cañete; Rheumatology Department, Fundació Clínic per a la Recerca Biomèdica; Barcelona, Spain.

Jose Manuel Carrascosa; Dermatology Department, Hospital Universitari Germans Trias i Pujol; Badalona, Spain.

Patricia Carreira; Rheumatology Department, Hospital Universitario 12 de Octubre; Madrid, Spain.

Santos Castañeda; Rheumatology Department, Hospital Universitario La Princesa; Madrid, Spain.

Hèctor Corominas; Rheumatology Department, Hospital de la Santa Creu i Sant Pau; Barcelona, Spain.

Josefina Cortés; Rheumatology Department, Hospital Universitari Vall d'Hebron; Barcelona, Spain.

Pablo de la Cueva; Dermatology Department, Hospital Universitario Infanta Leonor; Madrid, Spain.

Esteban Dauden; Dermatology Department, Hospital Universitario La Princesa; Madrid, Spain.

César Díaz; Rheumatology Department, Hospital de la Santa Creu i Sant Pau; Barcelona, Spain.

Eugeni Domènech; Gastroenterology Department, Hospital Universitari Germans Trias i Pujol; Badalona, Spain.

Elvira Díez; Rheumatology Department, Complejo Asistencial Universitario de León; León, Spain.

Alba Erra; Rheumatology Department, Hospital Sant Rafael; Barcelona, Spain.

Maria Esteve Comas; Gastroenterology Department, Hospital Universitari Mútua de Terrasa; Terrassa, Spain.

Benjamín Fernández; Rheumatology Department, Hospital Clínico San Carlos; Madrid, Spain.

Antonio Fernández-Nebro; Rheumatology Department, Hospital Regional Universitario Carlos Haya; Málaga, Spain.

Eduardo Fonseca; Dermatology Department, Complejo Hospitalario Universitario de A Coruña; A Coruña, Spain.

Mercedes Freire; Rheumatology Department, Complejo Hospitalario Universitario de A Coruña; A Coruña, Spain.

Esther García-Planella; Gastroenterology Department, Hospital de la Santa Creu i Sant Pau; Barcelona, Spain.

Vicenç García Patos; Dermatology Department, Hospital Universitari Vall d'Hebron; Barcelona, Spain.

Charo García de Vicuña; Rheumatology Department, Hospital Universitario La Princesa; Madrid, Spain.

María Jesús García Villanueva; Rheumatology Department, Hospital Universitario Ramón y Cajal

María Luz García Vivar; Rheumatology Department, Hospital Universitario de Basurto; Bilbao, Spain.

Fernando Gomollón García; Gastroenterology Department, Hospital Clínico Universitario Lozano Blesa; Zaragoza, Spain.

Carlos González; Rheumatology Department, Hospital Universitario Gregorio Marañón; Madrid, Spain.

Maria José González; Rheumatology Department, Hospital Universitario Quiron Dexeus; Barcelona, Spain.

Isidoro González; Rheumatology Department, Hospital Universitario La Princesa; Madrid, Spain.

Jordi Gratacós; Rheumatology Department, Hospital Parc Taulí; Sabadell, Spain.

Jordi Guardiola; Gastroenterology Department, Hospital Universitari de Bellvitge; Barcelona, Spain.

Iván Guerra; Gastroenterology Department, Hospital Universitario de Fuenlabrada; Madrid, Spain.

Ana Gutiérrez; Gastroenterology Department, Hospital General Universitario de Alicante; Alicante, Spain.

Enrique Herrera; Dermatology Department, Hospital Universitario Virgen de la Victoria; Málaga, Spain.

Susana Holgado; Rheumatology Department, Hospital Universitari Germans Trias i Pujol; Badalona, Spain.

Iago Rodríguez-Lago; Gastroenterology Department, Hospital Universitario de Galdakao; Bilbao, Spain.

Eva Iglesias; Gastroenterology Department, Hospital Universitario Reina Sofía; Córdoba, Spain.

Beatriz Joven; Rheumatology Department, Hospital Universitario 12 de Octubre

Ana Laiz; Rheumatology Department, Hospital de la Santa Creu i Sant Pau; Barcelona, Spain.

Laura López; Rheumatology Department, Hospital Sant Rafael; Barcelona, Spain.

Antonio Lopez-Sanromán; Gastroenterology Department, Hospital Universitario Ramón y Cajal; Madrid, Spain.

José Luís López Estebaranz; Dermatology Department, Hospital Universitario Fundación Alcorcón; Madrid, Spain.

Marta Loredo Martinez; Rheumatology Department, Hospital Universitario Central de Asturias; Asturias, Spain.

Berta Magallares; Rheumatology Department, Hospital de la Santa Creu i Sant Pau; Barcelona, Spain.

José Luís Marenco de la Fuente; Rheumatology Department, Hospital Universitario Virgen de Valme; Sevilla, Spain.

Nacho Marín; Gastroenterology Department, Hospital General Universitario Gregorio Marañón; Madrid, Spain.

Carlos Marras; Rheumatology Department, Hospital Universitario Virgen de la Arrixaca; Murcia, Spain.

Andrea Pluma; Rheumatology Department, Hospital Universitari Vall d'Hebron; Barcelona, Spain.

Mariló Martín Arranz; Gastroenterology Department, Hospital Universitario La Paz; Madrid, Spain.

Julia Martínez Barrio; Rheumatology Department, Hospital Universitario Gregorio Marañón; Madrid, Spain.

Víctor Martínez Taboada; Rheumatology Department, Hospital Universitario Marqués de Valdecilla; Santander, Spain.

Lourdes Mateo; Rheumatology Department, Hospital Universitari Germans Trias i Pujol; Badalona, Spain.

Juan Luís Mendoza; Gastroenterology Department, Hospital Clínico San Carlos; Madrid, Spain.

Francisco Mesonero; Gastroenterology Department, Hospital Universitario Ramón y Cajal; Madrid, Spain.

Joan Miquel Nolla; Rheumatology Department, Hospital Universitari de Bellvitge; Barcelona, Spain.

Jordi Monfort; Rheumatology Department, Hospital Universitari Parc de Salut Mar; Barcelona, Spain.

Carlos Montilla; Rheumatology Department, Hospital Universitario Virgen de la Vega; Salamanca, Spain.

Estefania Moreno; Rheumatology Department, Hospital Comarcal Alt Penedès; Vilafranca, Spain.

David Moreno Ramírez; Dermatology Department, Hospital Universitario Virgen Macarena; Sevilla, Spain.

Santiago Muñoz; Rheumatology Department, Hospital Universitario Infanta Sofía / Hospital Universitario La Paz; Madrid, Spain.

Fernando Muñoz; Gastroenterology Department, Hospital Universitario de Salamanca; Salamanca, Spain.

Antonio Naranjo; Rheumatology Department, Hospital Universitario de Gran Canaria Dr. Negrín; Gran Canarias, Spain.

Natividad Oreiro-Villar; Rheumatology Department, Complejo Hospitalario Universitario de A Coruña; A Coruña, Spain.

Pilar Nos Mateu; Gastroenterology Department, Hospital Universitario y Politécnico La Fe; Valencia, Spain.

Jaime Notario; Dermatology Department, Hospital Universitari de Bellvitge; Barcelona, Spain.

Ana Ortiz; Rheumatology Department, Hospital Universitario La Princesa; Madrid, Spain.

Pedro Zarco; Rheumatology Department, Fundación Hospital Alcorcón; Madrid, Spain.

Jose María Pego; Rheumatology Department, Hospital do Meixoeiro; Vigo, Spain.

Javier P. Gisbert; Gastroenterology Department, Hospital Universitario de la Princesa; Madrid, Spain.

José Javier Pérez-Venegas; Rheumatology Department, Hospital Universitario de Jerez de la Frontera; Cádiz, Spain.

Jordi Monfort; Rheumatology Department, Hospital Universitari Parc de Salut Mar; Barcelona, Spain.

José Antonio Pinto-Tasende; Rheumatology Department, Complejo Hospitalario Universitario de A Coruña; A Coruña, Spain.

Lluís Puig; Dermatology Department, Hospital de la Santa Creu i Sant Pau; Barcelona, Spain.

Ramon Pujol; Dermatology Department, Hospital Universitari Parc de Salut Mar; Barcelona, Spain.

Rubén Queiró; Rheumatology Department, Hospital Universitario Central de Asturias; Oviedo, Spain.

Delia Reina; Rheumatology Department, Hospital Moisès Broggi; Sant Joan Despí, Spain.

Elena Ricart; Gastroenterology Department, Fundació Clínic per a la Recerca Biomèdica; Barcelona, Spain.

Pilar del Río-Martínez; Rheumatology Department, Centro de Especialidades Médicas Inocencio Jiménez; Zaragoza, Spain.

Raquel Rivera; Dermatology Department, Hospital Universitario 12 de Octubre; Madrid, Spain.

Anne Riveros; Rheumatology Department, Hospital Universitari Germans Trias i Pujol; Badalona, Spain.

Esther Rodríguez; Rheumatology Department, Hospital Universitario 12 de Octubre; Madrid, Spain.

Daniel Roig; Rheumatology Department, Hospital Moisès Broggi; Sant Joan Despí, Spain.

Mónica Roncero Riesco; Dermatology Department, Hospital Universitario de Salamanca; Salamanca, Spain.

Iñigo Jesús Rua-Figueroa; Rheumatology Department, Hospital Universitario de Gran Canaria Doctor Negrín; Las Palmas de Gran Canaria, Spain.

Tarek Salman; Rheumatology Department, Hospital Universitari Parc de Salut Mar; Barcelona, Spain.

Georgina Salvador; Rheumatology Department, Hospital Universitari Mútua de Terrasa; Terrassa, Spain.

Simón Sánchez; Rheumatology Department, Hospital General La Mancha Centro; Ciudad Real, Spain.

Jose Luís Sánchez Carazo; Dermatology Department, Hospital General Universitario de Valencia; Valencia, Spain.

Raimon Sanmartí; Rheumatology Department, Fundació Clínic per a la Recerca Biomèdica; Barcelona, Spain.

Mónica Sierra; Gastroenterology Department, Complejo Asistencial Universitario de León; León, Spain.

Carles Tomàs; Rheumatology Department, Hospital comarcal d'Amposta; Amposta, Spain.

Jesús Tornero; Rheumatology Department, Hospital Universitario Guadalajara; Guadalajara, Spain.

Juan Carlos Torre-Alonso; Dermatology Department, Faculty of Medicine, Universidad de Oviedo; Oviedo, Spain.

Paloma Vela; Rheumatology Department, Hospital General Universitario de Alicante; Alicante, Spain.

Maribel Vera; Gastroenterology Department, Hospital Universitario Puerta de Hierro; Madrid, Spain.
