# Supplementary material for: Uncovering combination therapies for immune-mediated inflammatory diseases through systems biology analysis on longitudinal patient data
Source: Cell Rep Med. 2026 Sep 11;7(10):103026. doi: 10.1016/j.xcrm.2026.103026 (PMC13710146; doi:10.1016/j.xcrm.2026.103026)
Supplement: Supplementary file 2 [file mmc17.pdf]

Cell Reports Medicine

Article

Uncovering combination therapies for immune-mediated inflammatory diseases through systems biology analysis on longitudinal patient data

Graphical abstract

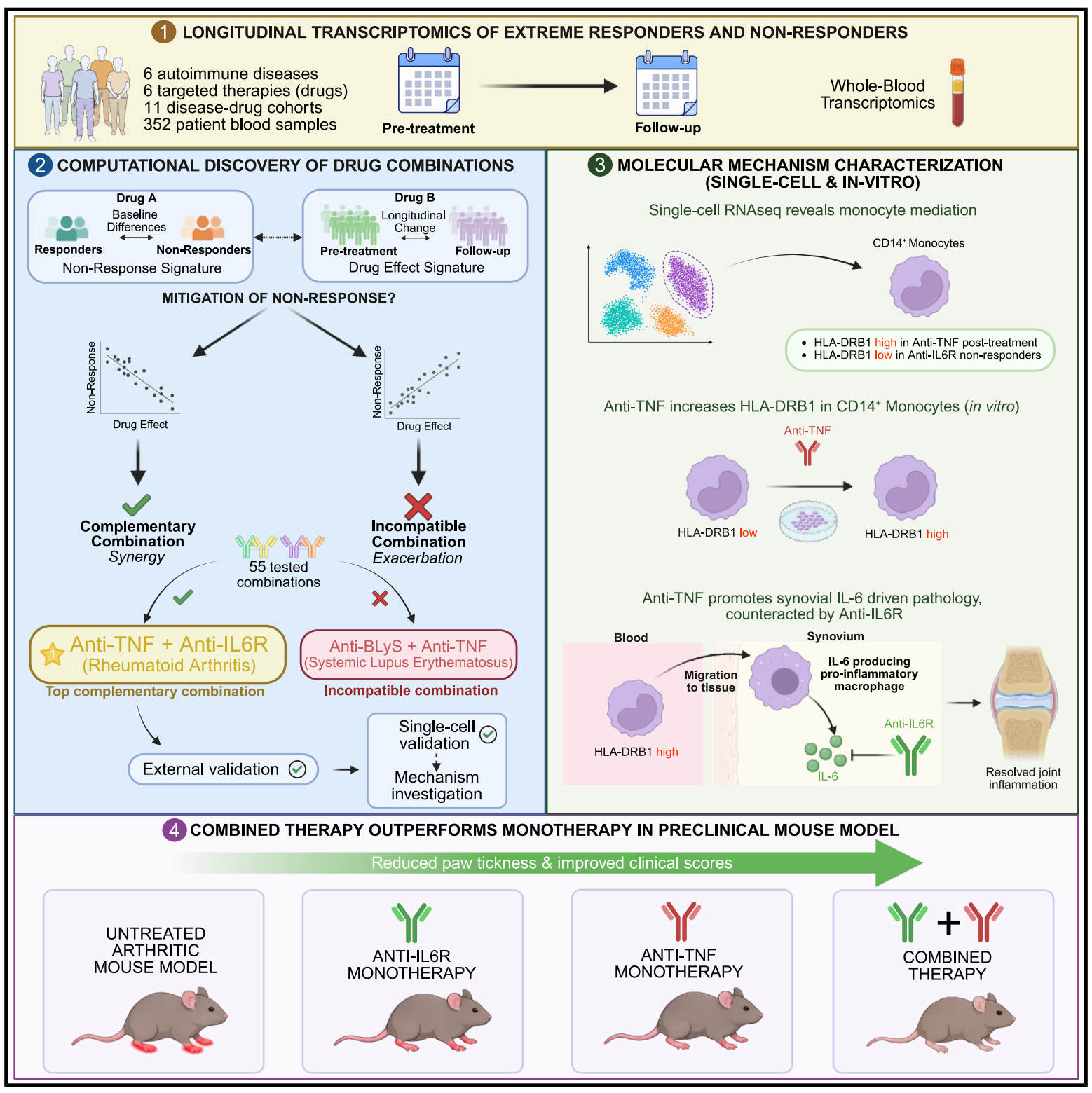

Highlights

- A systems approach prioritizes drug combinations from longitudinal patient data
- Anti-TNF and anti-IL-6R therapies are complementary in rheumatoid arthritis
- Single-cell analysis localizes the drug complementarity to $CD14^+$ monocytes
- In mice, combined anti-TNF/anti-IL-6R therapy outperforms monotherapy

Authors

Sergio H. Martínez-Mateu, Yolanda Guillén, Edgar Angelats, ..., Pere Santamaria, Sara Marsal, Antonio Julià

Correspondence

tonijuliacano@gmail.com

In brief

Martínez-Mateu et al. develop a computational framework that uses longitudinal blood transcriptomes from patients with immune-mediated inflammatory diseases to prioritize combination therapies. They identify anti-TNF plus anti-IL-6R as complementary in rheumatoid arthritis, localize the effect to $CD14^+$ monocytes, and validate the combination in mice, nominating candidates for clinical testing.

Martínez-Mateu et al., 2026, Cell Reports Medicine *7*, 103026
October 20, 2026 © 2026 The Author(s). Published by Elsevier Inc.
https://doi.org/10.1016/j.xcrm.2026.103026

CellPress

Cell Reports Medicine

CellPress
OPEN ACCESS

Article

Uncovering combination therapies for immune-mediated inflammatory diseases through systems biology analysis on longitudinal patient data

Sergio H. Martínez-Mateu,[1,2] Yolanda Guillén,[1] Edgar Angelats,[3] María A. López-Lasanta,[4] Stefania Madonna,[5] Laura Jiménez-Gracia,[2,6] Ivan Rodriguez-Nunez,[7] Damiana Álvarez-Errico,[1] Adrià Aterido,[8] Raül Tortosa,[8] Max Ruiz,[6] Pau Serra,[3] Juan D. Cañete,[3,9] Jose M. Carrascosa,[10] Eugeni Domènech,[11] Javier P. Gisbert,[12] Jesús Tornero,[13] IMID-Consortium[19] Britta Siegmund,[14] Giampiero Girolomoni,[15] Ernest H.S. Choy,[16] Richard M. Myers,[7] Holger Heyn,[2,6,17] Pere Santamaria,[3,18] Sara Marsal,[4,8] and Antonio Julià[8,20,*]

[1]IMIDomics, Inc., Barcelona, Spain
[2]Universitat de Barcelona (UB), Barcelona, Spain
[3]Institut d'Investigacions Biomèdiques August Pi i Sunyer (IDIBAPS), Barcelona, Spain
[4]Hospital Universitari Vall d'Hebron, Department of Rheumatology, Barcelona, Spain
[5]Laboratory of Experimental Immunology, Istituto Dermopatico dell'Immacolata-Istituto di Ricovero e Cura a Carattere Scientifico, Rome, Italy
[6]Centro Nacional de Análisis Genómico (CNAG), Barcelona, Spain
[7]HudsonAlpha Institute for Biotechnology, Huntsville, AL, USA
[8]Rheumatology Research Group, Vall d'Hebron Research Institute, Barcelona, Spain
[9]Fundació Clínic per a la Recerca Biomèdica, Department of Rheumatology, Barcelona, Spain
[10]Hospital Universitari Germans Trias i Pujol, Department of Dermatology, Badalona, Spain
[11]Hospital Universitari Germans Trias i Pujol, Department of Gastroenterology, and Centro de Investigación Biomédica en Red de Enfermedades Hepáticas y Digestivas (CIBEREHD), Badalona, Spain
[12]Hospital Universitario de La Princesa, Department of Gastroenterology, Instituto de Investigación Sanitaria Princesa (IIS-Princesa), Universidad Autónoma de Madrid (UAM), and Centro de Investigación Biomédica en Red de Enfermedades Hepáticas y Digestivas (CIBEREHD), Madrid, Spain
[13]Hospital General Universitario de Guadalajara, Department of Rheumatology, Guadalajara, Spain
[14]Charité-Universitätsmedizin Berlin, Department of Gastroenterology, Berlin, Germany
[15]Department of Medicine, Section of Dermatology, University of Verona, Verona, Italy
[16]CREATE Centre, Section of Rheumatology, Division of Infection and Immunity, Cardiff University School of Medicine, Cardiff, UK
[17]ICREA, Barcelona, Spain
[18]Department of Microbiology, Immunology and Infectious Diseases, Cumming School of Medicine, University of Calgary, Calgary, AB, Canada
[19]Further details can be found in the supplemental information
[20]Lead contact
*Correspondence: tonijuliacano@gmail.com
https://doi.org/10.1016/j.xcrm.2026.103026

SUMMARY

While targeted therapies have reshaped the clinical management of immune-mediated inflammatory diseases (IMIDs), primary non-response remains a major obstacle for many patients. Combining two targeted therapies is an emerging strategy to overcome this therapeutic ceiling, but the number of possible drug pairs makes prioritization difficult. For this objective, we present mitigation of non-response signature (MNRS), a computational approach that uses longitudinal blood transcriptomic data from six IMIDs treated with different targeted therapies to identify the most promising drug combinations. The approach identifies complementary pairs of biologic agents and small molecules, as well as potentially incompatible pairs. In rheumatoid arthritis, anti-TNF and anti-interleukin 6 receptor therapy emerges as highly complementary; single-cell analysis localizes this effect to $CD14^+$ monocytes, and a collagen-induced arthritis mouse model confirms that the combination outperforms monotherapy. These findings show that longitudinal patient data help prioritize drug combinations for clinical testing across immune-mediated diseases.

INTRODUCTION

Since the first introduction of tumor necrosis factor (TNF) inhibitors (anti-TNFs) in the late 1990s, there has been significant progress in the development of targeted therapies to treat immune-mediated inflammatory diseases (IMIDs).[1] However, while this drug development has improved the global outcomes of IMIDs compared to broad-spectrum immune modulators, there

© 2026 The Author(s). Published by Elsevier Inc.
This is an open access article under the CC BY license (http://creativecommons.org/licenses/by/4.0/).

are still many patients who do not benefit from targeted drug interventions.[2] Remission rates during the first year are rarely higher than 30%,[3–5] and even for those patients initially remitting, drug efficacy is eventually lost.[6] Thus, there is a major need to break this therapeutic ceiling in IMIDs. An emerging therapeutic strategy involves the combination of existing drugs that complement each other and enable additive or synergistic effects that can allow a larger proportion of patients to reach sustained remission.[7] Clinical studies are costly and time-consuming and therefore, the current challenge is to identify which drug combinations should be prioritized for testing in patients.

IMIDs share common pathogenic mechanisms and, consequently, targeted therapies have shown to be effective across different diseases.[1] With the increasingly large number of advanced therapies that are being approved every year, the number of potential combinations that could be considered is so high that it makes a comprehensive evaluation through clinical trials unrealistic. In oncology, several large-scale perturbation resources—such as Connectivity Map[8] and LINCS[9]—systematically measure transcriptional or viability responses to thousands of compounds across diverse cell lines. These datasets provide harmonized drug-effect signatures at a scale that enables data-driven discovery of drug combinations using approaches such as the signature reversion principle (SRP).[10–12] The systemic nature of IMIDs, however, does not allow this type of direct evaluation of drug efficacy. Alternatively, using large numbers of animals to evaluate all possible combinations raises major ethical concerns. Thus, there is a need to develop an *in silico* approach that can help prioritize drug combinations for IMIDs.

To address this limitation, we have developed an approach named "mitigation of non-response signature" (MNRS). MNRS is a systems biology approach based on the SRP,[13] which traditionally relies on comparing a disease signature to *in vitro* drug-effect signatures derived from perturbation screens. Because such perturbation datasets are not available for IMIDs, we instead leverage patient-derived *in vivo* signatures. In the SRP framework, a drug is predicted to be beneficial when it antagonizes the disease signature and shifts it toward a homeostatic state. Using this approach, we previously identified abatacept, an anti-CD80/86 blocker, as a candidate therapy for severe COVID-19.[14] Clinical evaluation of abatacept in hospitalized severe COVID-19 patients later confirmed the efficacy of this drug to reduce mortality and increase the probability of recovery.[15]

Here, we extend this systems biology framework to the problem of identifying optimal drug combinations for IMIDs. Instead of using a disease signature obtained from comparing cases and controls, here we used the signature of non-response to a specific targeted therapy using RNA-Seq from whole blood. Drug-effect signatures were obtained from the analysis of longitudinal transcriptomic data from patients with six different IMIDs treated with different therapies. Using this approach, we identified drug pairs showing a high level of complementarity, when each drug strongly antagonizes the non-response signature of the other, as well as drug pairs showing potential incompatibility. One of the strongest drug combinations was anti-TNF and anti-IL-6R for the treatment of rheumatoid arthritis (RA). Using single-cell RNA-sequencing (scRNA-Seq), we validated this complementarity, and we identified $CD14^+$ monocytes as a key cell type for mediating the combinatorial effect. Testing on a murine model for the disease corroborated the greater efficacy of the drug combination in concurrent administration compared to monotherapy. We report an approach for the prioritization of combinatorial therapies in IMIDs and provide a proof-of-concept study demonstrating that the combination of anti-TNF and anti-IL-6R can be highly beneficial for RA.

RESULTS

A systems biology approach for drug combination prioritization in IMIDs using longitudinal transcriptomic data from patients

We developed a systems biology approach based on the SRP to identify drug pairs with the highest potential for complementary effect across IMIDs. Our approach is based on two main components: (1) the disease-specific signature of non-response to a given drug, referred to as the non-response signature, and (2) the longitudinal *in vivo* effect of each therapy, referred to as the drug effect signature, as illustrated in Figure 1A. The non-response signature is obtained by comparing the gene expression profile of non-responders to that of responders at baseline. The drug effect signature is captured by comparing the gene expression profile after 3–4 months of treatment of each drug (follow-up) against the gene expression profile at baseline. By contrasting these two signatures, we can determine the extent to which the effect of one drug antagonizes the signature of non-response to a different drug (Figure 1B). We term this strategy "mitigation of non-response signature" (MNRS).

To obtain the two signatures that compose the MNRS, we generated whole blood longitudinal transcriptional profiles of 176 patients from six IMIDs starting a targeted therapy. In total, 352 blood samples were taken at the beginning of treatment and at the time of assessment of the first clinical response (Figure 1A). Patient selection was based on the presence of highly distinctive clinical outcomes, including patients representative of very favorable response (i.e., responders) and patients showing no improvement or even worsening of disease activity (i.e., non-responders). A total of 11 disease and therapy cohorts were selected, each consisting of 16 patients divided into two groups of highly opposed therapeutic outcomes (Table 1). Table S1 summarizes the clinical and epidemiological features of each of the cohorts. Responder and non-responder groups were matched for potential confounding variables (STAR Methods). To reduce heterogeneity related to prior treatment exposure, eligible patients were either naive to targeted therapies or with no more than one failure to a previous targeted therapy (Data S1: patient selection criteria). A total of six targeted therapies were included, five of which were biological agents targeting a cytokine or its receptor: anti-tumor necrosis factor (anti-TNF), anti-p40 subunit (shared by interleukins 12 and 23; anti-IL-12/23), anti-interleukin 17A cytokine/receptor (anti-IL-17A), anti-interleukin 6 receptor (anti-IL-6R), anti-B lymphocyte stimulator (anti-BLyS), and a small molecule Janus kinase inhibitor (JAKi). The longitudinal data from the $n = 352$ transcriptomes were used to generate the drug effect and drug non-response signatures (Figure 1A).

Based on the computed signatures, a total of 55 candidate drug combinations were evaluated using the MNRS analysis

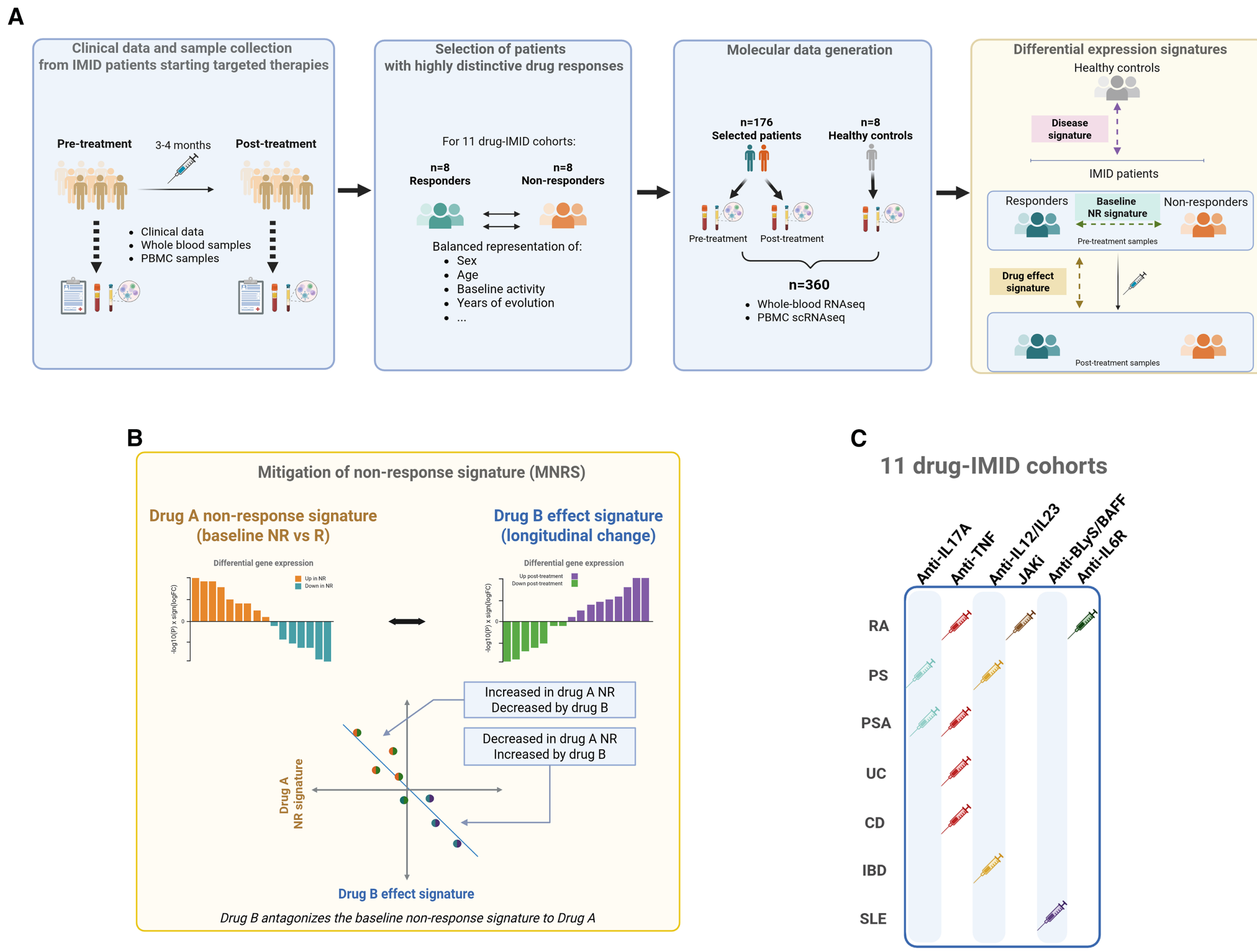

Figure 1. MNRS framework to prioritize optimal drug combinations for IMIDs

(A) Schematic representation of the study design. Left: pre-treatment and post-treatment samples were obtained from a large pool of IMID patients undergoing targeted therapies. Left-center: patients who were good responders or clearly drug-resistant were selected. Right-center: total samples included for data generation, including healthy controls. Right: two types of transcriptomic signatures were derived by differential expression analysis from the generated molecular data.

(B) Overview of the main principle underlying the MNRS drug combination prioritization framework.

(C) Illustration of the 11 drug-disease combinations included in this study, for which longitudinal transcriptomic data were generated. RA, rheumatoid arthritis; PS, psoriasis; PSA, psoriatic arthritis; UC, ulcerative colitis; CD, Crohn's disease; SLE, systemic lupus erythematosus; IBD, inflammatory bowel disease. See also Table S1.

(Table S1 = baseline characteristics of the cohorts.)

(Figure 2A). A total of seven drug combinations showed strong evidence for complementarity (Pearson $r < -0.7$; $p < 5.2e-16$). Five drug combinations showed moderate evidence of complementarity (Pearson $-0.7 < r < -0.5$; $p < 5.1e-16$). Two drug combinations, anti-BLyS-anti-TNF and anti-BLyS-anti-IL-6R both in SLE, showed a strong positive correlation (Pearson $r > 0.8$; $p < 1e-323$). This result is indicative that a large fraction of the genes associated with non-response to anti-BLyS in SLE are exacerbated by either of these candidate combination therapies. These results suggest that both anti-IL-6R and anti-TNF therapies are unlikely to be synergistic in combination with anti-BLyS and may even impair the efficacy of this targeted drug in SLE. Four additional drug combinations showed moderate evidence of exacerbation (Pearson $0.7 > r > 0.5$; p value $< 2.1e-13$). The MNRS analysis identified two distinctively strong antagonisms, one between the longitudinal effect of anti-TNF and the signature of non-response to anti-IL-6R in RA ($r = -0.87$, 95% CI [-0.88, -0.86], Figure 2A; Table S2), and the other between the longitudinal effect of anti-BLyS and the non-response to anti-IL-6R in RA ($r = -0.88$, 95% CI [-0.89, -0.86], Figure 2A; Table S2). These findings were reinforced by the proportion of genes that were antagonized against those that were exacerbated by each of the two complementary therapies (98.9% mitigated and only 1.1% exacerbated by anti-TNF; 97.4% mitigated and 2.6% exacerbated by anti-BLyS; Figure 2B; Table S3). Anti-IL-6R and anti-TNF combination therapy in RA was further supported by the reverse analysis, showing that the longitudinal profile of anti-IL-6R significantly opposes the non-response signature to

Table 1. Overview of the 11 cohorts included in DoCTIS

Disease	Drug	Total patients (*n*)	Responders (R)	Non-responders (NR)
Rheumatoid arthritis (RA)	anti-TNF	16	8	8
	anti-IL-6R	16	8	8
	JAK inhibitor	16	8	8
Psoriatic arthritis (PsA)	anti-TNF	16	8	8
	anti-IL17A	16	8	8
Psoriasis (PS)	anti-IL12/IL23	16	8	8
	anti-IL17A	16	11	5
Crohn's disease (CD)	anti-TNF	16	8	8
Ulcerative colitis (UC)	anti-TNF	16	8	8
Inflammatory bowel disease (IBD)	anti-IL12/IL23	16	7	9
Systemic lupus erythematosus (SLE)	anti-BlyS/BAFF	16	8	8

anti-TNF in RA (r = −0.76, 95% CI [-0.79, -0.72], 91.2% mitigated and 8.8% exacerbated). Overall, these findings demonstrate that MNRS effectively identifies drug combinations with potential therapeutic benefits in IMIDs while also highlighting combinations that may lead to detrimental interactions, thereby guiding informed prioritization of treatment strategies for clinical evaluation.

Characterization and validation of the anti-TNF and anti-IL-6R combination in RA

Given the robust evidence supporting the combination of anti-TNF and anti-IL-6R therapies for the treatment of RA, we prioritized this drug pair for in-depth investigation. We first analyzed the expression of the genes encoding the targets of these therapies, *IL6R* and *TNF*, along with the key components of their signaling pathways, including *TNFRSF1A*, *TNFRSF1B*, and *ADAM17* for the TNF pathway, and *IL6*, *IL6ST*, and *ADAM10* for the IL-6 pathway (Figures S1A and S1B). The two ADAM proteases that cleave and activate IL-6 and TNF were found to be overexpressed in anti-IL-6R non-responders (unadjusted $p < 0.05$; Figure S1A) and significantly downregulated by *in vivo* treatment with anti-TNF therapy (unadjusted $p < 0.001$; Figure S1A). We next evaluated the antagonism at the pathway level. For this, the gene-level signatures were transformed to signatures of enrichment scores of biological processes (Figure S2; Tables S4 and S5, STAR Methods). The MNRS between anti-TNF and anti-IL-6R in RA patients was also found to be strong at the pathway level (r = −0.91 95% CI [−0.95, −0.91] and r = −0.93 95% CI [-0.95, -0.89]; Figures 2C and 2D). The number of anti-IL-6R pathways associated with non-response that were antagonized by anti-TNF were of similar size of the anti-TNF pathways associated with non-response mitigated by anti-IL-6R therapy (128 and 103 pathways, respectively; Table S6; Figures 2C and 2D). The pathways enriched in the anti-IL-6R non-response signature and exclusively addressed by the anti-TNF longitudinal effect (*n* = 59), were found to be heterogeneous but with a predominance of metabolic processes and immune processes like TCR signaling (Figure S3). Therapy with anti-IL-6R also antagonized a variety of biological processes associated anti-TNF non-response (*n* = 57), with a predominance of gene ontologies related with cell division but also with strong opposition to immunological pathways, particularly related to viral response. Together, these results support that anti-TNF and anti-IL-6R complement each other at the functional level.

We next aimed to replicate the observed complementarity between anti-TNF and anti-IL-6R therapies in RA. We identified two transcriptional studies performed in Japanese RA patient cohorts from which we could estimate the non-response and longitudinal drug effect signatures necessary for the MNRS computation. For the former we used GSE78068 dataset, which includes baseline whole blood transcriptomic profiles from *n* = 98 responders and *n* = 42 non-responders to anti-TNF (infliximab), and the baseline profiles of *n* = 30 responders and *n* = 8 non-responders to anti-IL-6R (tocilizumab).[16] For the longitudinal drug-effects, we used GSE93272 dataset, which includes the longitudinal transcriptomic data of *n* = 18 RA patients undergoing therapy with infliximab and of *n* = 13 RA patients undergoing therapy with tocilizumab.[17] After computing the MNRS, we found that the reciprocal antagonism between anti-TNF and anti-IL-6R therapies was significantly validated in the independent patient cohorts in both directions (Figure S4, r = −0.71, 95% CI [-0.81, −0.57] and r = −0.73 95% CI [-0.83, -0.58]). Consistent strong negative correlations were also found when contrasting the signatures from the independent cohort with the signatures generated in our patient cohorts (Figures S4A and S4B). These reciprocal antagonistic relationships remained robust after adjusting the baseline non-response signatures for disease duration, target treatment duration, oral glucocorticoid use, and methotrexate use (Table S7). Pathway-level analysis further supported this result, revealing solely antagonistic interactions with biological processes exacerbated by the complementary drug (Figures S5A and S5B). Collectively, these findings demonstrate that anti-TNF and anti-IL-6R therapies are highly complementary and combining the two drugs could significantly improve therapeutic efficacy in RA.

Single-cell MNRS demonstrates the utility of the framework and identifies $CD14^+$ monocytes as mediators of anti-TNF/anti-IL-6R complementarity

To further support anti-TNF and anti-IL-6R combination for the treatment of RA, we analyzed the complementarity of the two drugs at the single-cell level using the MNRS approach.

CellPress
OPEN ACCESS

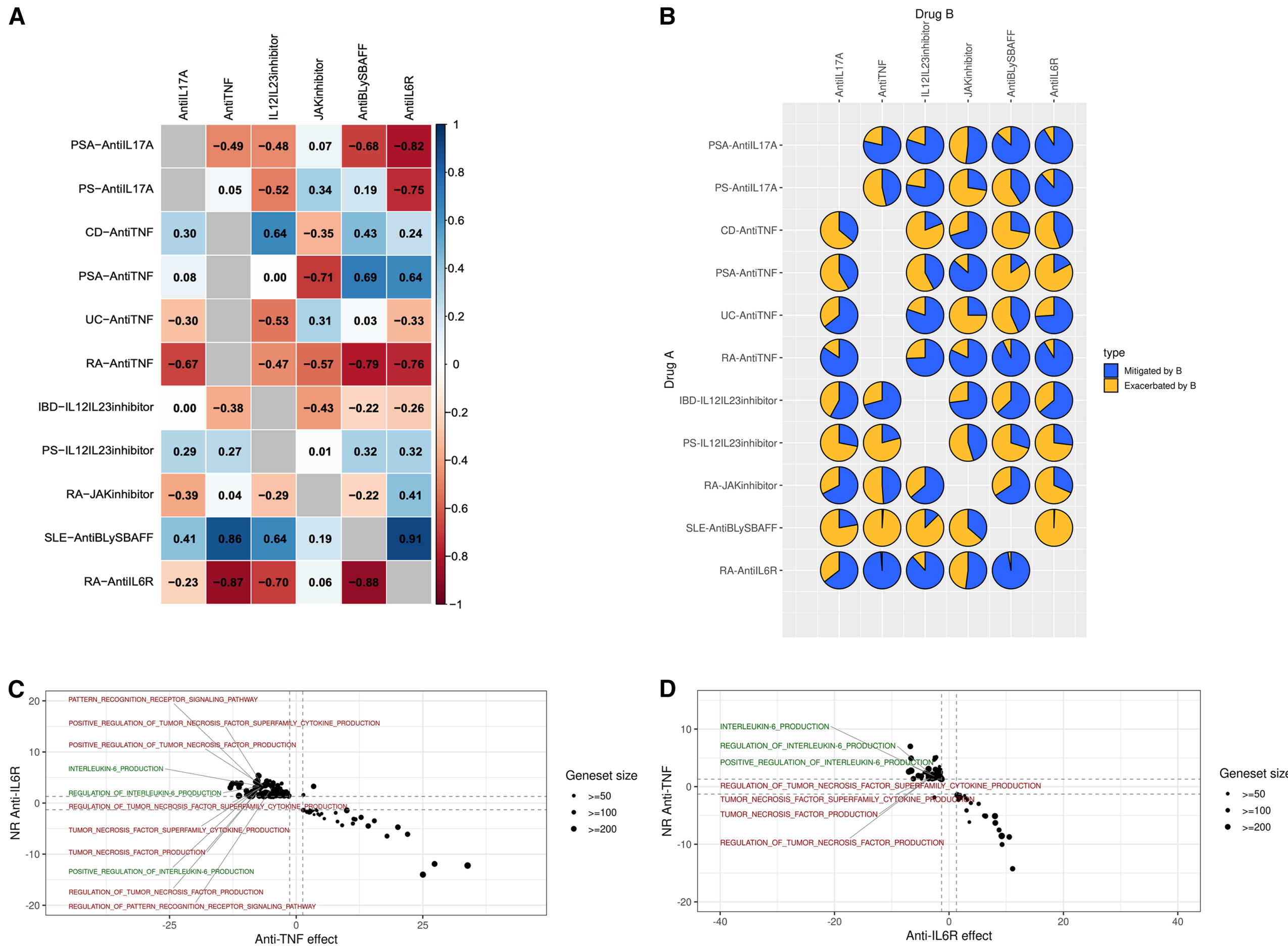

Figure 2. Drug combinatorial analysis in whole blood bulk RNA-seq based on the mitigation of the MNRS

(A) Gene-level correlation coefficients between non-response signatures (rows) and drug effect signatures (columns). Pearson correlations were computed using genes significant at $p < 0.05$ in both signatures. A negative correlation indicates that the effect of the drug in the column tends to reverse the signature of non-response in the drug-disease cohort of the row.

(B) Non-response signature reversal percentages. All the non-response genes significant at $p < 0.05$ in the drug-disease cohort of the row are categorized based on the drug effect of the drug in the column. Antagonized by A: genes that are reversed by the drug effect. Exacerbated by A: genes that are exacerbated by the drug effect.

(C and D) Pathway-level antagonization between anti-TNF and anti-IL-6R non-response signature in RA patients (C) and between anti-IL-6R effect and anti-TNF non-response signature in RA patients (D). Green: IL-6-related pathways. Red: TNF-related pathways. Biological replicates are individual patients; each drug-disease cohort comprises 16 patients (8 responders, 8 non-responders), except PS-anti-IL-17 and IBD-anti-IL-12/23 (see STAR Methods). See also Figures S1–S5 and Tables S2–S7.

scRNA-seq data were generated from peripheral blood mononuclear cells (PBMCs) from 48 RA patients and 8 healthy controls. Patients were analyzed at baseline and at the week of clinical response, resulting in a total of 104 samples. After quality control, a total of 590,872 cells were annotated into 29 different circulating immune cell types (Figure 3A). All cell types were present in all donors (Figure 3B), except for the less frequent cDC1, and the recently characterized ASDCs (present in 98.4% and 87.5% of donors, respectively). Treatment effect led to changes in cell composition in all three therapies (Figure S6).

To determine whether MNRS could provide cell-type-resolved information beyond whole-blood transcriptomic prioritization, we applied the same conceptual framework to the longitudinal scRNA-seq dataset. We applied the MNRS analysis to each of the 29 immune cell types and across the three RA drug combinations (anti-TNF versus anti-IL-6R, anti-TNF versus JAKi, and anti-IL-6R versus JAKi). In line with the bulk blood transcriptomic analysis, we found a strong antagonism between the longitudinal signature of anti-TNF and the non-response signature to anti-IL-6R (Figure 3C; Figures S7–S9; Table S8). The antagonism was highly significant in the classical $CD14^+$ monocyte compartment, including $CD14^+$ monocytes (p = 1e−35), $CD14^+$ $CCL2^{Hi}$ monocytes (p = 1e−9), and $CD14^+$ $DAB2^{Hi}$ monocytes (p = 1e−24) (Figure 3C; Table S9). Across these three cell types, HLA-DRB1 withstood as the most significantly antagonized gene. $CD14^+$ monocytes from anti-IL-6R non-responder patients

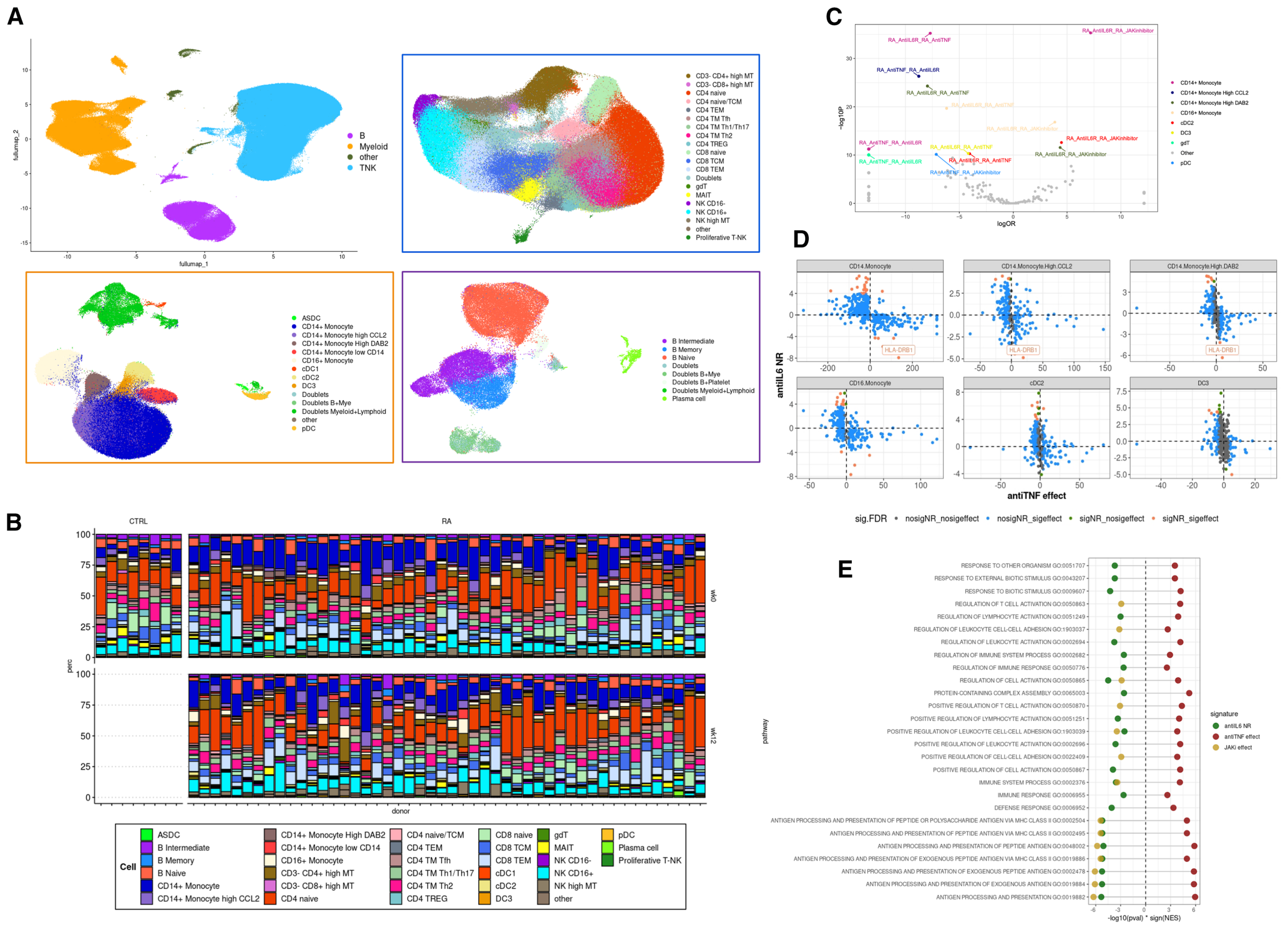

Figure 3. MNRS analysis using scRNA-seq longitudinal data from RA patients

(A) UMAPs of the PBMCs profiled with scRNA-seq from 96 longitudinal samples of 48 RA patients and 8 healthy donors. Cells of each major lineage (T/NK, myeloid, B) were independently clustered and annotated into 29 cell types.

(B) Cell composition across all samples grouped by disease status and time point.

(C) Volcano plot showing the MNRS analysis computed per each cell type and drug combination; the *x* axis represents the odds ratio (OR) (i.e., negative OR representing mitigation of the non-response signature of the first drug by the longitudinal signature of the second drug; positive OR represent exacerbation of the non-response signature of the first drug by the longitudinal effect of the second drug); the *y* axis represents the significance of the MNRS analysis (-log10(p value)).

(D) Scatterplot of the anti-TNF longitudinal effect signature (*x* axis) and the anti-IL-6R non-response signature (*y* axis). Only the immune cell types where the MNRS is significant ($p < 0.05$) are shown. Each point is a gene, colored by significance category (sig.FDR): not significant for either signature (gray), significant for the anti-TNF effect only (light blue), significant for the anti-IL-6R non-response only (green), or significant for both (orange). HLA-DRB1 is labeled.

(E) Gene set enrichment analysis results of the anti-IL-6R non-response (green), anti-TNF longitudinal (red), and JAKi longitudinal (mustard) signatures in $CD14^+$ monocytes. Only significantly enriched pathways (p value < 0.05) in at least two of the signatures are shown; direction of effect is determined by the sign of the NES statistic. See also Figures S6–S9 and Tables S8, S9, and S10.

at baseline showed a persistent downregulation of the HLA-DRB1 gene. Following 3–4 months of anti-TNF therapy, this gene was markedly upregulated across these three classical monocyte subtypes (Figure 3D). These results demonstrate that bulk MNRS predictions are recapitulated at single-cell resolution and allow to identify the specific immune compartments driving the predicted therapeutic complementarity.

Single-cell MNRS analysis of the longitudinal effect of JAKi against the non-response signature to anti-IL-6R showed a highly significant positive association (Figure 3C; Figure S8). Treatment with JAKi was shown to exacerbate the non-response signature to anti-IL-6R identified in $CD14^+$ monocytes (p = 1e−35), cDC2 (p = 1e−13), and $CD14^+$ $DAB2^{Hi}$ monocytes (p = 1e−12) (Figure 3C; Table S9). Comparing the gene ontology (GO) enrichment of the genes associated with anti-IL-6R non-response and the longitudinal effect of the two drugs, we found that, while anti-TNF systematically antagonizes GOs that are differentially enriched in the $CD14^+$ monocytes of anti-IL-6R non-responders, JAKi systematically enhances these same biological processes (Figure 3E; Table S10). Several of the pathways that were oppositely regulated by anti-TNF and JAKi were related with immune response activation. These results indicate that JAKi leads to an exacerbation of the gene expression programs in $CD14^+$ monocytes that are associated with the non-response to anti-IL-6R and suggests that the

combination of both drugs could risk worsening the disease activity of the patients.

Circulating CD14$^+$ monocytes are associated with synovial cell composition in RA and are poised for synovial macrophage differentiation

Single-cell analyses identified that CD14$^+$ monocytes with low expression of HLA-DRB1 are key cell types mediating the complementary effect of anti-TNF and anti-IL-6R therapies. Anti-IL-6R therapy non-responders exhibited significantly lower baseline expression of HLA-DRB1, whereas anti-IL-6R responders displayed expression levels comparable to healthy controls (Figure 4A). In line with this, stratification of CD14$^+$ monocytes based on the presence or absence of HLA-DRB1 expression showed that anti-IL-6R therapy non-responders have a higher proportion of circulating HLA-DRB1-negative CD14$^+$ monocytes compared to both responders and controls (ANOVA p value = 9.67e−4) (Figure 4B). This increased frequency of HLA-DRB1-deficient cells in anti-IL-6R non-responders persisted after 3 months of therapy (ANOVA p = 1.91e−2). Pairwise analyses across the different myeloid cell subsets showed a significant positive correlation of HLA-DRB1 deficiency, suggesting the implication of a common transcriptional program (Figure 4C). Cell-specific coexpression analysis corroborated that HLA-DRB1 is part of a transcriptional module that includes several other members of the HLA class II antigen presentation system, and it is conserved across myeloid cell types (Figure 4C; Figure S10). These findings suggest that the downregulation of HLA-DRB1 and its associated transcriptional program in CD14$^+$ monocytes is a characteristic feature of non-response to anti-IL-6R therapy.

In RA, circulating myeloid cells enter the synovium and differentiate into pathogenic subtypes that contribute to disease progression.[18] To investigate the potential link between circulating HLA-DRB1 levels in CD14$^+$ monocytes and the synovial composition in RA, we analyzed single-cell RNA-seq data of paired PBMCs and synovial biopsies obtained simultaneously from the same RA patients.[19] Synovial cells were annotated based on the Accelerating Medicines Partnership RA synovial atlas,[20] and circulating monocytes were annotated using our PBMC scRNA-Seq dataset as reference (Figure S11). Circulating CD14$^+$ monocytes were subsequently categorized into HLA-DRB^{Hi} and HLA-DRB1-negative subtypes and tested for correlation with the cellular abundance of different cell types in the matched RA synovium. We observed divergent correlation patterns between the two circulating CD14$^+$ subtypes and synovial cell populations (Figure 4D). While circulating CD14$^+$ HLA-DRB1-negative monocytes correlated with the abundance of cells from the myeloid, stromal, endothelial, and NK compartments, the frequency of blood HLA-DRB1^{Hi} monocytes correlated across different T cell subtypes. More specifically, higher circulating CD14$^+$ HLA-DRB1-negative monocytes were associated with higher abundance of different synovial fibroblast subtypes from the lining and sublining (including previously identified NOTCH3+ effector fibroblasts),[21] venous and arterial capillary cells, as well as four NK cell subtypes. In the myeloid compartment, both positive and negative correlations were observed. The former involved anti-inflammatory MERT+ SELENOP+LYVE1+ macrophages while the latter included proinflammatory SPP1+ and IL1B+FCN1+ macrophages. SPP1+ and IL1B+FCN1+ macrophages are MERTK- cells and recent evidence has shown that they are high producers of IL-6 under inflammatory conditions *in vitro*.[18] Our evidence indicates that RA patients with a higher abundance of circulating HLA-DRB1-negative CD14$^+$ monocytes have less IL-6-producing macrophages populating their synovium. Consequently, synovial inflammation in these patients is less dependent on this cytokine; this explains why RA patients with higher levels of HLA-DRB1-negative monocytes in blood respond poorly to the blocking of IL-6 signaling pathway.

To further understand the relationship between HLA-DRB1 expression in circulating CD14$^+$ monocytes and macrophage differentiation, we performed an *in vitro* differentiation analysis. CD14$^+$CD16$^-$ monocytes were isolated and sorted into HLA-DRB1-negative and HLA-DRB1^{Hi} subsets (Figure 4E). After 6 days of *in vitro* differentiation with M-CSF, the transcriptome of the two resulting macrophage populations were analyzed using scRNA-Seq. Gene expression analysis showed a marked transcriptional difference between the two macrophage populations (n = 1,648 genes, $p_{adjusted}$ < 0.01 and absolute fold change > 0.2) (Table S11). HLA-DRB^{Hi} derived macrophages showed a higher percentage of upregulated genes (n = 1,073, 65.1%) compared to HLA-DRB1-negative derived macrophages (n = 575, 34.9%). Gene set analyses in HLA-DRB^{Hi} macrophages revealed an increased activation of biological processes associated with viral life cycle and antigen presentation via MHC class II (Table S12). Conversely, the genes upregulated in HLA-DRB1-negative derived macrophages were predominantly enriched in non-immune processes like organic compound transport into the cell. This result supports the existence of a marked regulatory program associated with HLA-DRB1 expression in CD14$^+$ circulating monocytes. We next evaluated if this regulatory program predisposes to the differentiation toward a specific synovial macrophage population. Using the marker genes for each synovial macrophage subtype[20] (AUC > 0.75, FDR < 0.01), we found a significant positive enrichment for SPP1+ ($p_{adjusted}$ = 0.0018; Figure 4F) and STAT1+CXCL10+ ($p_{adjusted}$ = 0.024) macrophage markers with the HLA-DRB1^{Hi} signature. This result suggests an increased predisposition of HLA-DRB^{Hi} to differentiate into MERTK- pro-inflammatory synovial macrophages that produce high levels of IL-6. This is also in line with our paired blood-synovium analysis where patients with more HLA-DRB1-negative monocytes were found to have less SPP1+ macrophages in their synovial membrane. These results further support the presence of a gene regulatory program associated with HLA-DRB1 expression that poises circulating monocytes toward specific pathogenic macrophages in the synovium and that influences response to therapy.

Anti-TNF elicits a profound activation program in monocytes *in vitro*

To investigate the mechanisms underlying the longitudinal effect of anti-TNF and its direct impact on the circulating immune populations, we performed an *in vitro* analysis of PBMCs treated with anti-TNF. PBMCs from six RA patients (Table S13) were treated

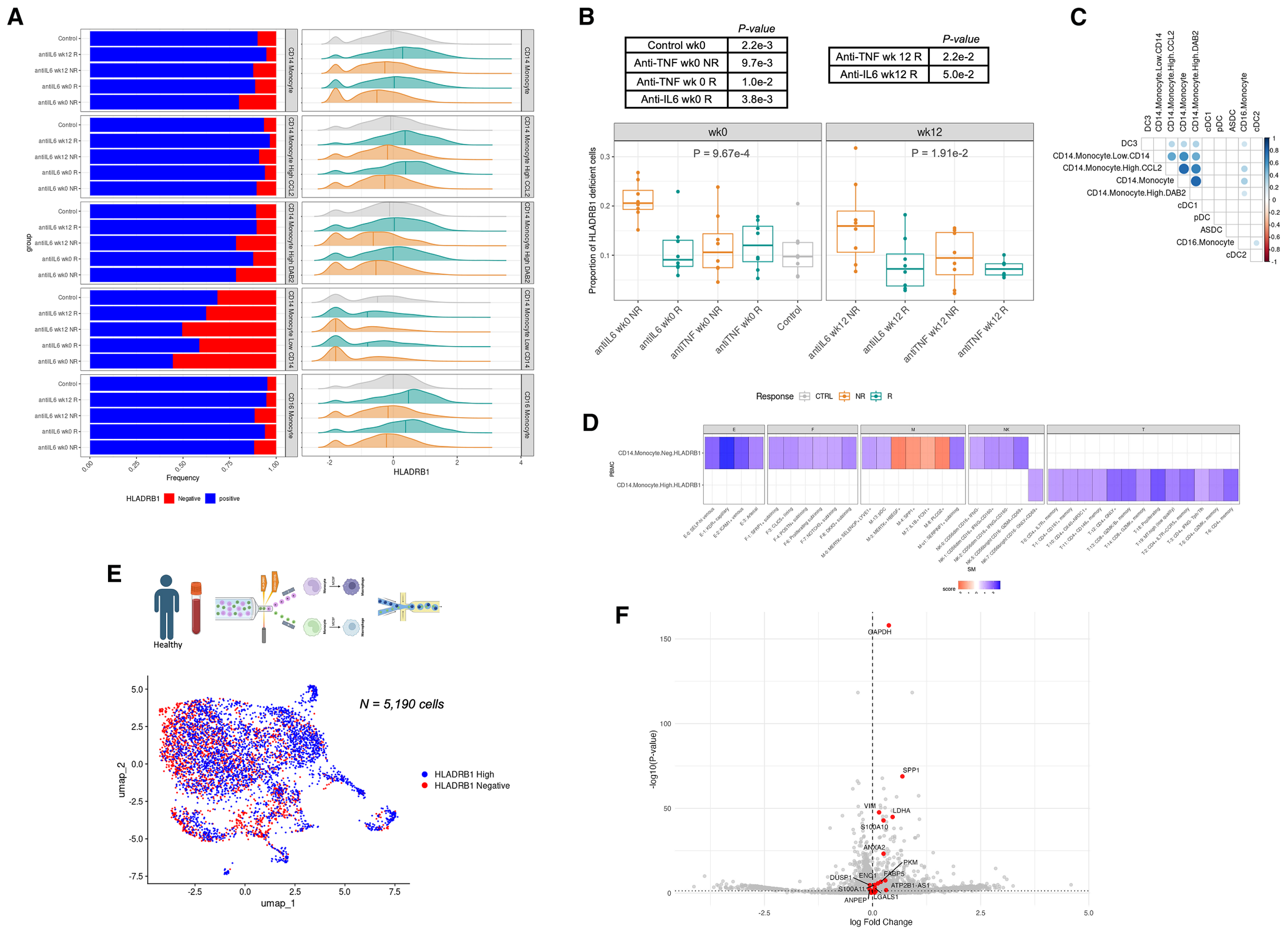

Figure 4. Distribution of HLA-DRB1 expressing myeloid cells in blood and synovial tissue

(A) Left: proportion of HLA-DRB1 expressing (blue) and non-expressing (red) cells across different myeloid cells in RA and healthy donors. Right: density plots of HLA-DsRB1 expression in myeloid cells. Responders (green), non-responders (orange), and healthy donors (gray).

(B) Distribution of HLA-DRB1 negative monocytes (CD14^{+}, CD14^{+} CCL2^{Hi}, CD14^{+} DAB2^{Hi}, and CD14^{+} CD14^{Lo}) per cohort and time point. The *p* values correspond to the ANOVA tests at each time point, with anti-IL-6R non-responders as the reference group.

(C) Heatmap of the pairwise correlations of HLA-DRB1-negative cells across all myeloid types. Only significant results (*p* value < 0.05) are shown with color gradient based on Pearson's r.

(D) Pairwise correlations between the frequency of circulating CD14^{+} monocytes stratified by HLA-DRB1 expression and the frequency of cell types in the synovium in RA patients. Only significant correlations are shown (blue: positive correlation, red: negative correlation; score: –log10(*p* value) multiplied by the correlation sign).

(E) UMAP of *in vitro* differentiated CD14^{+} macrophages originating from HLA-DRB1-stratified monocytes.

(F) Volcano plot of differentially expressed genes between HLA-DRB1^{Hi} and HLA-DRB1-negative derived macrophages. Marker genes of SPP1+ synovial macrophages (AUC > 0.75) are highlighted in red. For (A)–(C), data derived from the longitudinal scRNA-seq cohort (48 RA patients, 8 healthy donors); synovial frequencies in (D) derived from the external paired PBMC-synovium dataset (17 RA patients). For (E) and (F), *in vitro* differentiated macrophages were generated from HLA-DRB1-sorted monocytes of one RA donor. See also Figures S10 and S11, and Tables S11 and S12.

with anti-TNF for 24 h. Cells were pre-incubated with TNF or left untreated, and both pre-stimulated and unstimulated cells were incubated with anti-TNF or an isotype control (ISO), resulting in four experimental conditions that were analyzed using scRNA-seq. After quality control, the single-cell transcriptomes of 34,984 *in vitro* treated mononuclear cells were available for analysis (Figure 5A).

We found a high number of differentially expressed genes after treatment with anti-TNF in myeloid cells (Figure 5B). A substantial number of these differential genes were shared between TNF-pre-stimulated and unstimulated cells (73.7% commonly upregulated and 73.2% commonly downregulated), suggesting a dominant transcriptional program linked to anti-TNF response (Figure 5C). In the PBMC samples treated with anti-TNF, a clearly differentiated group of monocytes emerged (M1 cluster). This cluster was much less abundant in non-treated PBMCs (M1 frequency: anti-TNF: 50.2%; TNF + anti-TNF: 36.8%, ISO: 7.9%; ISO+TNF: 4.9%; Figures 5D and 5E). To characterize this drug-induced cell cluster, we compared it against the undifferentiated (M0) monocyte cluster. A total

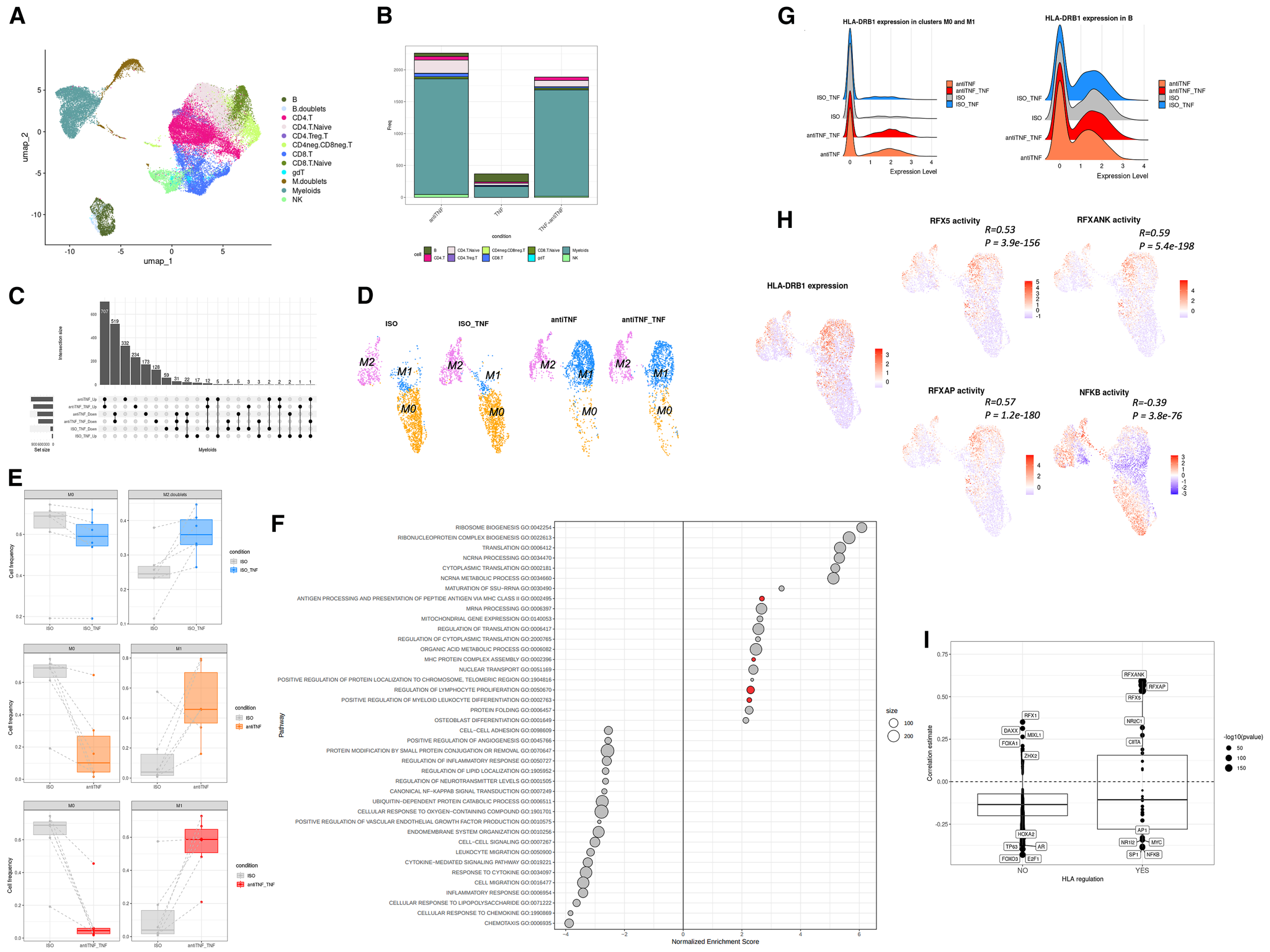

Figure 5. *In vitro* effects of anti-TNF on PBMCs from RA donors

(A) UMAP of scRNA-Seq data from 34,984 PBMCs cultured *in vitro* and treated with anti-TNF and/or TNF pre-stimulation.

(B) Number of differentially expressed genes per immune compartment and condition.

(C) Upset plot representing intersections of significant differentially up- and downregulated genes across all tested conditions in myeloid cells.

(D) UMAP of myeloid cells colored by unsupervised Louvain clusters annotated as M0, M1, and M2.doublets.

(E) Boxplots of M0 and M1 monocyte frequencies per condition. ISO, untreated cells (isotype control); ISO_TNF, cells preincubated with TNF and untreated; anti-TNF, cells treated with anti-TNF for 24 h; anti-TNF_TNF, cells pre-incubated with TNF and treated with anti-TNF for 24 h.

(F) Gene set enrichment analysis (GSEA) of differentially expressed genes between M0 and M1 clusters. HLA-DRB1 is contributing to the enrichment of pathways colored in red.

(G) HLA-DRB1 expression in M0 and M1 clusters (left) and B cells (right), across conditions.

(H) UMAPs of myeloid cells indicating levels of expression of HLA-DRB1, and the estimated activities of transcription factors RFX5, RFXANK, RFX, and NFKB activities. Pearson correlation values (r) and p values between each TF and HLA-DRB1 are included.

(I) Boxplots of correlation estimates between HLA-DRB1 expression and TF activity across all tested TFs. YES: TFs previously associated with HLA-DRB1 expression regulation according to CollecTRI database. NO: TFs with no previous evidence of HLA-DRB1 regulation. Only correlations with a p value < 0.05 are annotated. Biological replicates are individual donors: six RA donors, each profiled under four conditions. See also Tables S13, S14, S15, S16, S17, and S18.

of 2,384 genes were found to be differentially expressed between the two clusters (p value$_{adjusted}$ < 0.05; Table S14). Among the differentially expressed genes, we found 15 of the 32 genes of the previously identified HLA-DRB1 transcriptional module. In line with this, functional analysis of the upregulated genes from the anti-TNF induced M1 cluster showed a significant enrichment in pathways associated with antigen processing and presentation via MHC class II (Figure 5F; Table S15). Among the genes most significantly upregulated by anti-TNF therapy was HLA-DRB1 (anti-TNF vs. ISO: p value$_{adjusted}$ = 2.7e−42; anti-TNF+TNF vs. ISO: p value$_{adjusted}$ = 1.63e−47) (Table S16). Together, these results support that the increase in circulating HLA-DRB1 positive cells observed in patients treated with anti-TNF could be due to a direct effect of the drug in circulating or developing bone marrow monocytes.

This marked differentiation effect induced by the anti-TNF was not seen in B, T, or NK cells. B cells also constitutively express HLA-DR genes to present antigens to CD4^{+} T cells. However,

we found no significant change in HLA-DRB1 expression in B cells following *in vitro* treatment with anti-TNF (Figure 5G). This is in line with our findings in patients, where upregulation of HLA-DRB1 is specifically detected in monocytes and not in B cells. These results suggest the presence of a monocyte-specific mechanism associated with stimulation by anti-TNF therapy.

TNF is initially expressed as a membrane protein (mTNF) and inflammatory activation leads to its shedding to soluble TNF (sTNF) by ADAM17 metalloproteinase. Previous studies have suggested the presence of a reverse signaling mechanism by mTNF.[22] We hypothesized that the major transcriptional changes observed in monocytes is due to the signaling of mTNF upon binding to the anti-TNF molecule. To uncover the regulatory network driving this reverse signaling, we inferred the activity of 772 transcription factors (TFs) at the single-cell level.[23] In the M1 monocyte population, we found a highly significant enrichment of the activity of three TFs belonging to the regulatory factor X (RFX) complex: RFX5, RFXAP, and RFXANK (Table S17). As expected, these TFs were highly correlated with the expression of *HLA-DRB1* (Figures 5H and 5I, $p < 1e{-}150$, $r > 0.5$). Analysis of the longitudinal data from patients treated with anti-TNF further supported these findings, showing that anti-TNF therapy also significantly upregulates the activity of RFX5, RFXAP, and RFXANK in $CD14^+$ monocytes *in vivo* ($p_{RFX5} = 0.002$; $p_{RFXAP} = 0.002$; $p_{RFXANK} = 0.002$) (Table S18). In contrast, anti-IL6R therapy only induced a moderate upregulation of the RFX transcriptional complex, and treatment with JAK inhibitors did not result in significant changes. Together these results suggest that anti-TNF therapy has a strong direct regulatory effect in $CD14^+$ monocytes, inducing an HLA-DRB1 module upregulation via mTNF reverse signaling.

Combined anti-TNF and anti-IL-6R therapy enhances efficacy in a murine model of RA

To preclinically validate the anti-TNF and anti-IL-6R combinatorial therapy for RA, we implemented a collagen-induced arthritis mouse model on the arthritic-prone DBA1/Jr mouse strain (Figure 6A). Immunized mice presenting clinical signs of arthritis and paw swelling were randomized into isotype-control, anti-TNF (etanercept), anti-mIL-6R and anti-TNF (etanercept) + anti-mIL-6R groups, and received six doses of each therapy over the course of 2 weeks.

As expected, isotype control antibodies were unable to control disease progression in arthritic mice. Both paw swelling and clinical scores increased over the course of treatment, reaching the highest scores at the end of the follow-up (Figure 6B). Surprisingly, anti-mIL-6R therapy afforded limited protection against arthritis. Upon treatment onset, paw thickness in anti-mIL-6R-treated mice progressed to a similar extent as in isotype control-treated mice, only showing a small reduction in paw swelling at later stages of treatment (Figure 6B). Furthermore, anti-mIL-6R treatment did not improve the progression of clinical signs of disease as compared to isotype control-treated animals (Figure 6B). In contrast, treatment with etanercept stopped the progression of arthritis on DBA1/Jr mice shortly after the onset of therapeutic intervention. While etanercept was unable to reverse the arthritic symptomatology on DBA1/Jr mice, paw swelling and clinical score readouts did not increase throughout the etanercept monotherapy, resulting in a significantly improved clinical score compared to the isotype control-treated group (Figure 6B). In contrast, combinatorial therapy with etanercept and anti-mIL-6R allowed a rapid decrease of paw thickness and clinical scores of arthritic DBA1/Jr mice (Figure 6B). Etanercept and anti-mIL-6R combination significantly ameliorated both clinical readouts compared to isotype control and anti-mIL6R therapeutic groups. The rapid beneficial effect of the combinatorial therapy on the course of arthritis even allowed an improvement of the paw thickness increase compared to etanercept monotherapy at early time points after treatment onset (Figure 6B). Thus, whereas etanercept monotherapy only was able to stop disease progression and anti-mIL-6R monotherapy elicited a limited effect on paw swelling of arthritic DBA1/Jr mice, the combination of both monotherapies enabled a reduction of paw swelling below the therapeutic threshold, with a similar reduction at the clinical score level.

Collectively, these preclinical results show that the combination of anti-TNF and anti-IL-6R therapies can suppress and reverse experimental arthritis. Importantly, these *in vivo* results validate our transcriptomic and *in vitro* findings, reflecting a positive synergism between anti-TNF and anti-IL-6R therapies in RA.

DISCUSSION

Combination therapies are emerging as a promising strategy to increase the level of therapeutic response in IMIDs and achieve sustained remission in a large percentage of patients. However, the number of potential combinations is high and increasing with every new drug approval. Consequently, there is a major need to identify which are more likely to be synergistic and prioritize them for clinical testing. Here, we developed a systems biology approach, the MNRS, for this objective. The MNRS leverages longitudinal transcriptional data from patients treated with monotherapy to identify which drug optimally antagonizes the signature of non-response to another drug.

The MNRS approach extends the SRP[13] to the context of systemic inflammatory diseases and to the problem of drug combinations. In oncology, the availability of a precise pathological tissue—the tumor—that can also be easily expanded *in vitro*, allows the application of the SRP to high-throughput *in vitro* screens to many candidate compounds. This large-scale testing potential has been recently applied to identify combination therapies in cancer.[24] In chronic inflammatory diseases like IMIDs, however, large-scale drug screenings in the target tissue are not possible since the inflamed organs cannot be easily reproduced *in vitro*. The approach we describe here, the MNRS, leverages the longitudinal (*in vivo*) responses measured in the blood of IMID patients to capture the systemic effect to targeted drugs. Using this approach, we previously demonstrated that it allows an unbiased evaluation of drugs for the treatment of inflammatory diseases with a systemic component like severe COVID-19.[14] Here, we extend this approach to the problem of drug combinations for IMIDs. Among the 55 candidate drug combinations tested using the MNRS approach, we have found several pairs of targeted drugs that show significant complementary signatures and could be eligible for clinical testing. Among these candidate combinations, we have found support for the

CellPress
OPEN ACCESS

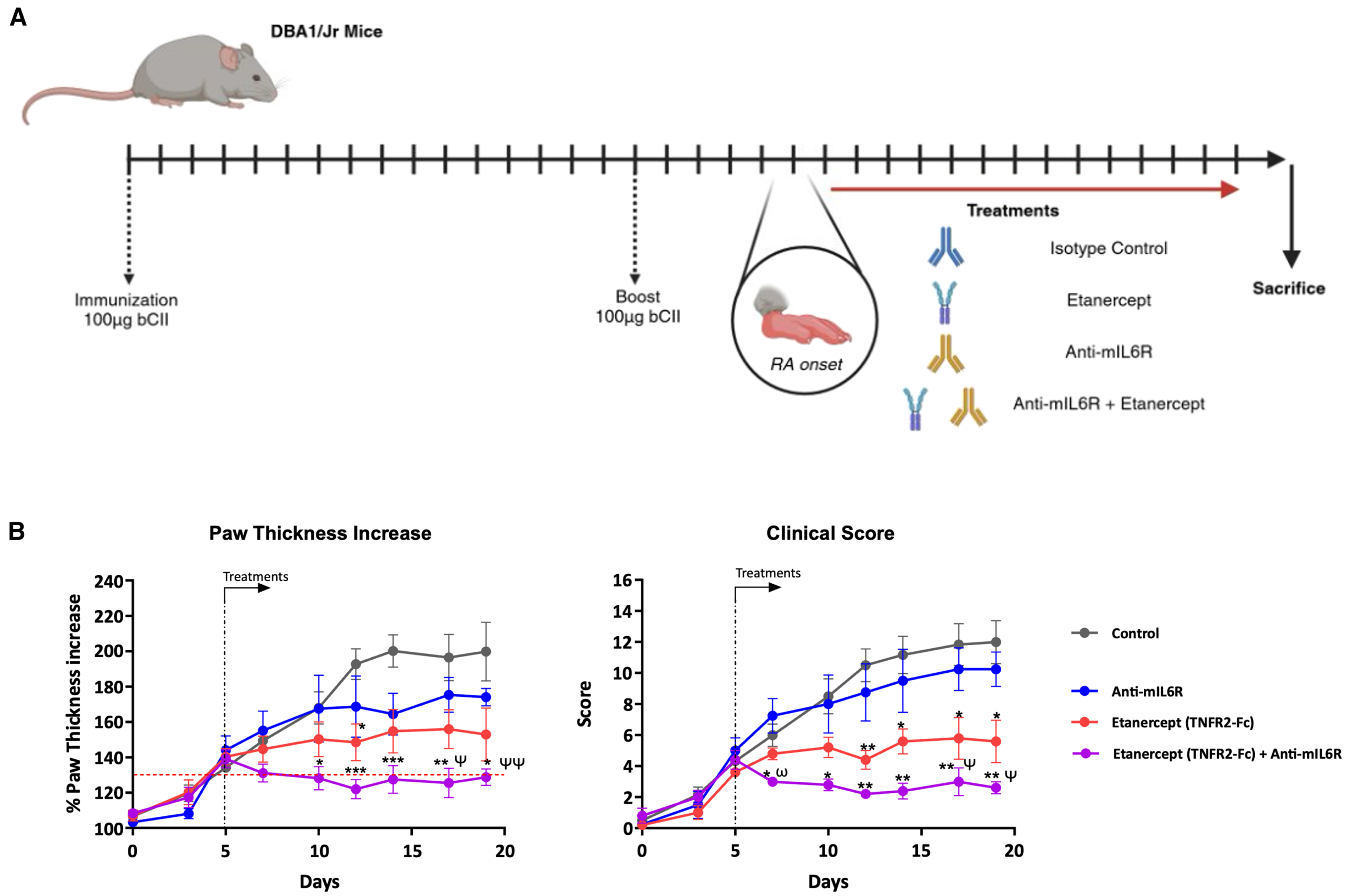

Figure 6. Effects of anti-TNF and anti-IL-6R in a collagen-induced arthritis mouse model

(A) DBA1/Jr mice immunized with 100 µg of bovine collagen II in CFA and boosted 17 days after the initial immunization have been treated with etanercept, anti-mIL-6R, etanercept + anti-mIL-6R or isotype control antibodies upon development of arthritis signs. Paw swelling and clinical score of animals have been monitored until sacrifice 48 h after the last therapeutic administration.

(B) Evaluation of therapeutic effects etanercept and anti-mIL-6R monotherapies, etanercept + anti-mIL-6R combination or isotype control antibodies on paw thickness increase (left) or clinical scores (right) of arthritic DBA1/Jr mice. Statistical analyses: two-way ANOVA followed by Tukey's multiple comparisons test performed. In *, comparisons against isotype control. *p value < 0.05; **p value < 0.01; ***p value < 0.001. In ψ, comparisons against anti-mIL-6R. $^{\Psi}p$ value < 0.05; $^{\Psi\Psi}p$ value < 0.01. In ω, comparisons with etanercept. $^{\omega}p$ value < 0.05. Data are shown as mean ± SEM. n = 9 (isotype control) and n = 5 per treatment group (anti-mIL-6R, etanercept, and etanercept + anti-mIL-6R).

combination between anti-IL-12/23 and anti-TNF in UC. Very recently, the VEGA study, a randomized double-blind clinical trial in ulcerative colitis, has shown preliminary evidence that the combination of anti-TNF and anti-IL-23 therapies might be more effective than therapy with either drug alone.[25] Our results are in line with this finding and also support the use of a broader spectrum drug to complement anti-TNF therapy in UC. Additionally, another case series has provided evidence in favor of the combination of anti-IL-12/23 and JAKi combination to treat CD.[26] Results obtained by applying the MNRS framework in longitudinal patient data also support the efficacy of this combination and extend its beneficial effect to UC. Together with the increasing availability of longitudinal transcriptomic data on response to new drugs, our approach could be extended to test additional therapeutic combinations in IMIDs.

Among the best scoring combinations in our approach, anti-TNF and anti-IL-6R therapy in RA showed a strong and consistent signal. We subsequently followed up this drug combination to investigate its potential mechanisms of action and to provide proof-of-concept that MNRS prioritizes drug combinations that are beneficial for IMIDs. Our *in vivo* and *in vitro* results show that anti-TNF therapy has a strong direct effect on $CD14^{+}$ monocytes and that these changes complement the non-response signature of anti-IL-6R therapy. These changes are characterized by the increase of HLA-DRB1 expression. Paired blood and synovial cell analyses showed a negative correlation between circulating HLA-DRB1 negative monocytes and the abundance of SPP1+ and IL1B+FC1N+ proinflammatory macrophages in the synovium. Both cell types are MERTK- proinflammatory macrophages, which have been previously associated with a high production of IL-6.[18] Consequently, a lower presence of these macrophage subtypes in the synovium of anti-IL-6R non-responders could be shaping a joint inflammation that is less dependent on IL-6 signaling and, therefore, would explain the ineffectiveness of anti-IL-6R therapy observed in these patients. Our *in vitro* differentiation analysis further supported this

model, showing that HLA-DRB1^{Hi} monocytes are poised for differentiation into MERTK- proinflammatory macrophages SPP1+ and STAT1+CXCL10+. These results demonstrate that expression of HLA-DRB1 in circulating monocytes conditions their differentiation into key macrophage subsets in the synovium of RA patients and ultimately conditions the patient's likelihood of response to therapy.

Our findings using the MNRS also caution against the use of certain drug combinations to treat IMIDs. Such is the case of anti-IL-6R and JAKi for the treatment of RA. When applying the MNRS approach at the single-cell level, the longitudinal effect of JAKi was found to exhibit a strong positive correlation with the signature of non-response to anti-IL-6R therapy in CD14^{+} monocytes. This overlap suggests a potential risk of promoting regulatory changes in the myeloid compartment that could exacerbate the disease mechanisms associated with non-response to IL-6R in RA. Similarly, the MNRS analysis in blood, showed a high positive correlation between the longitudinal effect of anti-IL-6R and the response to anti-BLyS in SLE. In fact, none of the five tested drugs for combination with belimumab for SLE showed a significant antagonism. Our approach therefore does not support combination therapy for SLE, at least for the combinations tested in this study. The recent introduction of new therapies for SLE[27] and, importantly, the generation of longitudinal molecular data will allow the evaluation using the MNRS approach of new combinations that could be highly beneficial for SLE.

Our study supports the existence of a profound reverse signaling system through the membrane TNF (mTNF) in monocytes. Previous studies have shown that mTNF, when bound to its cognate receptors TNFRSF1A and TNFRSF1B expressed on other cell types, can elicit diverse cellular effects in trans.[22] Our results extend this understanding, showing that mTNF also exerts a powerful reverse signaling mechanism when bound to its ligand. We have found that this reverse signaling elicited by the anti-TNF drug activates a large gene expression program that involves upregulation of HLA-DRB1 and its associated module in monocytes. In line with this, recent evidence in transgenic mouse models[28] supports reverse signaling through mTNF as a powerful *in vivo* anti-inflammatory mechanism that is mediated by the polarization of macrophages. This mechanism not only could play a pivotal role in the efficacy of the combination of anti-TNF and anti-IL-6R in RA but also could help us understand the variability of response to the drug in monotherapy. Predicting the response to anti-TNF therapy has been an elusive objective in translational research in IMIDs. Our discovery of a strong drug-induced effect in monocytes could be the basis for future precision medicine studies aimed at finding better ways to stratify patients to anti-TNF therapy.

Despite significant advances in targeted therapies, achieving sustained remission remains challenging for many IMID patients, with response rates varying widely across diseases and therapies. In RA, for example, 40% of patients fail to respond to monotherapies and only 10% reach remission,[29] highlighting the need for novel therapeutic approaches. Driven by the success in cancer, combination therapies are increasingly being considered as a powerful therapeutic strategy to increase remission rates in IMIDs. Our study addresses this unmet need by providing an approach, the MNRS, that leverages longitudinal transcriptomic patient data to identify the optimal combinations. The increasing availability of longitudinal datasets of response to targeted therapies, including those coming from clinical trials, could help expand this approach to other IMIDs and indications, and change the drug combination paradigm from a trial-and-error process to a data-driven approach.

Limitations of the study

This study has several limitations that point to opportunities for future work. The MNRS relies on peripheral blood transcriptomic responses, which may not fully capture tissue-resident immune programs driving therapeutic response. Integrating matched tissue-blood datasets and perturbation assays will be essential to refine mechanistic inference. The combinations evaluated are constrained by the therapies for which longitudinal data are currently available, and the MNRS cannot discriminate with certainty whether predicted synergies require simultaneous administration or could be achieved through sequential treatment. While we provide mechanistic validation for one high-priority combination, further experimental and clinical assessment of additional MNRS-predicted synergies will be needed to generalize these findings across IMIDs. Moreover, incorporating pharmacologic, safety, and real-world evidence will be important to translate MNRS predictions into clinically actionable strategies. As larger longitudinal and single-cell datasets become available, MNRS has the potential to evolve into a scalable, data-driven framework for guiding precision combinatorial therapy development in immune-mediated diseases.

RESOURCE AVAILABILITY

Lead contact

Requests for further information and resources should be directed to and will be fulfilled by the lead contact, Antonio Julià (tonijuliacano@gmail.com).

Materials availability

This study did not generate new unique reagents.

Data and code availability

- Single-cell and bulk RNA-seq data reported in this paper have been deposited at the European Genome-Phenome Archive (EGA) and are available under controlled access through the the Data Access Committee (EGAC50000000566), EGA: EGAD50000001964, EGAD50000002832, EGAD50000002833, and EGAD50000002834. Processed RNA-seq and scRNA-seq datasets and metadata have been deposited at Zenodo: https://doi.org/10.5281/zenodo.14999133. Accession numbers and the DOI are also listed in the key resources table.
- All original code has been deposited at Zenodo: https://doi.org/10.5281/zenodo.21476753. The DOI is listed in the key resources table.
- Any additional information required to reanalyze the data reported in this paper is available from the lead contact upon request.

CONSORTIA

The members of the IMID-Consortium are María Ángeles Aguirre Zamorano, Mercedes Alperi-López, José Luis Andréu, Manuel Barreiro, Isabel Belinchón, Diego Benavent, Francisco Blanco, Susana Blanco, Ricardo Blanco, Natalia Borruel, Luis Bujanda, José Luis Cabriada, Jaime Calvo-Alén, Juan D. Cañete, José Manuel Carrascosa, Patricia Carreira, Santos Castañeda, Hèctor Corominas, Josefina Cortés, Pablo de la Cueva, Esteban Daudén, César Díaz,

CellPress
OPEN ACCESS

Eugeni Domènech, Elvira Díez, Alba Erra, Maria Esteve Comas, Benjamín Fernández, Antonio Fernández-Nebro, Eduardo Fonseca, Mercedes Freire, Esther García-Planella, Vicenç García-Patos, Rosario García de Vicuña, María Jesús García-Villanueva, María Luz García Vivar, Fernando Gomollón García, Carlos González, María José González, Isidoro González, Jordi Gratacós, Jordi Guardiola, Iván Guerra, Ana Gutiérrez, Enrique Herrera, Susana Holgado, Iago Rodríguez-Lago, Eva Iglesias, Beatriz Joven, Ana Laiz, Laura López, Antonio López-Sanromán, José Luis López Estebaranz, Marta Loredo Martínez, Berta Magallares, José Luis Marenco de la Fuente, Ignacio Marín-Jiménez, Carlos Marras, Andrea Pluma, María Dolores Martín Arranz, Julia Martínez-Barrio, Víctor Martínez-Taboada, Lourdes Mateo, Juan Luis Mendoza, Francisco Mesonero, Joan Miquel Nolla, Jordi Monfort, Carlos Montilla, Estefania Moreno, David Moreno Ramírez, Santiago Muñoz, Fernando Muñoz, Antonio Naranjo, Natividad Oreiro-Villar, Pilar Nos Mateu, Jaime Notario, Ana Ortiz, Pedro Zarco, José María Pego-Reigosa, Javier P. Gisbert, José Javier Pérez-Venegas, José Antonio Pinto-Tasende, Lluís Puig, Ramon Pujol, Rubén Queiro, Delia Reina, Elena Ricart, Pilar del Río Martínez, Raquel Rivera, Anne Riveros, Esther Rodríguez, Daniel Roig, Mónica Roncero Riesco, Iñigo Jesús Rúa-Figueroa, Tarek Carlos Salman-Monte, Georgina Salvador, Simón Sánchez-Fernández, José Luis Sánchez Carazo, Raimon Sanmartí, Mónica Sierra-Ausín, Carles Tomàs, Jesús Tornero, Juan Carlos Torre-Alonso, Paloma Vela, and Maribel Vera.

ACKNOWLEDGMENTS

Samples used in this study were provided by the IMID-Biobank of the Hospital Universitari Vall d'Hebron Biobank (biobank ID B. 0000618, PT20/00107), integrated in the Instituto de Salud Carlos III Platform Biobanks and Biomodels, and they were processed following standard operating procedures with the appropriate approval of the Ethics and Scientific Committees. S. Marsal is the coordinator of the IMID Consortium. We would like to acknowledge the support of Isbaal Ramos, Laura Sesma, and Mariano Echávarri from the European Projects Department at Zabala Innovation. Schematic figures have been elaborated using BioRender. This project has received funding from the European Union's H2020 research and innovation program under grant agreement no. 848028 (DoCTIS; Decision on Optimal Combinatorial Therapies in IMIDs Using Systems Approaches). S.H.M.-M., Y.G., and D.Á.-E. were supported by IMIDomics. L.J.-G. has held an FPU PhD fellowship (FPU19/04886) from the Spanish Ministry of Universities.

AUTHOR CONTRIBUTIONS

Conceptualization, S.H.M.-M., Y.G., S. Marsal, and A.J.; methodology, all authors; computational analysis, S.H.M.-M., Y.G., A.A., and A.J.; data generation, H.H., L.J.-G., M.R., R.M.M., and I.R.-N.; animal model analysis, E.A., P. Serra, and P. Santamaria; *in vitro* analyses, D.Á.-E. and R.T.; writing – original draft, S.H.M.-M., Y.G., E.A., P. Santamaria, and A.J.; writing – review and editing, S.H.M.-M., Y.G., E.A., M.A.L.L., S. Madonna, L.J.-G., A.A., D.Á.-E., G.G., B.S., E.H.S.C., R.M.M., I.R.-N., H.H., P. Santamaria, S. Marsal, and A.J.; supervision, A.J. and S. Marsal.

DECLARATION OF INTERESTS

H.H. is co-founder and chief scientific officer of Omniscope, scientific advisory board member at Nanostring and Mirxes, consultant for Moderna and Singularity, and has received honoraria from Genentech. P. Santamaria is founder, scientific officer, and stockholder of Parvus Therapeutics. He receives funding from the company. He also has a consulting agreement with Sanofi. A.J. has served as chief data scientist of IMIDomics. R.M.M. is a co-founder of IMIDomics. S. Marsal is co-founder and Chief Medical officer of IMIDomics.

STAR★METHODS

Detailed methods are provided in the online version of this paper and include the following:

- KEY RESOURCES TABLE
- EXPERIMENTAL MODEL AND STUDY PARTICIPANT DETAILS
  - Patients and clinical sample collection
  - Collagen-induced arthritis mouse model
- METHOD DETAILS
  - Bulk RNA-seq
  - Whole blood bulk transcriptomic data
  - Single-cell RNA-seq
  - Single-cell RNA-seq analysis of monocyte-derived macrophages *in vitro*
  - *In vitro* characterization of anti-TNF effect in PBMCs
  - CIA mouse model
  - Antibody production
- QUANTIFICATION AND STATISTICAL ANALYSIS
  - Patient selection and covariate balancing
  - Gene expression signatures
  - Analysis of drug combinations using MNRS
  - Biological characterization of the drug non-response and drug-effect signatures
  - Single-cell RNA-seq of the DoCTIS cohort
  - Genotyping for scRNA-Seq demultiplexing
  - Single-cell RNA-seq of *in vitro* response to anti-TNF therapy
  - Single-cell RNA-Seq analysis of PBMCs and synovial biopsies from RA patients
  - Single-cell RNA-Seq analysis of *in vitro* differentiated HLA-DRB1-negative/High monocytes
  - TF activity

SUPPLEMENTAL INFORMATION

Supplemental information can be found online at https://doi.org/10.1016/j.xcrm.2026.103026.

Received: June 6, 2025
Revised: June 30, 2026
Accepted: August 12, 2026
Published: September 11, 2026

REFERENCES

1. McInnes, I.B., and Gravallese, E.M. (2021). Immune-mediated inflammatory disease therapeutics: past, present and future. Nat. Rev. Immunol. *21*, 680–686. https://doi.org/10.1038/s41577-021-00603-1.

2. Marsal, J., Barreiro-de Acosta, M., Blumenstein, I., Cappello, M., Bazin, T., and Sebastian, S. (2022). Management of Non-response and Loss of Response to Anti-tumor Necrosis Factor Therapy in Inflammatory Bowel Disease. Front. Med. *9*. https://doi.org/10.3389/fmed.2022.897936.

3. Maini, R.N., Breedveld, F.C., Kalden, J.R., Smolen, J.S., Furst, D., Weisman, M.H., St Clair, E.W., Keenan, G.F., van der Heijde, D., Marsters, P.A., et al. (2004). Sustained improvement over two years in physical function, structural damage, and signs and symptoms among patients with rheumatoid arthritis treated with infliximab and methotrexate. Arthritis Rheum. *50*, 1051–1065. https://doi.org/10.1002/art.20159.

4. Furst, D.E., Pangan, A.L., Harrold, L.R., Chang, H., Reed, G., Kremer, J.M., and Greenberg, J.D. (2011). Greater Likelihood of Remission in Rheumatoid Arthritis Patients Treated Earlier in Disease Course: Results from the CORRONA Registry. Arthritis Care Res. *63*, 856–864. https://doi.org/10.1002/acr.20452.

5. Hasegawa, Y., Arinuma, Y., Asakura, H., Shindo, R., Ino, K., Kanayama, Y., Tanaka, T., Matsueda, Y., Wada, T., Oku, K., and Yamaoka, K. (2024). Real-world efficacy of belimumab in achieving remission or low-disease activity in systemic lupus erythematosus: A retrospective study. Mod. Rheumatol. *34*, 732–740. https://doi.org/10.1093/mr/road078.

6. Raine, T., and Danese, S. (2022). Breaking Through the Therapeutic Ceiling: What Will It Take? Gastroenterology *162*, 1507–1511. https://doi.org/10.1053/j.gastro.2021.09.078.

7. Solitano, V., Ma, C., Hanžel, J., Panaccione, R., Feagan, B.G., and Jairath, V. (2023). Advanced Combination Treatment With Biologic Agents and Novel Small Molecule Drugs for Inflammatory Bowel Disease. Gastroenterol. Hepatol. *19*, 251–263.

8. Subramanian, A., Narayan, R., Corsello, S.M., Peck, D.D., Natoli, T.E., Lu, X., Gould, J., Davis, J.F., Tubelli, A.A., Asiedu, J.K., et al. (2017). A Next Generation Connectivity Map: L1000 Platform and the First 1,000,000 Profiles. Cell *171*, 1437–1452.e17. https://doi.org/10.1016/j.cell.2017.10.049.

9. Koleti, A., Terryn, R., Stathias, V., Chung, C., Cooper, D.J., Turner, J.P., Vidović, D., Forlin, M., Kelley, T.T., D'Urso, A., et al. (2018). Data Portal for the Library of Integrated Network-based Cellular Signatures (LINCS) program: integrated access to diverse large-scale cellular perturbation response data. Nucleic Acids Res. *46*, D558–D566. https://doi.org/10.1093/nar/gkx1063.

10. Sirota, M., Dudley, J.T., Kim, J., Chiang, A.P., Morgan, A.A., Sweet-Cordero, A., Sage, J., and Butte, A.J. (2011). Discovery and preclinical validation of drug indications using compendia of public gene expression data. Sci. Transl. Med. *3*, 96ra77. https://doi.org/10.1126/scitranslmed.3001318.

11. Yu, K., Basu, A., Yau, C., Wolf, D.M., Goodarzi, H., Bandyopadhyay, S., Korkola, J.E., Hirst, G.L., Asare, S., DeMichele, A., et al. (2023). Computational drug repositioning for the identification of new agents to sensitize drug-resistant breast tumors across treatments and receptor subtypes. Front. Oncol. *13*, 1192208. https://doi.org/10.3389/fonc.2023.1192208.

12. Wang, X., Yang, L., Yu, C., Ling, X., Guo, C., Chen, R., Li, D., and Liu, Z. (2023). An integrated computational strategy to predict personalized cancer drug combinations by reversing drug resistance signatures. Comput. Biol. Med. *163*, 107230. https://doi.org/10.1016/j.compbiomed.2023.107230.

13. Pushpakom, S., Iorio, F., Eyers, P.A., Escott, K.J., Hopper, S., Wells, A., Doig, A., Guilliams, T., Latimer, J., McNamee, C., et al. (2019). Drug repurposing: progress, challenges and recommendations. Nat. Rev. Drug Discov. *18*, 41–58. https://doi.org/10.1038/nrd.2018.168.

14. Julià, A., Bonafonte-Pardàs, I., Gómez, A., López-Lasanta, M., López-Corbeto, M., Martínez-Mateu, S.H., Lladós, J., Rodríguez-Nunez, I., Myers, R.M., and Marsal, S. (2021). Targeting of the CD80/86 proinflammatory axis as a therapeutic strategy to prevent severe COVID-19. Sci. Rep. *11*, 11462. https://doi.org/10.1038/s41598-021-90797-0.

15. Balevic, S.J., Benjamin, D.K., Jr., Powderly, W.G., Smith, P.B., Gonzalez, D., McCarthy, M.W., Shaw, L.K., Lindsell, C.J., Bozzette, S., Williams, D., et al. (2024). Abatacept Pharmacokinetics and Exposure Response in Patients Hospitalized With COVID-19: A Secondary Analysis of the ACTIV-1 IM Randomized Clinical Trial. JAMA Netw. Open *7*, e247615. https://doi.org/10.1001/jamanetworkopen.2024.7615.

16. Nakamura, S., Suzuki, K., Iijima, H., Hata, Y., Lim, C.R., Ishizawa, Y., Kameda, H., Amano, K., Matsubara, K., Matoba, R., and Takeuchi, T. (2016). Identification of baseline gene expression signatures predicting therapeutic responses to three biologic agents in rheumatoid arthritis: a retrospective observational study. Arthritis Res. Ther. *18*, 159. https://doi.org/10.1186/s13075-016-1052-8.

17. Tasaki, S., Suzuki, K., Kassai, Y., Takeshita, M., Murota, A., Kondo, Y., Ando, T., Nakayama, Y., Okuzono, Y., Takiguchi, M., et al. (2018). Multi-omics monitoring of drug response in rheumatoid arthritis in pursuit of molecular remission. Nat. Commun. *9*, 2755. https://doi.org/10.1038/s41467-018-05044-4.

18. Alivernini, S., MacDonald, L., Elmesmari, A., Finlay, S., Tolusso, B., Gigante, M.R., Petricca, L., Di Mario, C., Bui, L., Perniola, S., et al. (2020). Distinct synovial tissue macrophage subsets regulate inflammation and remission in rheumatoid arthritis. Nat. Med. *26*, 1295–1306. https://doi.org/10.1038/s41591-020-0939-8.

19. Wu, X., Liu, Y., Jin, S., Wang, M., Jiao, Y., Yang, B., Lu, X., Ji, X., Fei, Y., Yang, H., et al. (2021). Single-cell sequencing of immune cells from anticitrullinated peptide antibody positive and negative rheumatoid arthritis. Nat. Commun. *12*, 4977. https://doi.org/10.1038/s41467-021-25246-7.

20. Zhang, F., Jonsson, A.H., Nathan, A., Millard, N., Curtis, M., Xiao, Q., Gutierrez-Arcelus, M., Apruzzese, W., Watts, G.F.M., Weisenfeld, D., et al. (2023). Deconstruction of rheumatoid arthritis synovium defines inflammatory subtypes. Nature *623*, 616–624. https://doi.org/10.1038/s41586-023-06708-y.

21. Wei, K., Korsunsky, I., Marshall, J.L., Gao, A., Watts, G.F.M., Major, T., Croft, A.P., Watts, J., Blazar, P.E., Lange, J.K., et al. (2020). Notch signalling drives synovial fibroblast identity and arthritis pathology. Nature *582*, 259–264. https://doi.org/10.1038/s41586-020-2222-z.

22. Siegmund, D., and Wajant, H. (2023). TNF and TNF receptors as therapeutic targets for rheumatic diseases and beyond. Nat. Rev. Rheumatol. *19*, 576–591. https://doi.org/10.1038/s41584-023-01002-7.

23. Müller-Dott, S., Tsirvouli, E., Vazquez, M., Ramirez Flores, R.O., Badia-i-Mompel, P., Fallegger, R., Türei, D., Lægreid, A., and Saez-Rodriguez, J. (2023). Expanding the coverage of regulons from high-confidence prior knowledge for accurate estimation of transcription factor activities. Nucleic Acids Res. *51*, 10934–10949. https://doi.org/10.1093/nar/gkad841.

24. Jaaks, P., Coker, E.A., Vis, D.J., Edwards, O., Carpenter, E.F., Leto, S.M., Dwane, L., Sassi, F., Lightfoot, H., Barthorpe, S., et al. (2022). Effective drug combinations in breast, colon and pancreatic cancer cells. Nature *603*, 166–173. https://doi.org/10.1038/s41586-022-04437-2.

25. Feagan, B.G., Sands, B.E., Sandborn, W.J., Germinaro, M., Vetter, M., Shao, J., Sheng, S., Johanns, J., and Panés, J.; VEGA Study Group (2023). Guselkumab plus golimumab combination therapy versus guselkumab or golimumab monotherapy in patients with ulcerative colitis (VEGA): a randomised, double-blind, controlled, phase 2, proof-of-concept trial. Lancet Gastroenterol. Hepatol. *8*, 307–320. https://doi.org/10.1016/S2468-1253(22)00427-7.

26. Miyatani, Y., Choi, D., Choi, N.K., and Rubin, D.T. (2024). Dual-Targeted Therapy with Upadacitinib and Ustekinumab in Medically Complex Crohn's Disease. Dig. Dis. Sci. *69*, 355–359. https://doi.org/10.1007/s10620-023-08182-y.

27. The U.S. Food and Drug Administration (FDA), Advancing Health Through Innovation: New Drug Therapy Approvals 2021 (2021), https://www.fda.gov/files/drugs/published/2021_NewDrugTherapyApprovals_final_220126_0.pdf

28. Diallo, K., Simons, N., Sayegh, S., Baron, M., Degboé, Y., Boyer, J.-F., Kruglov, A., Nedospasov, S., Novarino, J., Aloulou, M., et al. (2021). Evidence for tmTNF reverse signaling *in vivo*: Implications for an arginase-1-mediated therapeutic effect of TNF inhibitors during inflammation. iScience *24*, 102331. https://doi.org/10.1016/j.isci.2021.102331.

29. Rivellese, F., Surace, A.E.A., Goldmann, K., Sciacca, E., Çubuk, C., Giorli, G., John, C.R., Nerviani, A., Fossati-Jimack, L., Thorborn, G., et al. (2022). Rituximab versus tocilizumab in rheumatoid arthritis: synovial biopsy-based biomarker analysis of the phase 4 R4RA randomized trial. Nat. Med. *28*, 1256–1268. https://doi.org/10.1038/s41591-022-01789-0.

30. Simon Andrews (2010). FastQC: A Quality Control Tool for High Throughput Sequence Data. http://www.bioinformatics.babraham.ac.uk/projects/fastqc/.

31. Chen, S., Zhou, Y., Chen, Y., and Gu, J. (2018). fastp: an ultra-fast all-in-one FASTQ preprocessor. Bioinformatics *34*, i884–i890. https://doi.org/10.1093/bioinformatics/bty560.

32. Smith, T., Heger, A., and Sudbery, I. (2017). UMI-tools: Modelling sequencing errors in Unique Molecular Identifiers to improve quantification accuracy. Genome Res. *27*, 491–499, gr.209601.116. https://doi.org/10.1101/gr.209601.116.

33. Liao, Y., Smyth, G.K., and Shi, W. (2014). featureCounts: an efficient general purpose program for assigning sequence reads to genomic features. Bioinformatics *30*, 923–930. https://doi.org/10.1093/bioinformatics/btt656.

CellPress
OPEN ACCESS

34. Love, M.I., Huber, W., and Anders, S. (2014). Moderated estimation of fold change and dispersion for RNA-seq data with DESeq2. Genome Biol. *15*, 550. https://doi.org/10.1186/s13059-014-0550-8.

35. Durinck, S., Spellman, P.T., Birney, E., and Huber, W. (2009). Mapping identifiers for the integration of genomic datasets with the R/Bioconductor package biomaRt. Nat. Protoc. *4*, 1184–1191. https://doi.org/10.1038/nprot.2009.97.

36. Kang, H.M., Subramaniam, M., Targ, S., Nguyen, M., Maliskova, L., McCarthy, E., Wan, E., Wong, S., Byrnes, L., Lanata, C.M., et al. (2018). Multiplexed droplet single-cell RNA-sequencing using natural genetic variation. Nat. Biotechnol. *36*, 89–94. https://doi.org/10.1038/nbt.4042.

37. Pool, A.-H., Poldsam, H., Chen, S., Thomson, M., and Oka, Y. (2023). Recovery of missing single-cell RNA-sequencing data with optimized transcriptomic references. Nat. Methods *20*, 1506–1515. https://doi.org/10.1038/s41592-023-02003-w.

38. Huang, X., and Huang, Y. (2021). Cellsnp-lite: an efficient tool for genotyping single cells. Bioinformatics *37*, 4569–4571. https://doi.org/10.1093/bioinformatics/btab358.

39. Huang, Y., McCarthy, D.J., and Stegle, O. (2019). Vireo: Bayesian demultiplexing of pooled single-cell RNA-seq data without genotype reference. Genome Biol. *20*, 273. https://doi.org/10.1186/s13059-019-1865-2.

40. Brand, D.D., Latham, K.A., and Rosloniec, E.F. (2007). Collagen-induced arthritis. Nat. Protoc. *2*, 1269–1275. https://doi.org/10.1038/nprot.2007.173.

41. Law, C.W., Chen, Y., Shi, W., and Smyth, G.K. (2014). voom: precision weights unlock linear model analysis tools for RNA-seq read counts. Genome Biol. *15*, R29. https://doi.org/10.1186/gb-2014-15-2-r29.

42. Hoffman, G.E., and Schadt, E.E. (2016). variancePartition: interpreting drivers of variation in complex gene expression studies. BMC Bioinf. *17*, 483. https://doi.org/10.1186/s12859-016-1323-z.

43. Sergushichev, A.A. (2016). An algorithm for fast preranked gene set enrichment analysis using cumulative statistic calculation. Preprint at bioRxiv. https://doi.org/10.1101/060012.

44. de Matos Simoes, R., and Emmert-Streib, F. (2012). Bagging Statistical Network Inference from Large-Scale Gene Expression Data. PLoS One *7*, e33624. https://doi.org/10.1371/journal.pone.0033624.

45. Hao, Y., Stuart, T., Kowalski, M.H., Choudhary, S., Hoffman, P., Hartman, A., Srivastava, A., Molla, G., Madad, S., Fernandez-Granda, C., and Satija, R. (2024). Dictionary learning for integrative, multimodal and scalable single-cell analysis. Nat. Biotechnol. *42*, 293–304. https://doi.org/10.1038/s41587-023-01767-y.

46. Hao, Y., Hao, S., Andersen-Nissen, E., Mauck, W.M., Zheng, S., Butler, A., Lee, M.J., Wilk, A.J., Darby, C., Zager, M., et al. (2021). Integrated analysis of multimodal single-cell data. Cell *184*, 3573–3587.e29. https://doi.org/10.1016/j.cell.2021.04.048.

47. Korsunsky, I., Millard, N., Fan, J., Slowikowski, K., Zhang, F., Wei, K., Baglaenko, Y., Brenner, M., Loh, P.R., and Raychaudhuri, S. (2019). Fast, sensitive and accurate integration of single-cell data with Harmony. Nat. Methods *16*, 1289–1296. https://doi.org/10.1038/s41592-019-0619-0.

48. Monaco, G., Lee, B., Xu, W., Mustafah, S., Hwang, Y.Y., Carré, C., Burdin, N., Visan, L., Ceccarelli, M., Poidinger, M., et al. (2019). RNA-Seq Signatures Normalized by mRNA Abundance Allow Absolute Deconvolution of Human Immune Cell Types. Cell Rep. *26*, 1627–1640.e7. https://doi.org/10.1016/j.celrep.2019.01.041.

49. Finak, G., McDavid, A., Yajima, M., Deng, J., Gersuk, V., Shalek, A.K., Slichter, C.K., Miller, H.W., McElrath, M.J., Prlic, M., et al. (2015). MAST: a flexible statistical framework for assessing transcriptional changes and characterizing heterogeneity in single-cell RNA sequencing data. Genome Biol. *16*, 278. https://doi.org/10.1186/s13059-015-0844-5.

50. Langfelder, P., and Horvath, S. (2008). WGCNA: an R package for weighted correlation network analysis. BMC Bioinf. *9*, 559. https://doi.org/10.1186/1471-2105-9-559.

51. Su, C., Xu, Z., Shan, X., Cai, B., Zhao, H., and Zhang, J. (2023). Cell-type-specific co-expression inference from single cell RNA-sequencing data. Nat. Commun. *14*, 4846. https://doi.org/10.1038/s41467-023-40503-7.

52. Purcell, S., Neale, B., Todd-Brown, K., Thomas, L., Ferreira, M.A.R., Bender, D., Maller, J., Sklar, P., de Bakker, P.I.W., Daly, M.J., and Sham, P.C. (2007). PLINK: A Tool Set for Whole-Genome Association and Population-Based Linkage Analyses. Am. J. Hum. Genet. *81*, 559–575. https://doi.org/10.1086/519795.

53. Kang, J.B., Nathan, A., Weinand, K., Zhang, F., Millard, N., Rumker, L., Moody, D.B., Korsunsky, I., and Raychaudhuri, S. (2021). Efficient and precise single-cell reference atlas mapping with Symphony. Nat. Commun. *12*, 5890. https://doi.org/10.1038/s41467-021-25957-x.

54. (2022). decoupleR: ensemble of computational methods to infer biological activities from omics data. Bioinformatics Advances. https://academic.oup.com/bioinformaticsadvances/article/2/1/vbac016/6544613?login=false.

STAR★METHODS

KEY RESOURCES TABLE

REAGENT or RESOURCE	SOURCE	IDENTIFIER
**Antibodies**		
Anti-CD14-APC (clone 61D3)	eBioscience	Cat#14-0149-82; RRID: AB_467129
Anti-HLA-DRB1-PE-Vio	Miltenyi Biotec	Cat#MA110354; RRID: AB_11152797
Human IgG isotype control	Invitrogen	Cat#31154
In vivo mAb anti-mouse IL6R clone 15A7	BioXCell	#BE0047
Enbrel (Etanercept)	Pfizer	N/A
Rat IgG2b	This paper	N/A
Mouse IgG1	This paper	N/A
**Bacterial and virus strains**		
Mycobacterium tuberculosis H37Ra	Fisher Scientific	10218823
pLV[Exp]-CMV>{3/11-ratIgG2b}:IRES:Cerulean	Vector Builder	N/A
pLV[Exp]-CMV>{3/11-ratKappa}:IRES:EGFP	Vector Builder	N/A
pLV[Exp]-CMV>{D44.1-mIgG1}:IRES:Cerulean	Vector Builder	N/A
pLV[Exp]-CMV>{D44.1-mIg Kappa}:IRES:EGFP	Vector Builder	N/A
**Biological samples**		
Whole blood (PAXgene-preserved)	Study cohorts	Not applicable
ACD-preserved whole blood	Study cohorts	Not applicable
Peripheral blood mononuclear cells (PBMCs, cryopreserved)	RA and healthy donors cohorts	Not applicable
**Chemicals, peptides, and recombinant proteins**		
DNase I	Worthington Biochemical	PN LS002007
PBS (1×)	Thermo Fisher Scientific	Cat#20012027
BSA (0.05% for PBS supplement)	Miltenyi Biotec	PN 938130-091-376
RPMI medium	Gibco	Cat#61870-036
FBS	Thermo Fisher Scientific	Cat#A5209501
Trypan Blue	Used with TC20 counter	Cat#15250061
Immunization Grade Bovine Collagen II, 10mg lyophilized	Chondrex	20021
Immunization Grade Bovine Collagen II, 2mg/mL	Chondrex	20022
Frend's Adjuvant, Complete	Sigma	F5881
Freund's Adjuvant, Incomplete	Sigma	F5506
L-Glutamine Solution (200 mM)	Cultek	Cat#H3BE17-605E
Gentamycin	Lonza	Cat#91L0012-010
Cell Boost 7a	HyClone	Cat#SH31026.07
Cell Boost 7b	HyClone	Cat#SH31027.01
Recombinant human M-CSF	PeproTech	Cat#300-25-10UG
Tumor necrosis factor-α (TNF-α)	Bio-Techne R&D Systems	Cat#210-TA-020
Etanercept (Erelzi)	Sandoz	N/A
DMSO	Merck_Sigma-Aldrich	Cat#D2650-100ML
Lymphoprep™	StemCell Technologies	Cat#18060
Ficoll Paque-Plus	Cytiva	Cat#17544203
Monocyte Attachment Medium (MAM)	Sigma-Aldrich	Cat#C-28051
TrypLE™ Select	Gibco	Cat#12563011
Bovine Serum Albumin, Fraction V	Gibco	Cat#15260-037

(*Continued on next page*)

REAGENT or RESOURCE	SOURCE	IDENTIFIER
Continued		
Lipopolysaccharide (LPS)	eBioscience	Cat#00-4976-93
PBS w/o Ca2+ w/o Mg2+	Corning	Cat#21-040-CVR
Acridine Orange/Propidium Iodide Stain	Logos Biosystems	Cat#F23001
Critical commercial assays		
EasySep^{TM} Human Monocyte Isolation Kit	StemCell Technologies	Cat#19359
Chromium GEM-X Single Cell 3′ Reagent Kits v4	10× Genomics	Cat#1000691
Chromium GEM-X Single Cell 3′ Chip Kit v4	10× Genomics	Cat#1000690
Parse Evercode WT Mega Kit	Parse Biosciences	Cat#ECWT3500
Parse Cell Fixation Kit	Parse Biosciences	Cat#ECF3300
PAXgene Blood miRNA kit	QIAGEN	Cat#763134
Quant-iT RiboGreen RNA Ki	Thermo Fisher Scientific	Cat#R11490
KAPA RNA HyperPrep Kit with RiboErase (HMR) Globin	Roche	Cat#KK8563
xGen^{TM} UDI-UMI Adapters	IDT	Cat#10005903
Chromium Next GEM Single Cell 3′ Reagent Kit v3.1	10× Genomics	PN-1000121
3′ CellPlex Kit Set A	10× Genomics	N/A
Omni 2.5–8 BeadChip (genotyping array)	Illumina	N/A
Protein G chromatography (for Ig purification)	N/A	N/A
BCA assay (protein quantification)	N/A	N/A
Deposited data		
Longitudinal cohorts, bulk RNA-seq	This paper	EGA: EGAD50000002834
Single-cell RNA-seq, baseline RA and healthy controls	This paper	EGA: EGAD50000001964
Single-cell RNA-seq, RA follow-up	This paper	EGA: EGAD50000002832
Single-cell RNA-seq, *in vitro* monocyte-derived macrophages	This paper	EGA: EGAD50000002833
Processed RNAseq, scRNAseq and metadata	This paper	Zenodo: https://doi.org/10.5281/zenodo.14999133
Experimental models: Cell lines		
Hamster: CHO-S cells	Thermo Fisher Scientific	Cat#R80007
Experimental models: Organisms/strains		
Mouse: DBA/1JRj	Janvier Labs	N/A
Oligonucleotides		
Cell Multiplexing Oligos (CMO)	10× Genomics	Included with 3′ CellPlex Kit Set A, PN 1000261
Software and algorithms		
Original analysis code (this paper)	This paper	Zenodo: https://doi.org/10.5281/zenodo.21476753
R 4.5.2	Open-source	https://cran.r-project.org/
FASTQC 0.12.1	Open-source	https://www.bioinformatics.babraham.ac.uk/projects/fastqc/
STAR 2.6.1day	Open-source	https://doi.org/10.1093/bioinformatics/bts635
Fastp 0.20.0	Open-source	https://doi.org/10.1093/bioinformatics/bty560
UMI-tools 1.1.6	Open-source	https://doi.org/10.1101/051755
FeatureCounts 1.5.3	Open-source	https://doi.org/10.1093/bioinformatics/btt656
DESeq2 (VST) 1.42.0	Open-source	https://bioconductor.org/packages/DESeq2/
BiomaRt 2.56.1	Open-source	https://bioconductor.org/packages/biomaRt/
Cell Ranger 7.0.1	10× Genomics	https://www.10xgenomics.com/support/software/cell-ranger/latest
cellsnp-lite 1.2.2	Open-source	https://doi.org/10.1093/bioinformatics/btab358

(*Continued on next page*)

Continued		
REAGENT or RESOURCE	SOURCE	IDENTIFIER
vireo 0.5.6	Open-source	https://doi.org/10.1186/s13059-019-1865-2
PLINK 2	Open-source	https://doi.org/10.1086/519795
LiftOver	Open-source	https://genome.ucsc.edu/cgi-bin/hgLiftOver
Seurat 5.0.0	Open-source	https://doi.org/10.1016/j.cell.2021.04.048
BPCells 0.1.0	Open-source	https://bnprks.github.io/BPCells
Harmony 1.2.0	Open-source	https://doi.org/10.1038/s41592-019-0619-0
Azimuth 0.5.0	Open-source	https://azimuth.hubmapconsortium.org/
Fgsea 1.28.0	Open-source	https://bioconductor.org/packages/fgsea
limma-voom	Open-source	https://bioconductor.org/packages/limma
variancePartition	Open-source	https://bioconductor.org/packages/variancePartition
MAST 1.28.0	Open-source	https://bioconductor.org/packages/MAST
WGCNA 1.7.-5	Open-source	https://cran.r-project.org/web/packages/WGCNA
CSCORE 0.0.0.9	Open-source	https://doi.org/10.1038/s41467-023-40503-7
DecoupleR 2.9.7	Open-source	https://doi.org/10.1093/bioadv/vbac016
CollecTRI TF network	Open-source	https://doi.org/10.1093/nar/gkad841
Symphony 0.1.1	Open-source	https://doi.org/10.1038/s41467-021-25957-x
Parse pipeline 1.1.0	Parse Biosciences	https://www.parsebiosciences.com/pipeline-download/
Other		
BD FACSAria™ Fusion Cell Sorter	BD Bioscience	N/A
LUNA-FL™ Dual Fluorescence Cell Counter	Logos Biosystems	Cat#L20001
Bioanalyzer High Sensitivity chip	Agilent Technologies	RRID:SCR_018043
Illumina NovaSeq X Plus 25B sequencer	Illumina	RRID:SCR_024568
Illumina NovaSeq 6000	Illumina	RRID:SCR_016387
Leucosep tubes	Greiner Bio-One	N/A
Falcon tube	Corning	Cat#352096
40 µm cell strainer	Cell Strainer	PN 43-10040-70
TC20™ Automated Cell Counter	Bio-Rad	RRID:SCR_025462

EXPERIMENTAL MODEL AND STUDY PARTICIPANT DETAILS

Patients and clinical sample collection

The patients analyzed in this study were recruited by the Immune-Mediated Inflammatory Diseases Consortium (IMIDC). Longitudinal cohorts of IMID patients under different targeted therapies were collected at the rheumatology, gastroenterology and dermatology departments from 52 different university hospitals in Spain. For the present study, 8 responders and 8 non-responders were selected for each of the 11 IMID and targeted therapy pairs considered (total n = 176 patients). Patient selection and therapeutic response criteria were defined by the DoCTIS clinical experts of each disease area (Supplemental Document S1). Applying these criteria, the final set of 16 patients were systematically selected to ensure optimal matching between responder and non-responder groups across epidemiological and clinical covariates (see patient selection and covariate balancing). Peripheral blood samples were obtained from all patients at enrollment, when therapy with a targeted therapy was initiated (week 0), and at the follow-up time point when the therapeutic response was assessed (weeks 12–16, Supplemental Methods). In addition, 8 healthy controls contributed clinical and blood samples at a single time point. Blood for RNA-Seq analysis was collected and preserved at −80°C using RNA-stabilizing PaxGene tubes (PreAnalytiX, Switzerland). Written informed consent was obtained from all participants.

The study was approved by the Hospital Universitari Vall d'Hebron Clinical Research Ethics Committee with reference number PR(AG)144/2019. This study was conducted according to the principles of the Declaration of Helsinki. Protocols were reviewed and approved by the local institutional review board of each participating center.

Collagen-induced arthritis mouse model

8-week-old DBA1/Jr mice purchased from Janvier Labs were housed in SPF conditions. Therapeutic groups consisted of anti-mIL6R clone 15A7 (BioXCell, #BE0047) administered at 0.2 mg/dose (i.p.), Etanercept administered at 0.1 mg/dose (i.p.), Anti-mIL6R clone

15A7 0.2 mg/dose (i.p). + Etanercept 0.1 mg/dose (i.p.) and rat IgG2b 0.2 mg/dose (i.p.) + mouse IgG1 0.1 mg/dose (i.p) as Isotype Control group. All treatments were administered 6 times over the course of 2 weeks. All experimental procedures were approved by the Ethical Committee of the University of Barcelona with protocol # CEEA 32/20.

METHOD DETAILS

Bulk RNA-seq

Total RNA was extracted using the PaxGene blood isolation kit (Qiagen). RNA was quantified using the RiboGreen assay. The median RIN and DV200 scores were 7 and 85%, respectively, supporting a high quality of the samples and the downstream synthesis of the RNA-Seq libraries. Ribosomal and globin RNA content was reduced using the Roche KAPA RNA HyperPrep Kit with Riboerase (HMR). To improve gene detection and quantification and minimize PCR bias, Unique Molecular Identifiers (UMIs) were added during library preparation. PCR duplicates derived from the same mRNA molecule were labeled and eliminated. Libraries were sequenced using the Novaseq6000 platform (Illumina), and a minimum of 50 million 100 bp paired-end reads per sample were obtained. To minimize batch effect on tested conditions (time point, IMID, therapy, and response), samples were randomized in different plates and batches (Figure S12A).

Global quality control (QC) was performed with FASTQC.[30] Reads were filtered by quality and trimmed using fastp.[31] High quality sequences were then aligned against the human genome (version GRCh38) with STAR. PCR duplicates were collapsed and removed with the adjacency method from umitools.[32] Sequences that mapped against the human genome were assigned to genes using featureCounts,[33] setting exons as features. Genes were considered expressed if they had at least one count in more than 80% of all samples. This yielded a total of 25,823 genes for downstream analyses. The count matrix was normalized for downstream analysis using VST from the DESeq2 package.[34] Sample outliers were not observed based on principal component analysis of the VST-transformed data. A moderate association with plate/sequencing batch was observed only for PC4 and PC7 (Figure S12B). This potential confounder was accounted for by including the plate indicator variable in the linear models used for differential expression analyses.

Whole blood bulk transcriptomic data

Datasets GSE78068[16] and GSE93272[17] were downloaded from the NCBI Gene Expression Omnibus (GEO). The data was already normalized, and the gene ids were re-mapped to Ensembl using the biomaRt R package.[35] The binary non-response clinical phenotype included in GSE78068 was used to compute anti-TNF and anti-IL6R non-response signatures in RA patients before treatment with Infliximab and Tocilizumab. GSE93272 was used to compute anti-TNF and anti-IL6R longitudinal drug effects by comparing the follow-up and baseline samples from the same patients. Follow-up samples were included only if collected within a 3-week window around week 12, to ensure alignment with the RA follow-up time point in the DoCTIS cohorts.

Single-cell RNA-seq

Human blood samples were collected in EDTA tubes (BD Biosciences). PBMCs were isolated using Ficoll density gradient centrifugation. For each condition, 12 mL of blood was diluted with an equal volume of pre-warmed RPMI 1640 culture medium (Lonza). The diluted blood was then carefully layered onto a Leucosep tube (Greiner Bio-One) prefilled with 15 mL of Ficoll-Plus (GE Healthcare Biosciences AB) and centrifuged for 15 min at 800×g and RT (without acceleration and brake). After centrifugation, PBMCs were collected with a sterile Pasteur pipette into a 50-mL tube, diluted up to 10 mL with pre-warmed RPMI medium and centrifuged for 10 min, at 400×g and RT. Following a second washing step with 5 mL of RPMI medium and a 5-min centrifugation, PBMCs were resuspended in 8 aliquots of freezing media. Freezing media consisted of RPMI 1640 with 20% heat-inactivated fetal bovine serum (Sigma-Aldrich), 10% DMSO (Sigma-Aldrich), and penicillin-streptomycin 1:1000 (Lonza). One-milliliter aliquots, with approximately 1 × 10e6 cells/mL, were gradually frozen using a commercial freezing box (Mr. Frosty, Nalgene) at − 80°C for 24 h and then stored in a vapor-phase liquid nitrogen tank at − 150°C.

Cryopreserved PBMCs were thawed in a water bath at 37°C and transferred to a 15 mL Falcon tube containing 10 mL of pre-warmed RPMI media supplemented with 10% FBS (Thermo Fisher Scientific). Samples were centrifuged at 350 × g for 8 min at RT, supernatant was removed, and pellets resuspended with 1 mL of cold 1× PBS (Thermo Fisher Scientific) supplemented with 0.05% BSA (PN 130-091-376, Miltenyi Biotec). Samples were incubated for 10 min at RT with 0.1 mg/mL of DNAse I (PN LS002007, Worthington-Biochem) to eliminate ambient DNA and favor the resuspension of the pellet. Cells were filtered with a 40 μm strainer (PN 43-10040-70, Cell Strainer) to remove eventual clumps and washed by adding 10 mL of cold PBS+0.05% BSA. Samples were centrifuged at 350 × g for 8 min at 4°C and resuspended in an adequate volume of PBS+0.05% BSA in order to reach the desired concentration. Cells concentration and viability were verified with a TC20 Automated Cell Counter (Bio Rad) upon staining of the cells with Trypan Blue.

Sample multiplexing was performed using the genotype approach described previously.[36] Genome-wide genotype data was generated for each individual using the Omni 2.5–8 BeadChip and Illumina GSA MG v.3-24 arrays (Illumina, USA). Quality control analysis was performed using PLINK software. In this analysis, we identified autosomal SNPs and, using those SNPs from chromosome X, we confirmed the consistency between SNP-estimated and the clinically reported sex. Then, we quantified the percentage of SNPs with a minor allele frequency (MAF) higher than 5%. The percentage of missingness both at the SNP-wise and sample-wise levels was computed. We assessed the heterozygosity rate (F) of each sample to evaluate if any of the genotyped samples could

be contaminated. Patient genotypes were simplified by removing Single Nucleotide Variants (SNVs) that were unannotated (chr 0), located in the sexual Y (chr 24), pseudo-autosomal XY (chr 25), or mitochondrial chromosomes (chr 26). As genotypes were obtained using the human hg19 reference genome, we converted their coordinates to the same reference genome used to mapped the sequencing reads (GRCh38), via the UCSC LiftOver tool.

PBMCs samples were evenly mixed in pools of 8 donors. To avoid technical artifacts that could mask biological differences, samples with different response to treatment were pooled in the same library. Multiplexed samples were loaded into the 10× Genomics Chromium Controller, loaded into two channels with a target recovery of 35,000 cells per channel (to ensure at least 3,000 cells per sample condition). Libraries were prepared using a standard throughput Chromium Next GEM Single Cell 3′ Reagent Kit v3.1 and following manufacturer's instructions of protocol CG000390. More specifically, cells were partitioned into Gel BeadInEmulsions (GEMs) and underwent a GEM-RT incubation resulting in cDNAs that are further purified with SPRI beads. cDNA initial product was then pre-amplified during 13 cycles using the Dual Index Plate TT Set A. Between 20 and 200 ng of cDNA were used for preparing libraries and final library size distribution and concentration were determined using a Bioanalyzer High Sensitivity chip (Agilent Technologies). Library sequencing was carried out on a NovaSeq6000 system (Illumina) using the following sequencing conditions: 28 bp (Read 1) + 10 bp (i7 index) + 10 bp (i5 index) + 90 bp (Read 2), to obtain approximately 20,000 paired-end reads per cell.

Raw reads were fed into 10× Genomics Inc. software CellRanger (v.7.0.1) to obtain gene count matrices. Sequences were mapped against the optimized human genome reference (version 1.0 from GRCh38 reference) following Pool et al.[37]

Multiplexed libraries were demultiplexed with cellsnp-lite (v 1.2.2)[38] in Mode 1a, which allows genotyping of single-cell GEX libraries by piling-up the expressed alleles based on a list of given SNPs. To do so, we used a list of 7.4 million common SNPs in the human population (MAF >5%) published by the 1000 Genome Project consortium. Donor deconvolution was performed using vireo (v 0.5.6),[39] which assigns the deconvoluted samples to its donor identity using known genotypes, while detecting doublets and unassigned cells. Finally, we discarded detected doublets and unassigned cells before moving on to the downstream processing steps.

Single-cell RNA-seq analysis of monocyte-derived macrophages *in vitro*

PBMCs were obtained by Ficoll density gradient centrifugation (Lymphoprep^{TM}, Stem Cell Technologies; Ficoll Paque Plus, Cytiva) from fresh whole blood. Before cell sorting, CD14^{+}CD16- monocyte population was enriched by immunomagnetic negative selection using the EasySep Human Monocyte Isolation Kit (StemCell Technologies). Isolated monocytes were stained with conjugated CD14-APC (clone 61D3 eBioscience), HLA-DRB1-PE-Vio (Miltenyi). Cells were separated in CD14+/CD16- and HLA-DRB1-negative or HLA-DRB1^{Hi} (Figure S13) using a BD FACSAria Fusion Cell Sorter (BD Bioscience).

HLA-DRB1-negative and HLA-DRB1^{Hi} monocyte subpopulations were resuspended in Monocyte Attachment Medium (MAM) (Sigma-Aldrich) supplemented with M-CSF (Peprotech) at 100 ng/mL, seeded in a 24 well plate and incubated for 1.5h in 5% CO2 incubator at 37°C to allow monocytes to adhere to the plate. Upon monocyte adhesion, media was changed to RPMI supplemented with 10%FBS and M-CSF at 100 ng/mL and cultured for 7 days. For single-cell analysis, *in vitro* differentiated macrophages were detached on day 7 with TrypLE Select (Gibco) and washed with PBS. Cell number and viability were assessed using a LUNA-FL Dual Fluorescence Cell Counter (Logos Biosystems).

Cells were encapsulated using Chromium GEM-X Single-cell 3′ Reagent Kits v4 targeting a recovery of 5,000 cells/sample. Libraries were prepared following manufacturer's instructions of protocol CG000732 (10× Genomics). Between 20 and 200 ng of cDNA were used for library preparation, and the final library size distribution and concentration were determined using a Bioanalyzer High Sensitivity chip (Agilent Technologies). Libraries were sequenced aiming at a minimum coverage of 40,000 raw reads per cell on Illumina NovaSeq X Plus 25B sequencer. Raw reads were fed into 10× Genomics Inc. software CellRanger (v.7.0.1) to obtain gene count matrices. Sequences were mapped against the optimized human genome reference (version 1.0 from GRCh38 reference) following Pool et al.[37]

In vitro characterization of anti-TNF effect in PBMCs

Single-cell transcriptomes from PBMCs from six RA samples were treated *in vitro* under four different conditions and profiled with the Parse Evercode WT Mega kit (Parse Biosciences, Seattle, WA, USA). PBMCs were previously isolated from six RA whole blood by Ficoll density gradient centrifugation using the standard protocol described above. Isolated PBMCs were then stored in freezing media (FBS with 10% DMSO) in liquid nitrogen. Thawed PBMCs were pre-incubated with or without 10 ng/mL TNF for 2h at 37°C. Next, both conditions were treated with either 5 μg/mL Etanercept or isotype control (ISO) for 24h at 37°C. Cells were then fixed and permeabilized as indicated by the manufacturer (Parse Bioscience Fixation protocol V2.0.2). Fixed cells were subsequently barcoded, and libraries were prepared following manufacturer's instructions. Each library was sequenced using Illumina's NovaSeq 6000 platform, targeting a minimum of 20,000 150-bp paired-end reads per cell.

CIA mouse model

To induce arthritis, bovine collagen II (bCII) (Chondrex) dissolved in acetic acid 0.05M was emulsified 1:1 (v/v) in CFA containing 4 mg/mL heat-inactivated Mycobacterium tuberculosis (H37Ra). DBA1/Jr mice were immunized at the base of the tail with 100 μg of bCII in CFA (Sigma) and boosted on day 17 with 100 μg of bCII in IFA (Sigma) in the flanks. Size of the four paws of each animal was measured with a caliper before immunization day (day 0) and every two days upon disease onset. Disease progression was

CellPress
OPEN ACCESS

measured as percentage increase in joint swelling relative to day 0 and by clinical score according to.[40] When percentage increase in joint swelling reached 130% of the day 0 value, mice were randomly assigned into therapeutic groups. Both male and female DBA1/Jr mice were used in all the therapeutic groups.

Antibody production

Rat IgG2b and mouse IgG1 antibody production for the CIA mouse model Isotype control group were purified from supernatants of CHO-S cells (Invitrogen, San Diego CA) transduced with lentivirus encoding for light and heavy chains of each immunoglobulin. After FACS selection of highly transduced cells, sorted cells were harvested and the antibodies were purified via protein G chromatography, and quantified by BCA assay. All cell lines were routinely tested for Mycoplasma contamination. CHO-S cells were obtained from Thermo Fisher. The transduced CHO-S lines were authenticated in-house by PCR.

QUANTIFICATION AND STATISTICAL ANALYSIS

Patient selection and covariate balancing

For each drug-disease cohort, we selected 16 patients, prioritizing the selection of 8 top responders and 8 top non-responders. Top responders/non-responders were defined based on the change in the established disease activity score. To obtain a matched comparison set of patients from the complementary group, we chose the set of patients that minimized the marginal differences in the following covariates: sex (as recorded in the clinical record), age, body mass index, years of evolution, smoking status, concomitant use of other DMARDs and baseline disease activity score. Differences were measured based on the P-value of the *t* test for quantitative covariates and based on the difference in prevalence for binary covariates and considered as optimal set the one that minimized the sum of these statistics.

Only for PS-AntiIL17 and IBD-AntiIL12IL23 there were not enough responders/non-responders in one of the two groups. For PS-AntiIL17, we selected all the available patients meeting the non-response criteria ($n = 5$) and matched them against an optimal set of $n = 11$ responders to make up for a total of 16 patients. Similarly, for the IBD-*anti*-IL12p40 cohort, we selected all available responders ($n = 7$) and matched them against an optimal set of $n = 9$ non-responders.

Gene expression signatures

To determine the non-response signature, we performed differential expression analysis between responders and non-responders in each cohort at baseline. Non-response signatures were defined at baseline because this contrast isolates pre-treatment biological differences between responders and non-responders. In contrast, post-treatment NR–R differences reflect a mixture of baseline biology, drug-induced transcriptional changes, and divergence in disease activity during therapy, and therefore do not represent intrinsic mechanisms of resistance. We performed differential expression analysis using the limma-voom framework.[41] The comparisons were adjusted for batch (plate indicator) when applicable. To characterize the longitudinal effects of drugs we compared the gene expression levels between baseline and post-treatment time points in each cohort. Random effects models were considered to account for individual patient effects in the data. We used the limma-voom framework implementation of the random effects model provided by the variancePartition R package.[42] To enhance the generalizability of the generated signatures, treatment effects were obtained using all patients where each drug was tested. If multiple diseases were included, a disease indicator was added as an adjustment variable. Comparisons were adjusted for batch (plate indicator) when applicable as fixed effects, and the subject indicator was modeled as a random effect.

The molecular signatures used to evaluate drug complementarity were defined as the signed logarithm (log10) of the P-values from the corresponding differential gene expression analysis, with the sign reflecting the direction of the log fold change. A positive sign was assigned to indicate an increased expression over time for drug effects and an increased expression in non-responders versus responders for clinical response.

Analysis of drug combinations using MNRS

Our working hypothesis to address the optimal drug combination problem is that an effective pairing of drugs A and B occurs when the effects of drug B shift a patient's transcriptomic profile toward that of responders to drug A, and vice versa.

To nominate pairs of targeted drugs with the higher potential for combination therapy, we leveraged both the drug effect and the clinical response molecular signatures observed in the different drug-disease cohorts of the DoCTIS project. We adopted the following framework based on the MNRS working hypothesis: 1) All longitudinal drug effects signatures were considered (in the case where the drug was used in more than one IMID cohorts, we grouped them to improve the estimation of the drug-effect signature). 2) Consider all signatures of non-response to the rest of drugs. 3) For each pair of drug effects and non-response signatures, select only genes that are significant for both based on a pre-defined association threshold (e.g., $p < 0.05$). 4) Compute Pearson's correlation between the drug effects signature and the non-response signature and store the results in a matrix M_{ij} where *i* is the index for the non-response signature and *j* the index for the drug effect signature. Using the described workflow, we obtained a signature correlation matrix, which we used to identify the most promising drug-disease partner for each drug by selecting the most negative correlations in each column. To further characterize the complementarity detected by the correlations, we computed the percentages of non-response genes that were reversed and antagonized by the drugs to be combined.

Biological characterization of the drug non-response and drug-effect signatures

To identify the biological processes antagonized by the proposed combinations of drugs, we performed functional enrichment analyses using as reference the collection of biological processes (BP) collected by Gene Ontology (GO) project (version October 2023). To convert gene-level signatures to gene set-level signatures we applied the rank-based enrichment test using Gene Set Functional Enrichment Analysis (GSEA) tool implemented in the fgsea library.[43] For each set of differentially expressed genes, we multiplied the sign of the coefficient by the minus logarithm of the P-value to obtain the statistic used to rank the genes and perform the enrichment. To assess the enrichment of selected sets of genes (e.g., genes that were associated with non-response and modulated in the same direction by the other therapy), we applied a hypergeometric test.[44]

Single-cell RNA-seq of the DoCTIS cohort

Mapped reads against the optimized version of the human genome were analyzed with Seurat (v.5.0.0).[45] Cells with less than 750 UMIs or less than 300 genes were discarded. Cells with a mitochondrial content higher than 20% were also filtered out, as well as genes expressed in less than 15 cells. A total of 590,872 cells and 36,387 genes were considered for downstream analyses. Gene normalization, selection, and scaling were performed following Zhang et al.[20] Features were log-normalized before selecting the 1,000 most highly variable genes (HVGs) per sample. Cell cycle genes listed in *Seurat::cc.genes* were excluded. All HVGs were pooled together to perform a sketch-based analysis using seurat in combination with BPCells. Major cell lineages (myeloid, NK-T, and B cells) were annotated after mapping to the PBMC reference dataset included in the distribution package.[46] Gene normalization, selection, scaling, and cells clustering were then performed per each lineage separately. After integrating the data by sample using Harmony,[47] UMAPs were performed. For each cluster, we obtained the most significantly upregulated genes in comparison to the rest of cells in the lineage using the *Seurat::FindAllMarkers* function. Myeloid, B and plasma cells were manually annotated by inspecting the resulting markers as well as the predicted annotation using Azimuth[46] at different resolutions. For T and NK subtype annotation we used the same approach but also included an estimation of the T/NK subtypes as defined in Monaco et al.[48] using GSEA. For this latter approach, candidate annotations were assigned based on the significance of the enrichment results.

To evaluate changes in cell composition after therapy we modeled the abundance of each cell type using a linear mixed model, including donor as a random effect. Drug-specific differentially expressed genes between non-responders (NR) and responders (R), at baseline and follow-up, and between two timepoints (NR + R), were tested per each cell type using MAST.[49] The fixed-effects model implemented in MAST included adjustment by sex, age, cellular rate detection, and percentage of mitochondrial gene expression. To adjust for the donor, a random effect was also added to the model. Cellular rate detection was estimated by counting the total number of different genes expressed by a cell (minimum one UMI) and scaling this value from 0 to 1 across all cells. Significant changes in cell composition were tested by using a linear mixed model implemented in the lmerTest package, including random effect by donor.

To infer the cell-type specific co-expression module associated with HLA-DRB1 expression, we applied the WGCNA.[50] For this, we first computed CSCORE estimates of gene-coexpression. CSCORE[51] provides an alternative measure of gene co-expression for single-cell RNA-sequencing data, replacing the Pearson correlations used in the traditional WGCNA workflow. Only CSCORE estimates with a significant P-value (FDR <0.05) were considered for WGCNA analysis.

Genotyping for scRNA-Seq demultiplexing

Genome-wide genotyping data from 48 RA patients and 8 healthy controls was generated using the Illumina Omni2.5-8 BeadChip array (Illumina, USA) at HudsonAlpha Institute for Biotechnology (Alabama, US). A total of 2,619,926 genetic variants (mostly SNPs, but also structural variants) were genotyped. Quality control analysis was performed using PLINK.[52] All individuals had >90% of genotyped SNPs. All SNPs (n = 82,274 SNPs) with a missingness rate greater than 5% were excluded for downstream analyses.

Single-cell RNA-seq of *in vitro* response to anti-TNF therapy

Reads were mapped and demultiplexed following the bioinformatic pipeline developed by Parse (version 1.1.0). Downstream analyses were performed with Seurat. Cells with less than 750 counts or less than 300 genes were discarded. Cells with a mitochondrial content higher than 20% were excluded. Only genes expressed in more than 15 cells were considered for downstream analyses. A total of 34,984 cells and 32,091 genes were analyzed. Gene normalization, selection, and scaling were performed as previously described. Cells were clustered and manually annotated according to the top markers of each Louvain cluster. The list of the top markers per cluster was extracted using the *Seurat::FindMarkers* function.

Single-cell RNA-Seq analysis of PBMCs and synovial biopsies from RA patients

Single-cell RNA-Seq data from paired PBMCs and synovial membrane biopsies of 17 RA patients were downloaded from the Genome Sequence Archive (HRA000155).[19] Raw reads were fed into 10× Genomics Inc. software CellRanger (v.7.0.1) to obtain gene count matrices. Sequences were mapped against the optimized human genome reference (version 1.0 from GRCh38 reference) following Pool et al.[37] Cells with less than 500 UMIs or less than 250 genes were discarded. Cells with more than 20% mitochondrial expressed genes were not considered, and only genes expressed in more than 15 cells were included. A total of 290,346 PBMCs and 165,605 cells from the synovial membrane were analyzed. Symphony[53] was used to annotate PBMCs against our reference from the DoCTIS cohort. Synovial membrane cells were mapped against the RA synovial membrane atlas generated by the AMP.[20]

Single-cell RNA-Seq analysis of *in vitro* differentiated HLA-DRB1-negative/High monocytes

Mapped reads against the optimized version of the human genome were analyzed with Seurat (v.5.0.0).[45] Cells with less than 350 UMIs or less than 250 genes were discarded. Cells with a mitochondrial content higher than 20% were also filtered out, as well as genes expressed in less than 15 cells. A total of 5,190 cells and 15,381 genes were considered for downstream analyses. Features were log-normalized before selecting the 1,000 most highly variable genes (HVGs) per sample. Cell cycle genes listed in *Seurat::cc.genes* were excluded. All HVGs were pooled together, obtaining a list of 1,243 genes. Gene normalization, selection, and scaling were performed following Zhang et al.[20] Differentially expressed genes between HLA-DRB1+ and HLA-DRB1- derived macrophages were tested using MAST adjusting by cellular rate detection and percentage of mitochondrial gene expression.

TF activity

decoupleR[54] was used to infer the activity of the 1,186 human TFs included in the CollecTRI database.[23] The univariate linear model (ulm) method was run to infer TF enrichment scores per cell. Scores were scaled from 0 to 1 across all cells. To test for a significant association between TF activity and cell cluster, a linear mixed model was used with donor as random effect. TF activity scores were correlated with *HLA-DRB1* expression using Pearson's correlation test.

Cell Reports Medicine, Volume *7*

Supplemental information

Uncovering combination therapies for immune-mediated inflammatory diseases through systems biology analysis on longitudinal patient data

Sergio H. Martínez-Mateu, Yolanda Guillén, Edgar Angelats, María A. López-Lasanta, Stefania Madonna, Laura Jiménez-Gracia, Ivan Rodriguez-Nunez, Damiana Álvarez-Errico, Adrià Aterido, Raül Tortosa, Max Ruiz, Pau Serra, Juan D. Cañete, Jose M. Carrascosa, Eugeni Domènech, Javier P. Gisbert, Jesús Tornero, IMID-Consortium, Britta Siegmund, Giampiero Girolomoni, Ernest H.S. Choy, Richard M. Myers, Holger Heyn, Pere Santamaria, Sara Marsal, and Antonio Julià

SUPPLEMENTAL FIGURES

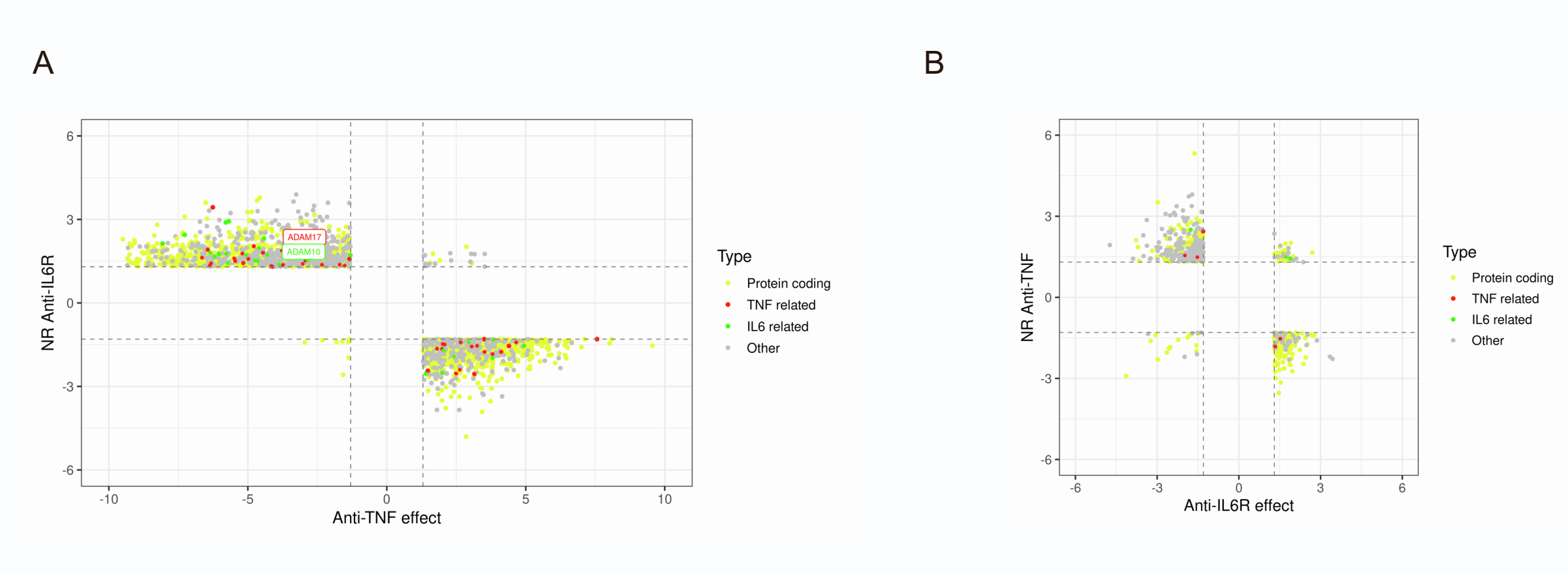

Figure S1. MNRS analysis shows antiIL6R-antiTNF complementarity in RA patients. Plots comparing the non-response signature of one drug (y-axis) with the longitudinal effect of the complementary drug (x-axis). Gene values are shown as log10(P-value) of differential gene expression with the sign of the fold change. (A) Anti-IL6R non-response signature against anti-TNF longitudinal effect. (B) Anti-TNF non-response signature against anti-IL6R longitudinal effect. Colours indicate if the gene is from TNF signalling pathway (red), from IL-6 signalling pathway (green) or, otherwise, if it’s a protein-coding gene (yellow) or non-coding gene (grey). Related to Figure 2.

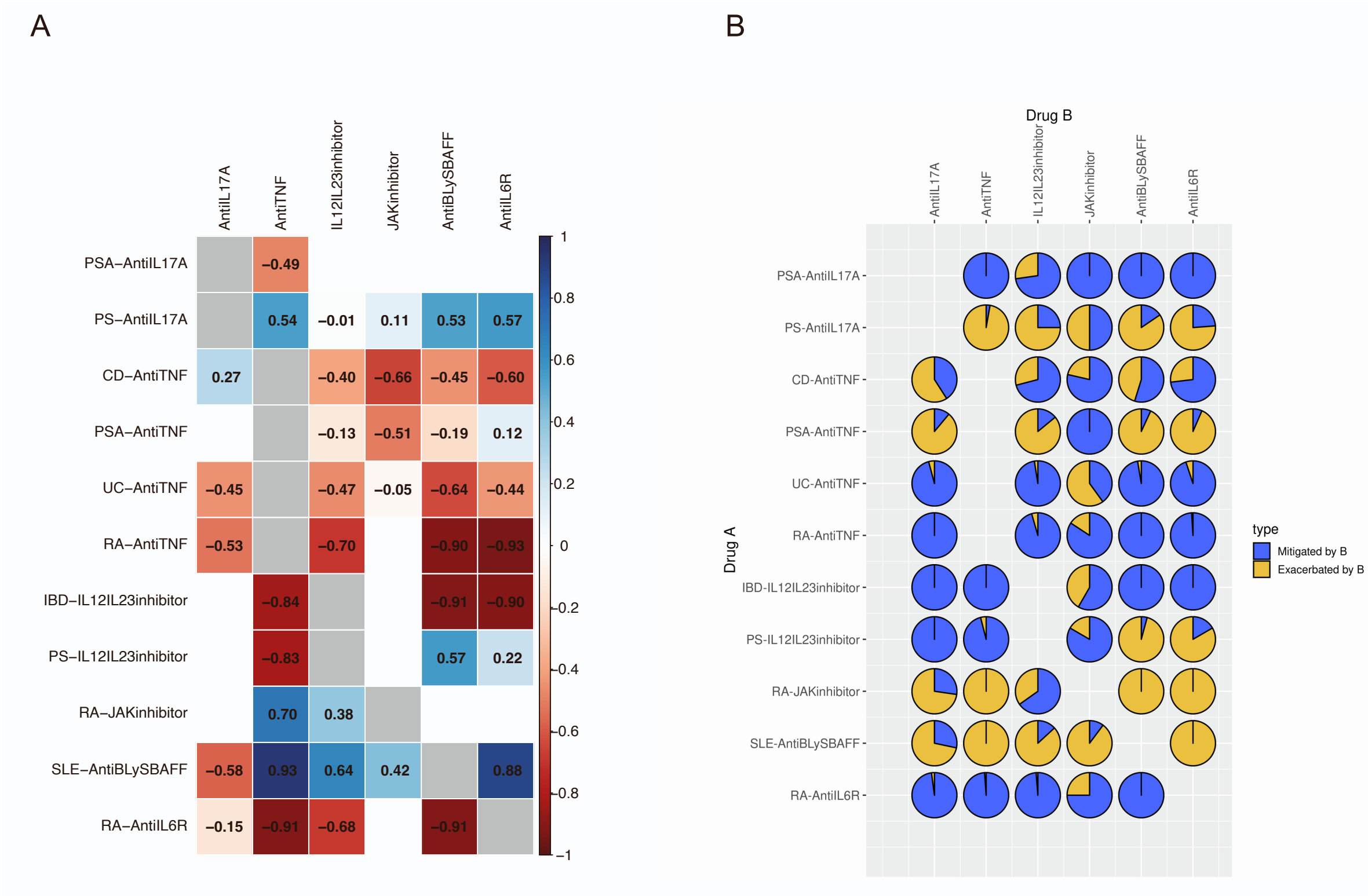

Figure S2. Mitigation of non-response (MNRS) analysis at the pathway level. (A) Pathway-level correlation coefficients between non-response signatures (rows) and drug effect signatures (columns). Pearson correlations were computed using only pathways significant at FDR<0.05 for both signatures. Correlations were not computed if there were less than 20 significant pathways (blank squares). (B) Non-response signature reversal percentages. Pathways associated to non-response (FDR<0.05) in the drug-disease cohort (rows) are categorized based on the longitudinal effect of drug B (columns). Antagonized by B: pathways that are reversed by drug B effect. Exacerbated by B: pathways that are exacerbated by drug B effect. Related to Figure 2.

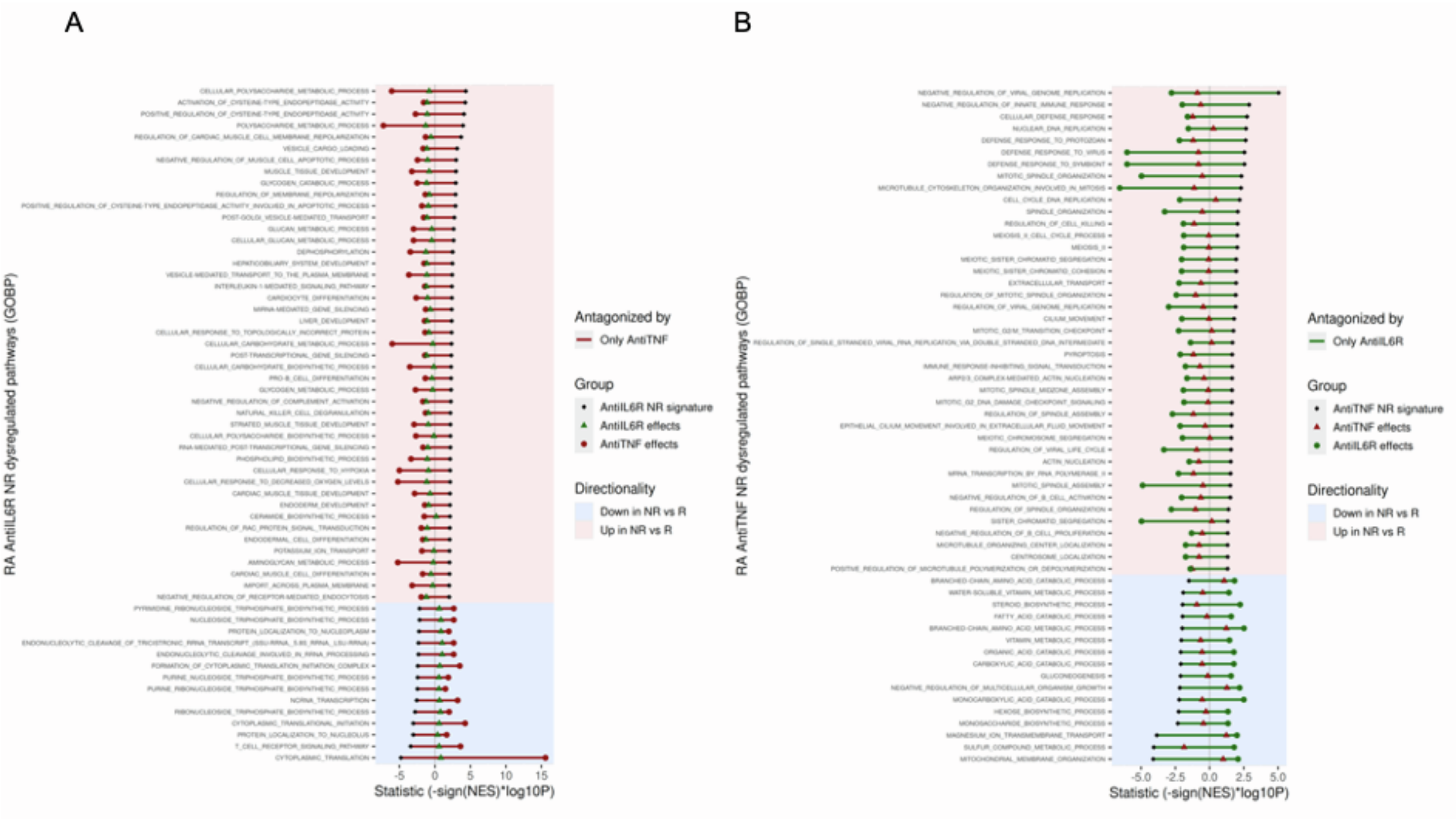

Figure S3. Pathway level characterization of the complementarity between anti-TNF and anti-IL6R in RA. Gene set enrichment analysis (GSEA) was performed using differential gene expression signatures as input. The enrichment statistic is defined shown as log10(P-value) of the enrichment test with the sign of the NES. (A) Pathways significantly associated to anti-IL6R non-response ($p<0.01$) that are significantly modulated by anti-TNF ($p<0.05$) but not by anti-IL6R ($p>0.05$). (B) Pathways significantly associated with anti-TNF non-response ($p<0.05$) that are significantly modulated by anti-IL6R ($p<0.05$) but not by anti-TNF ($p>0.05$). Black diamonds: pathways associated to non-response to therapies at baseline. Green dots/triangles: pathway association to anti-IL6R longitudinal effect. Red dots/triangles: pathway association to ant-TNF longitudinal effect. Related to Figure 2.

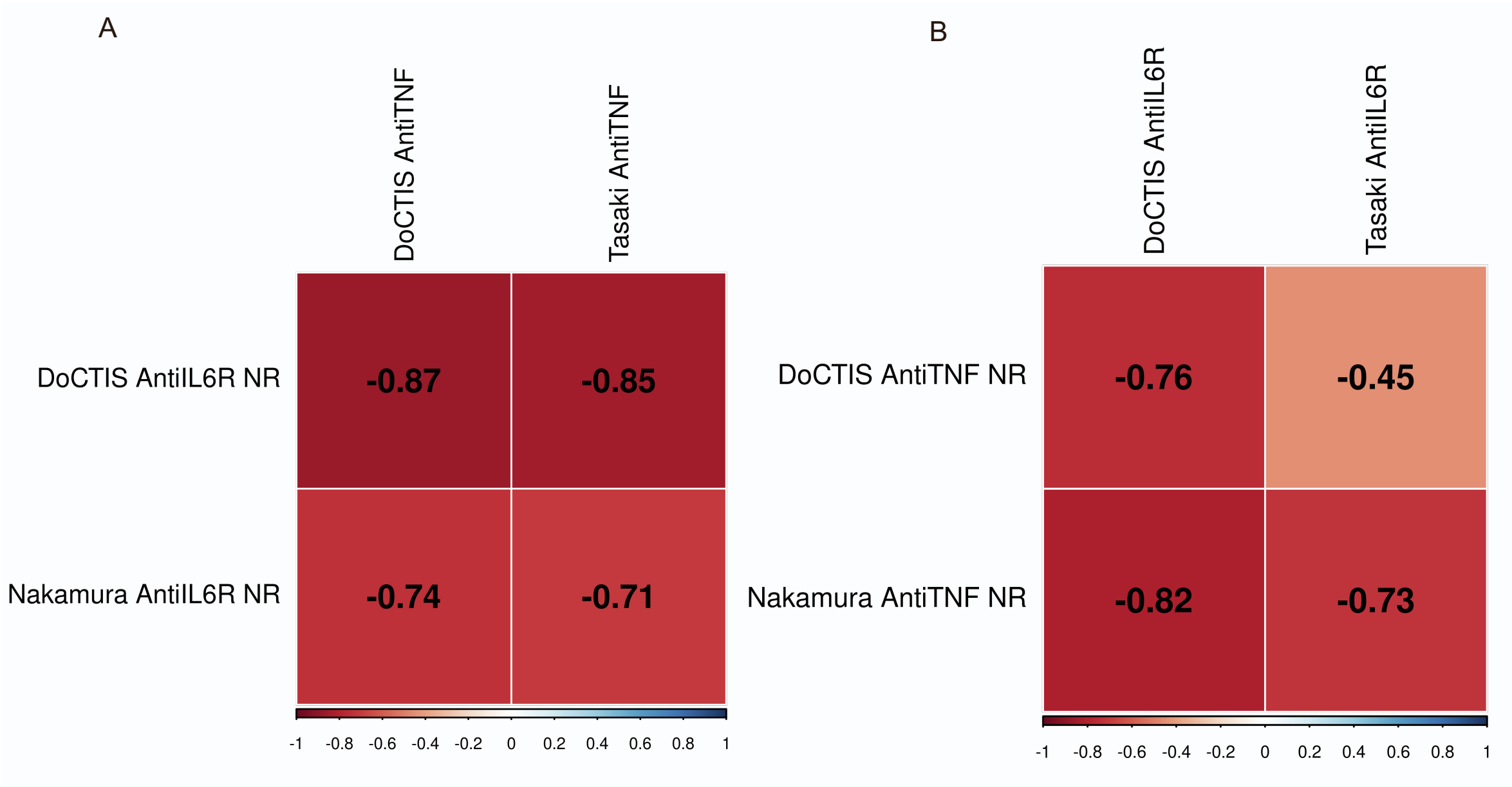

Figure S4. Replication of MNRS complementarity between anti-TNF and anti-IL6R combination in RA. Correlation coefficients between non-response signatures (rows) and drug effect signatures (columns). (A) Non-response signatures to anti-IL6R (rows) from Nakamura (GSE78068) and our study (DoCTIS) against the anti-TNF effect signatures (columns) from Tasaki (GSE93272), DoCTIS RA patients and DoCTIS multi-cohort analysis (global). (B) Non-response signatures (rows) to anti-TNF therapy from Nakamura (GSE78068) and DoCTIS against anti-IL6R treatment effect signatures (columns) from Tasaki (GSE93272) and DoCTIS RA patients. Related to Figure 2.

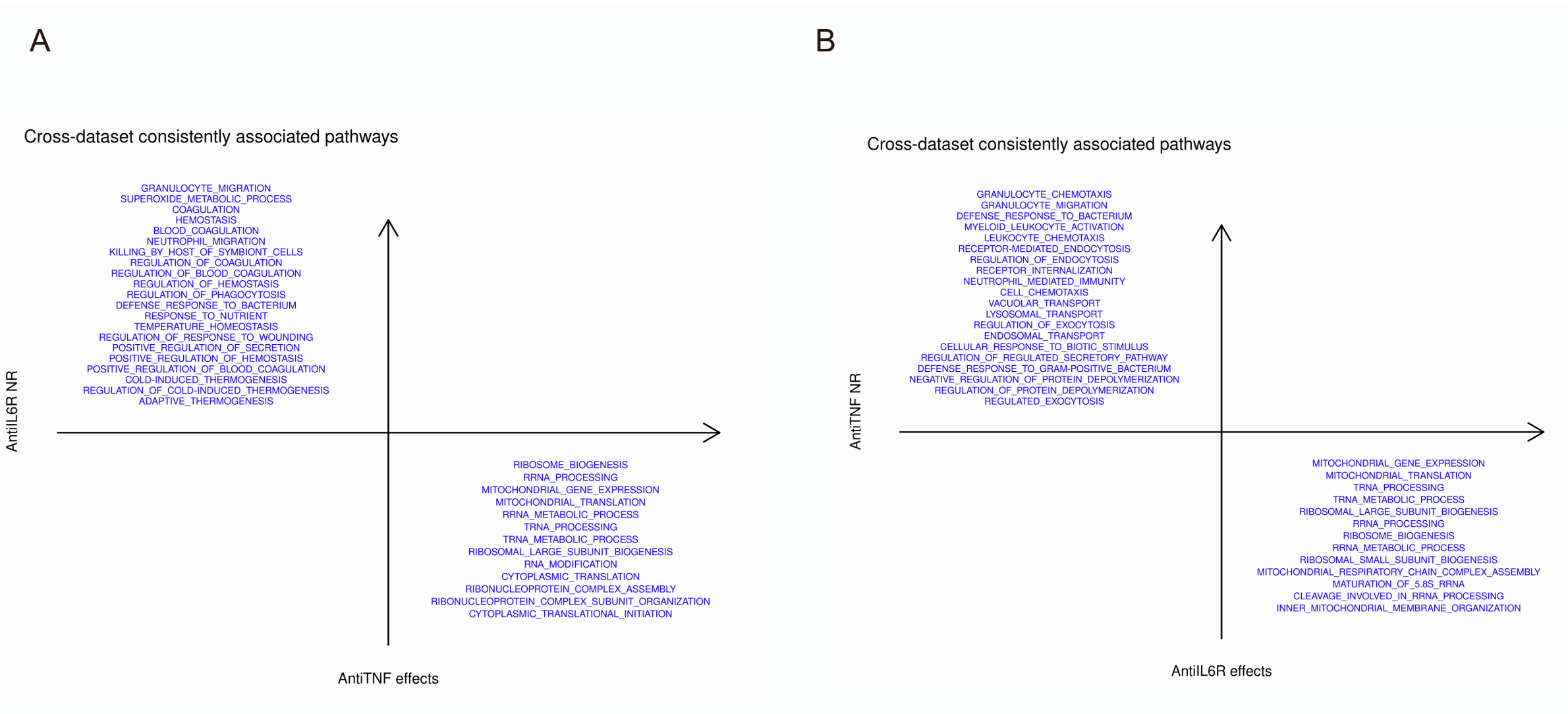

Figure S5. Pathway level analysis of the MNRS validation. Pathways consistently associated across datasets are displayed in four quadrants, based on the direction of their association. (A) Pathways that are consistently associated with anti-IL6R non-response in RA across Nakamura (GSE78068) and DoCTIS, and modulated by anti-TNF therapy in DoCTIS (global) and Tasaki (GSE93272). (B) Anti-TNF non-response signatures in RA from Nakamura (GSE78068) and DoCTIS compared to anti-IL6R treatment effects in DoCTIS RA patients and Tasaki (GSE93272). Related to Figure 2.

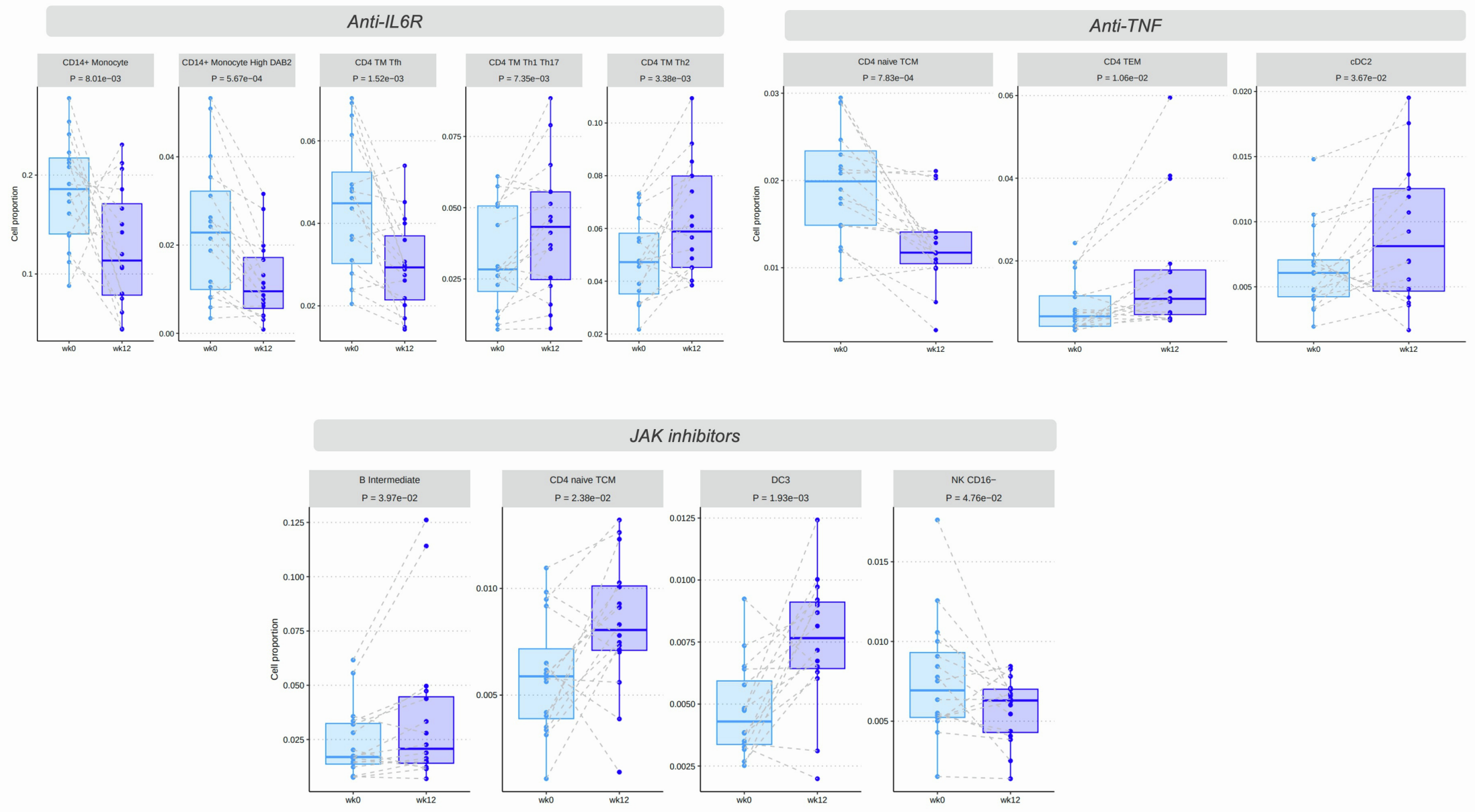

Figure S6. Proportion of cell types before and after therapy. Per each cohort, only cell types showing significant changes before and after therapy (P-value < 0.05) are represented. Related to Figure 3

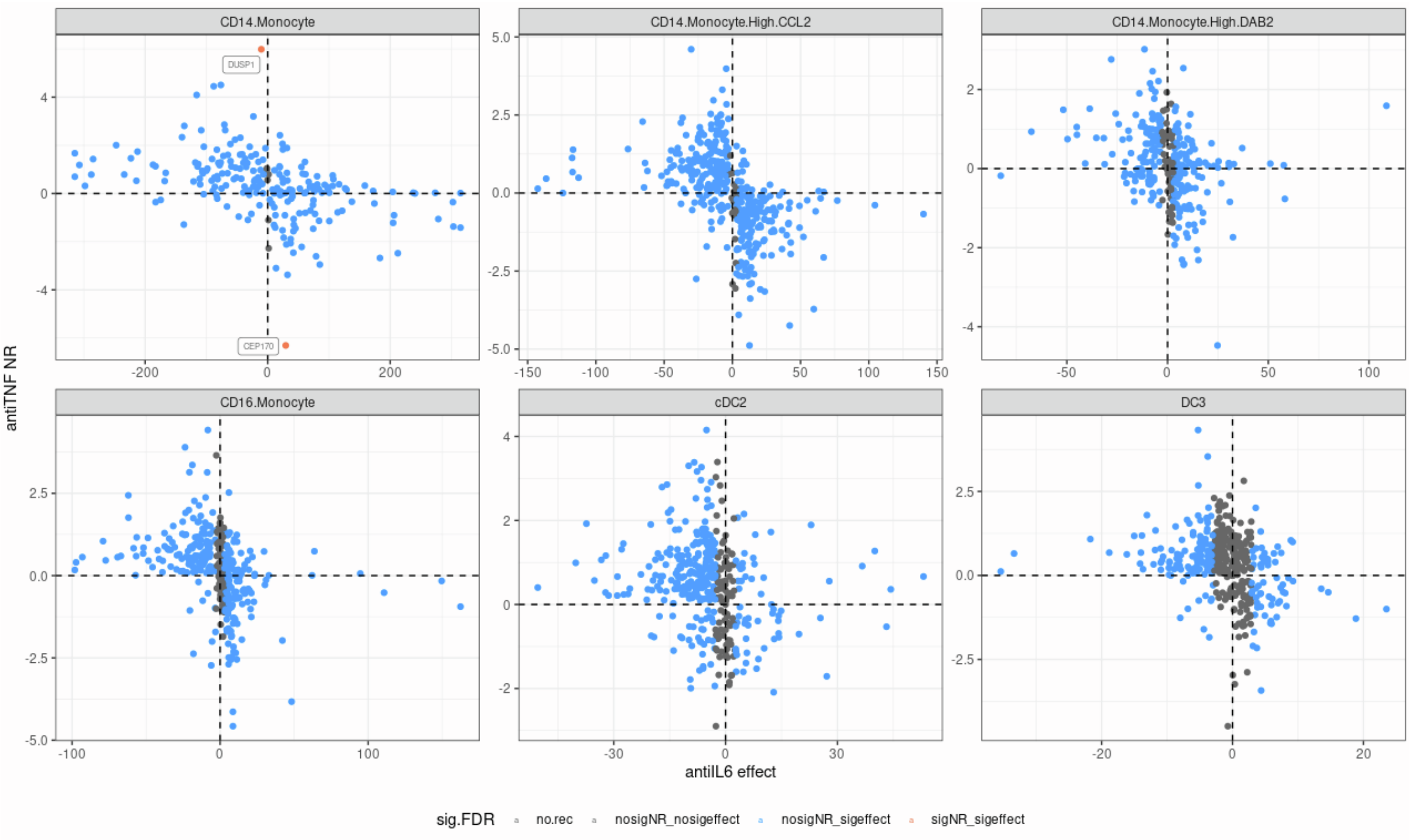

Figure S7. MNRS single cell analysis: anti-TNF non-response vs anti-IL6R longitudinal effect. X axis: anti-IL6R effect signature. Y axis: anti-TNF non-response signature. Values are shown as log10(P-value) of differential gene expression with the sign of the fold change. Only cell types in which the MNRS is significant are shown. Differentially expressed genes (P-value$_{adjusted}$ < 0.05) are labeled. Related to Figure 3.

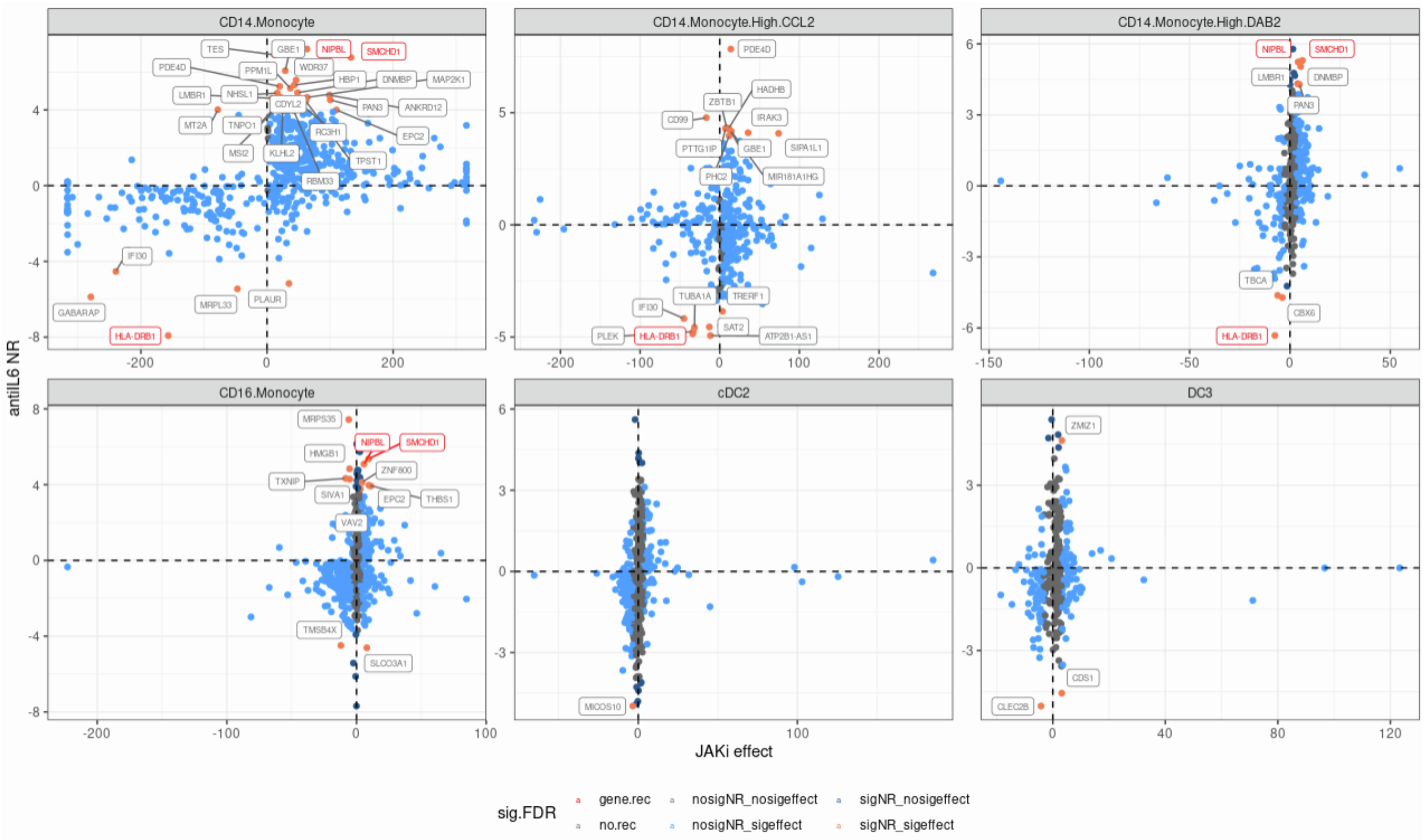

Figure S8. MNRS single cell analysis: anti-IL6R NR vs JAKi effect. X axis: JAKi effect signature. Y axis: anti-IL6R NR signature. Values are shown as log10(P-value) of differential gene expression with the sign of the fold change. Only cell types in which Fisher's exact test is significant are shown. Significantly differentially expressed genes (DEGs) (P-value adj < 0.05) are labeled. Genes differentially expressed in the maximum number of cell types are highlighted in red. Related to Figure 3.

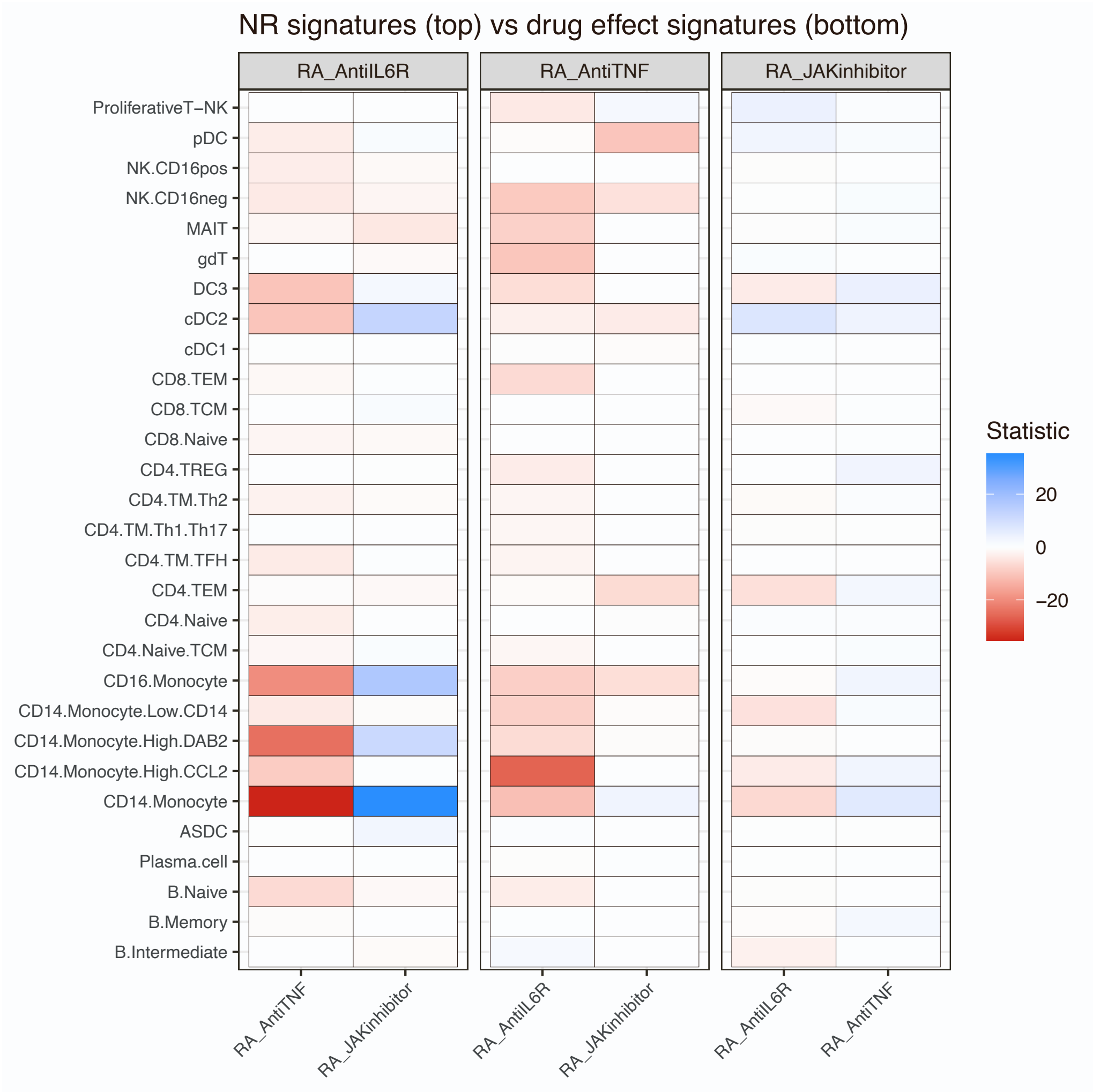

Figure S9. MNRS analysis per cell type on scRNAseq data from RA patients. Significant genes (P<0.05) were classified into four categories based on the directionality of the non-response and longitudinal effect association and the Fisher's test was performed to evaluate enrichment in the antagonization categories. The statistic is the logarithm of the P-value of the Fisher's exact test with sign given by the sign of the logarithm of the odds ratio. Non-response signatures are indicated on the top; effect signatures are indicated on the bottom. Related to Figure 3.

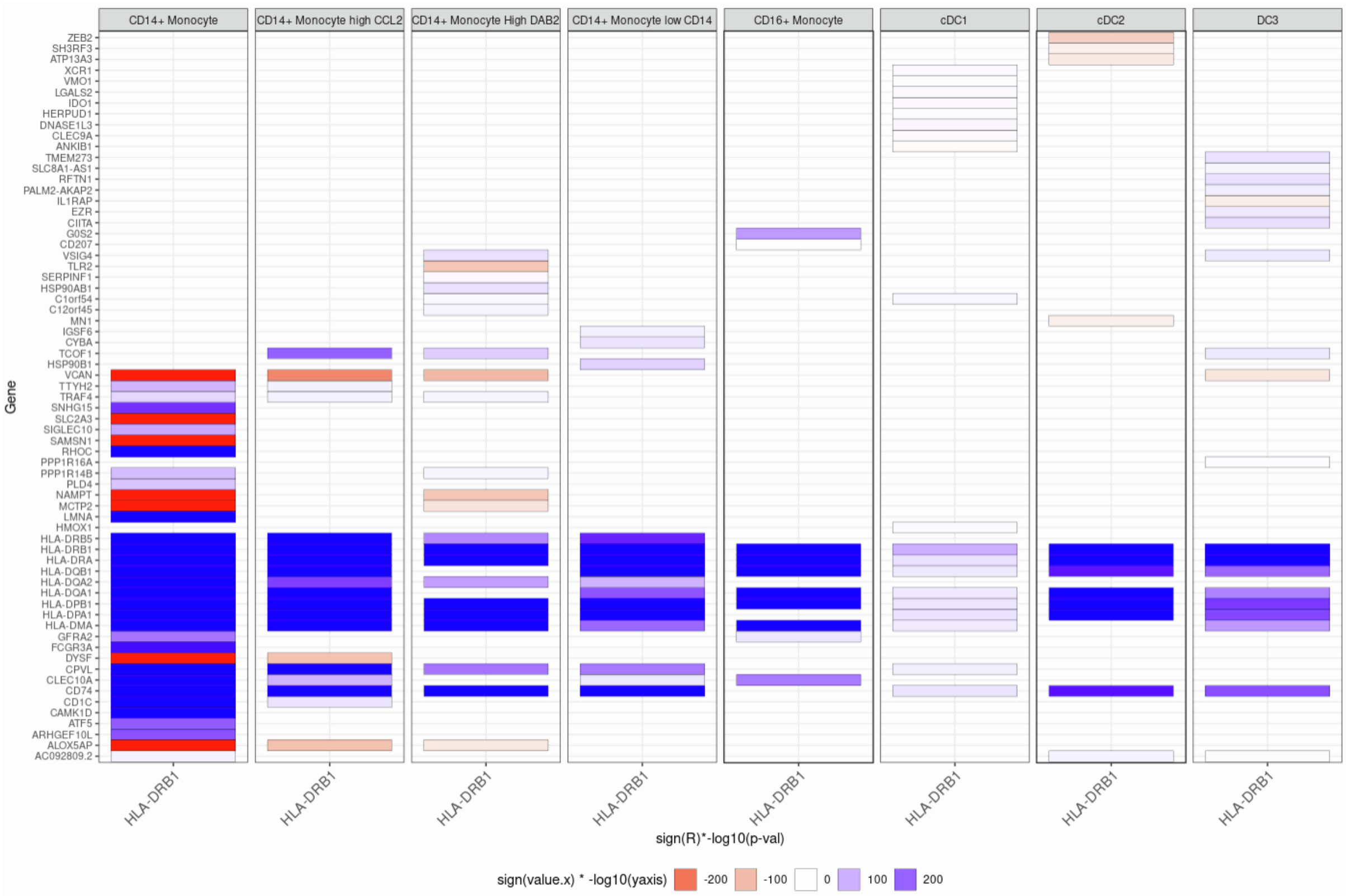

Figure S10. HLA-DRB1 co-expressed genes in myeloid cell subtypes. Gene modules were inferred using CSCORE. All the genes from the HLA-DRB1 module types are included. Significant correlations with HLA-DRB1 (P-value < 0.05, CSCORE estimate > 0.2 (blue) or <-0.2 (red) expression within each cell are shown. Related to Figure 4.

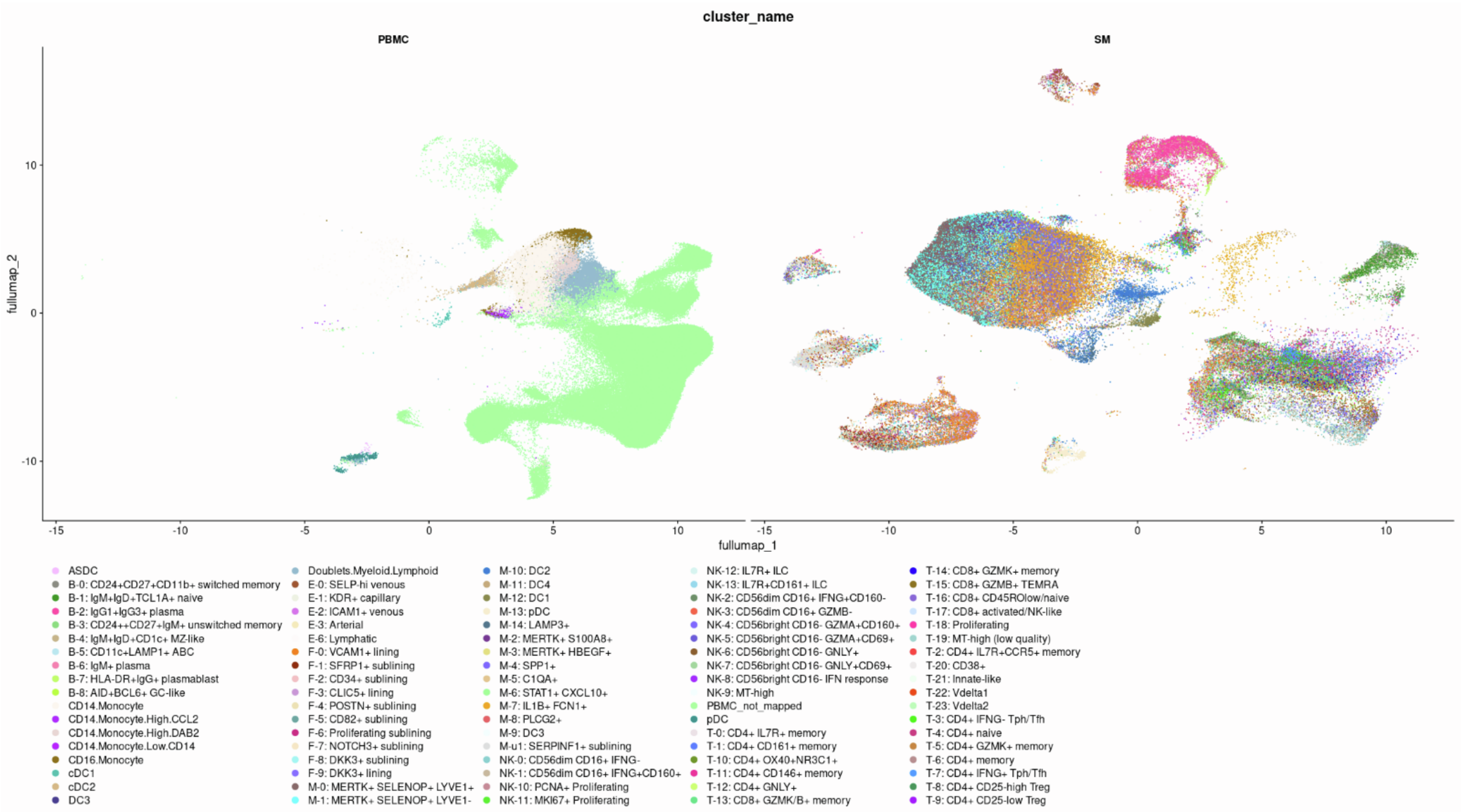

Figure S11. UMAP of scRNA-Seq data of paired PBMCs and synovial membrane samples. PBMCs are represented on the left panel, and synovial membrane cells on the right panel. Myeloid cells from PBMCs were annotated using our PBMC dataset. SM cells were mapped against the Accelerating Medicines Partnership atlas of the RA synovium [23]. Related to Figure 4.

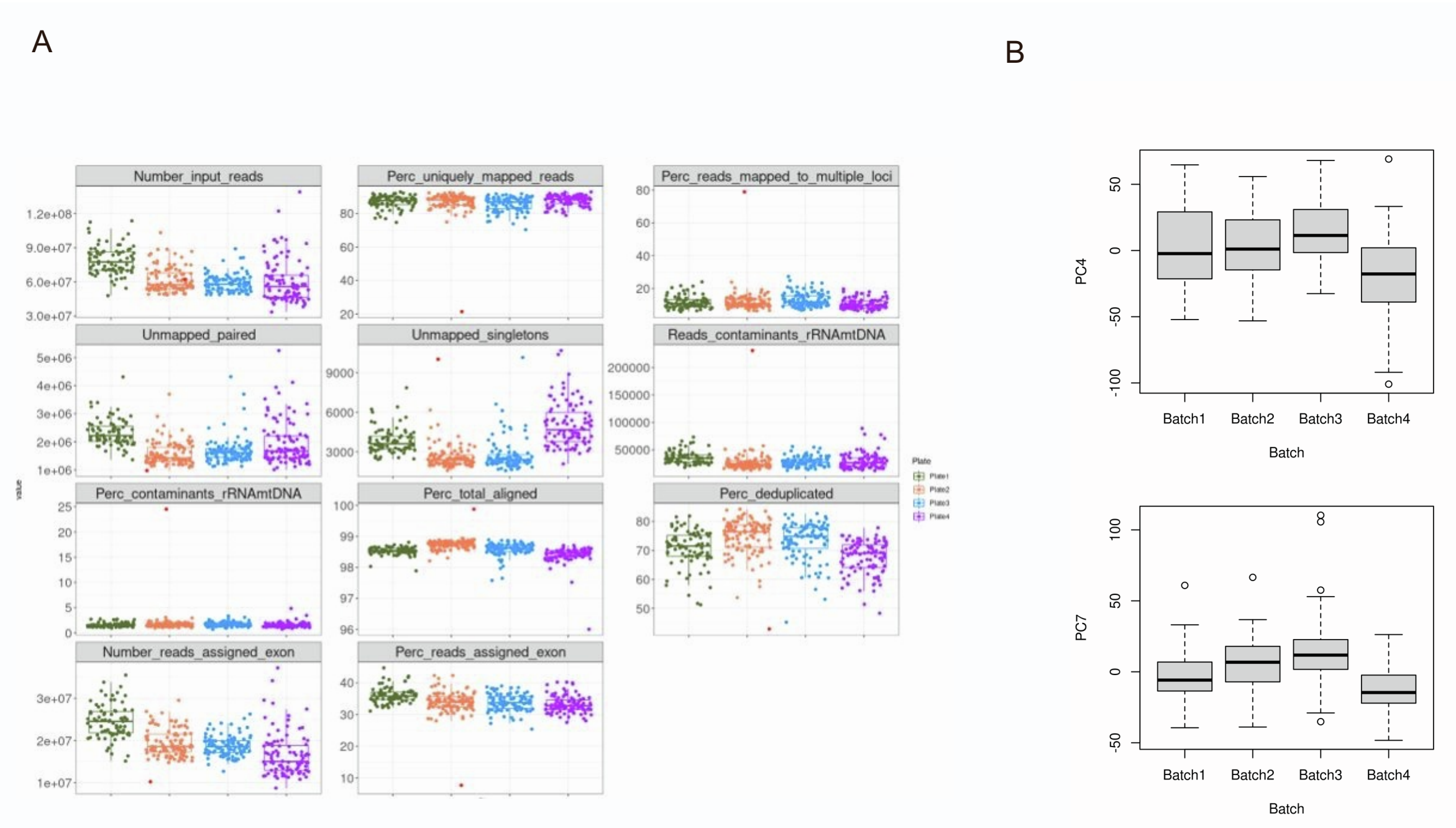

Figure S12. Bulk RNAseq quality control. (A) Association between bulk RNA-Seq parameters and sequencing batch (plate). *Number_input_reads*: Number of input filtered/trimmed reads. *Perc_uniquely_mapped_reads*: Percentage of reads mapped to unique regions of human genome. *Perc_reads_mapped_to_multiple_loci*: Percentage of reads mapping to multiple loci. *Unmapped_paired*: Paired reads that did not map to the human genome. *Unmapped_singletons*: Singleton sequences that did not map to the human genome. *Reads_contaminants_rRNAmtDNA*: Unmapped reads corresponding to rRNA or mtDNA. *Perc_contaminants_rRNAmtDNA*: Percentage of unmapped reads corresponding to rRNA or mtDNA. *Perc_total_aligned*: Sum of % uniquely and % multiple loci mapping reads. *Perc_deduplicated*: Percentage of reads deduplicated. *Number_reads_assigned_exon*: Number of reads assigned to annotated exons (featureCounts). *Perc_reads_assigned_exon*: Percentage of aligned reads mapped to annotated exons (featureCounts). One sample showed a high fraction of rRNA and mtDNA contamination as well as a low fraction of reads mapping to unique regions (red dot). This sample was successfully resequenced. (B) Principal components 4 (4% of explained variance) and 7 (2% of explained variance) of the global bulk gene expression matrix showed a significant association with the sequencing batch and were included as covariates in the differential expression analysis. Related to STAR Methods.

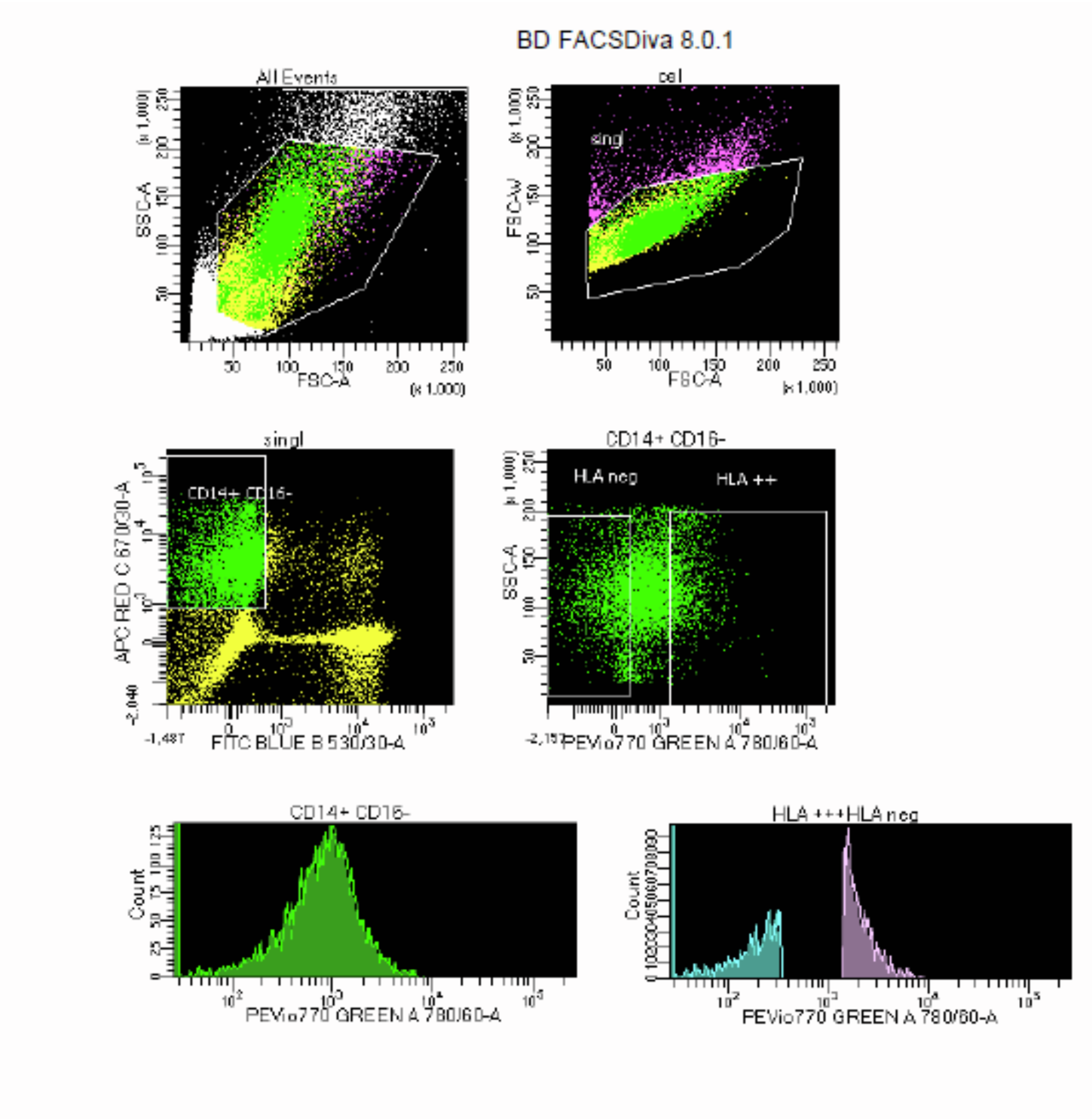

Figure S13. Monocyte-enriched cell suspensions obtained from fresh PBMCs were stained with anti-CD14-APC, anti-CD16-FITC and HLA-DRB1-PE-Vio. HLADRB1^{Hi} and HLADRB1-negative CD14+/CD16- were separated by cell sorting. Gates for separation according to HLADRB1 expression level are shown in the bottom right histogram; HLA-DRB1 negative and HLA++ (i.e. HLA-DRB1^{Hi}), colored as blue and violet, respectively. Related to STAR Methods.

SUPPLEMENTAL TABLES

Table S1. Baseline characteristics of the responders and non-responders from each disease-drug cohort. Related to Figure 1.

Table S1.A: PsA Anti-IL7A

	PSA Anti IL17A		
**Variable**	**R**<br>N = 8	**NR**<br>N = 8	**p-value**[1]
**Sex Female, n (%)**	6 (75%)	7 (88%)	>0.99
**Age, Mean (SD)**	49 (6)	55 (11)	0.43
**BMI, Mean (SD)**	24.0 (4.8)	27.7 (5.6)	0.16
**Cigarette Frequency, n (%)**			>0.99
Daily	2 (25%)	1 (13%)	
Occasionally	0 (0%)	0 (0%)	
Never	6 (75%)	7 (88%)	
**Disease Duration Years, Mean (SD)**	9 (8)	18 (11)	0.083
**Days To Follow-up, Mean (SD)**	94 (14)	86 (6)	0.063
**Concomitant GCs, n (%)**	4 (50%)	6 (75%)	0.61
**Baseline Activity, Median (Q1, Q3)**	4.99 (4.89, 5.64)	4.66 (4.11, 5.11)	0.16

[1]Fisher's exact test; Wilcoxon rank sum test; Wilcoxon rank sum exact test

Table S1.B: SLE Anti-BlyS/BAFF

	SLE Anti BLyS/BAFF		
**Variable**	**R**<br>N = 8	**NR**<br>N = 8	**p-value**[1]
**Sex Female, n (%)**	8 (100%)	8 (100%)	
**Age, Mean (SD)**	35 (13)	44 (13)	0.25
**BMI, Mean (SD)**	27.0 (6.0)	23.3 (3.9)	0.25
**Cigarette Frequency, n (%)**			0.47
Daily	2 (25%)	0 (0%)	
Occasionally	0 (0%)	0 (0%)	
Never	6 (75%)	8 (100%)	
**Disease Duration Years, Mean (SD)**	9 (9)	11 (7)	0.46
**Days To Follow-up, Mean (SD)**	84 (12)	104 (27)	0.14
**Concomitant GCs, n (%)**	6 (75%)	7 (88%)	>0.99
**Baseline Activity, Median (Q1, Q3)**	9.50 (8.00, 10.50)	7.00 (4.50, 8.50)	0.090

[1]NA; Wilcoxon rank sum test; Fisher's exact test

Table S1.C: RA Anti-IL6R

	RA Anti IL6R		
**Variable**	**R**<br>N = 8	**NR**<br>N = 8	**p-value**[1]
**Sex Female, n (%)**	8 (100%)	6 (75%)	0.47
**Age, Mean (SD)**	55 (12)	55 (11)	>0.99
**BMI, Mean (SD)**	24.5 (10.7)	28.4 (6.3)	0.65
**Cigarette Frequency, n (%)**			0.46
Daily	0 (0%)	2 (29%)	
Occasionally	0 (0%)	0 (0%)	
Never	7 (100%)	5 (71%)	
**Disease Duration Years, Mean (SD)**	11 (6)	14 (12)	>0.99
**ACPA+, n (%)**	6 (75%)	8 (100%)	0.47
**RF+, n (%)**	7 (88%)	8 (100%)	>0.99
**Erosive, n (%)**	0 (0%)	0 (0%)	
**Days To Follow-up, Mean (SD)**	87 (15)	89 (12)	0.92
**Concomitant GCs, n (%)**	6 (75%)	6 (75%)	>0.99

Variable	RA Anti IL6R<br>R<br>N = 8	 NR<br>N = 8	 p-value[1]
**Concomitant MTX, n (%)**	3 (38%)	3 (38%)	>0.99
**Baseline Activity, Median (Q1, Q3)**	5.45 (4.63, 6.28)	5.56 (4.52, 6.02)	>0.99

[1]Fisher's exact test; Wilcoxon rank sum test; Wilcoxon rank sum exact test; NA

Table S1.D: UC Anti-TNF

Variable	UC Anti TNF<br>R<br>N = 8	 NR<br>N = 8	 p-value[1]
**Sex Female, n (%)**	4 (50%)	4 (50%)	>0.99
**Age, Mean (SD)**	39 (15)	46 (19)	0.34
**BMI, Mean (SD)**	22.7 (3.5)	26.3 (6.2)	0.23
**Cigarette Frequency, n (%)**			>0.99
Daily	2 (25%)	1 (13%)	
Occasionally	0 (0%)	1 (13%)	
Never	6 (75%)	6 (75%)	
**Disease Duration Years, Mean (SD)**	6.0 (2.8)	7.0 (7.8)	0.84
**Days To Follow-up, Mean (SD)**	99.1 (2.8)	98.6 (8.6)	0.18
**Concomitant GCs, n (%)**	2 (25%)	2 (25%)	>0.99
**Baseline Activity, Median (Q1, Q3)**	6.50 (5.50, 7.50)	8.00 (4.50, 9.50)	0.63

[1]Fisher's exact test; Wilcoxon rank sum test; Wilcoxon rank sum exact test

Table S1.E: PsA Anti-TNF

Variable	PSA Anti TNF<br>R<br>N = 8	 NR<br>N = 8	 p-value[1]
**Sex Female, n (%)**	4 (50%)	6 (75%)	0.61
**Age, Mean (SD)**	50 (11)	49 (12)	>0.99
**BMI, Mean (SD)**	27.0 (2.8)	24.8 (4.8)	0.54
**Cigarette Frequency, n (%)**			>0.99
Daily	1 (13%)	1 (13%)	
Occasionally	1 (13%)	0 (0%)	
Never	6 (75%)	7 (88%)	
**Disease Duration Years, Mean (SD)**	8.3 (5.7)	3.1 (2.8)	0.052
**Days To Follow-up, Mean (SD)**	85 (9)	88 (9)	>0.99
**Concomitant GCs, n (%)**	3 (38%)	4 (50%)	>0.99
**Baseline Activity, Median (Q1, Q3)**	4.29 (4.04, 4.68)	4.59 (3.40, 5.65)	0.96

[1]Fisher's exact test; Wilcoxon rank sum test; Wilcoxon rank sum exact test

Table S1.F: PS IL12IL23 inhibitor

Variable	PS IL12IL23 inhibitor<br>R<br>N = 8	 NR<br>N = 8	 p-value[1]
**Sex Female, n (%)**	4 (50%)	2 (25%)	0.61
**Age, Mean (SD)**	45 (10)	48 (12)	0.49
**BMI, Mean (SD)**	25.3 (3.8)	28.5 (3.3)	0.10
**Cigarette Frequency, n (%)**			0.20
Daily	3 (38%)	0 (0%)	
Occasionally	0 (0%)	0 (0%)	
Never	5 (63%)	8 (100%)	

	PS IL12IL23 inhibitor		
**Variable**	**R**<br>N = 8	**NR**<br>N = 8	**p-value**[1]
**Disease Duration Years, Mean (SD)**	22 (12)	17 (8)	0.67
**Days To Follow-up, Mean (SD)**	103 (21)	106 (13)	0.92
**Concomitant GCs, n (%)**	0 (0%)	0 (0%)	
**Baseline Activity, Median (Q1, Q3)**	21 (13, 24)	13 (11, 21)	0.46

[1]Fisher's exact test; Wilcoxon rank sum test; Wilcoxon rank sum exact test; NA

Table S1.G: CD Anti TNF

	CD Anti TNF		
**Variable**	**R**<br>N = 8	**NR**<br>N = 8	**p-value**[1]
**Sex Female, n (%)**	5 (63%)	5 (63%)	>0.99
**Age, Mean (SD)**	44 (16)	46 (10)	0.79
**BMI, Mean (SD)**	25.0 (7.4)	23.7 (3.2)	>0.99
**Cigarette Frequency, n (%)**			>0.99
Daily	4 (50%)	4 (50%)	
Occasionally	2 (25%)	1 (13%)	
Never	2 (25%)	3 (38%)	
**Disease Duration Years, Mean (SD)**	7 (7)	11 (11)	0.52
**Days To Follow-up, Mean (SD)**	103 (12)	104 (9)	0.67
**Concomitant GCs, n (%)**	4 (50%)	2 (25%)	0.61
**Baseline Activity, Median (Q1, Q3)**	191 (158, 217)	148 (86, 170)	0.13

[1]Fisher's exact test; Wilcoxon rank sum test; Wilcoxon rank sum exact test

Table S1.H: RA JAK inhibitor

	RA JAK inhibitor		
**Variable**	**R**<br>N = 8	**NR**<br>N = 8	**p-value**[1]
**Sex Female, n (%)**	7 (88%)	8 (100%)	>0.99
**Age, Mean (SD)**	54 (12)	44 (15)	0.10
**BMI, Mean (SD)**	25.4 (5.1)	26.4 (6.2)	0.72
**Cigarette Frequency, n (%)**			>0.99
Daily	1 (13%)	2 (25%)	
Occasionally	0 (0%)	0 (0%)	
Never	7 (88%)	6 (75%)	
**Disease Duration Years, Mean (SD)**	10 (11)	13 (9)	0.37
**ACPA+, n (%)**	7 (100%)	8 (100%)	
**RF+, n (%)**	8 (100%)	8 (100%)	
**Erosive, n (%)**	0 (0%)	0 (0%)	
**Days To Follow-up, Mean (SD)**	90 (11)	84 (7)	0.30
**Concomitant GCs, n (%)**	5 (63%)	7 (88%)	0.57
**Concomitant MTX, n (%)**	1 (13%)	3 (38%)	0.57
**Baseline Activity, Median (Q1, Q3)**	5.50 (5.20, 5.84)	6.35 (4.91, 6.85)	0.37

[1]Fisher's exact test; Wilcoxon rank sum test; Wilcoxon rank sum exact test; NA

Table S1.I: RA Anti TNF

	RA Anti TNF		
**Variable**	**R**<br>N = 8	**NR**<br>N = 8	**p-value**[1]
**Sex Female, n (%)**	8 (100%)	8 (100%)	

RA Anti TNF			
**Variable**	**R**<br>N = 8	**NR**<br>N = 8	**p-value**[1]
**Age, Mean (SD)**	48 (16)	51 (11)	0.64
**BMI, Mean (SD)**	25.3 (4.6)	27.9 (5.3)	0.37
**Cigarette Frequency, n (%)**			>0.99
Daily	2 (25%)	3 (38%)	
Occasionally	0 (0%)	0 (0%)	
Never	6 (75%)	5 (63%)	
**Disease Duration Years, Mean (SD)**	7.8 (7.0)	8.1 (5.6)	0.87
**ACPA+, n (%)**	8 (100%)	8 (100%)	
**RF+, n (%)**	8 (100%)	7 (88%)	>0.99
**Erosive, n (%)**	0 (0%)	0 (0%)	
**Days To Follow-up, Mean (SD)**	89 (13)	88 (17)	0.83
**Concomitant GCs, n (%)**	8 (100%)	8 (100%)	
**Concomitant MTX, n (%)**	5 (63%)	2 (25%)	0.31
**Baseline Activity, Median (Q1, Q3)**	4.56 (4.08, 5.74)	5.07 (3.82, 6.54)	>0.99

[1]NA; Wilcoxon rank sum test; Fisher's exact test

Table S1.J: PS Anti IL17A

PS Anti IL17A			
**Variable**	**R**<br>N = 11	**NR**<br>N = 5	**p-value**[1]
**Sex Female, n (%)**	5 (45%)	4 (80%)	0.31
**Age, Mean (SD)**	53 (14)	58 (12)	>0.99
**BMI, Mean (SD)**	28.7 (5.0)	30.0 (5.0)	0.83
**Cigarette Frequency, n (%)**			0.59
Daily	2 (18%)	2 (40%)	
Occasionally	2 (18%)	0 (0%)	
Never	7 (64%)	3 (60%)	
**Disease Duration Years, Mean (SD)**	30 (21)	13 (10)	0.15
**Days To Follow-up, Mean (SD)**	96 (16)	90 (18)	0.58
**Concomitant GCs, n (%)**	0 (0%)	0 (0%)	
**Baseline Activity, Median (Q1, Q3)**	8.00 (6.00, 10.20)	6.90 (5.30, 7.50)	0.46

[1]Fisher's exact test; Wilcoxon rank sum test; Wilcoxon rank sum exact test; NA

Table S1.K: IBD IL12IL23 inhibitor

IBD IL12IL23 inhibitor			
**Variable**	**R**<br>N = 7	**NR**<br>N = 9	**p-value**[1]
**Sex Female, n (%)**	4 (57%)	4 (44%)	>0.99
**Age, Mean (SD)**	48 (10)	51 (19)	0.79
**BMI, Mean (SD)**	25.5 (5.8)	25.1 (3.6)	0.87
**Cigarette Frequency, n (%)**			>0.99
Daily	0 (0%)	0 (0%)	
Occasionally	0 (0%)	1 (11%)	
Never	7 (100%)	8 (89%)	
**Disease Duration Years, Mean (SD)**	6 (4)	15 (15)	0.24
**Days To Follow-up, Mean (SD)**	103 (11)	104 (7)	>0.99
**Concomitant GCs, n (%)**	1 (14%)	1 (11%)	>0.99
**Baseline Activity, Median (Q1, Q3)**	148 (7, 216)	126 (9, 193)	0.71

[1]Fisher's exact test; Wilcoxon rank sum test; Wilcoxon rank sum exact test

Table S7. Sensitivity analysis results for the MNRS correlation between Anti-TNF and Anti-IL6R. Concomitant OGC = Concomitant treatment with oral glucocorticoids. ConcomitantMTX = Concomitant treatment with Methotrexate. Related to Figure 2.

NR Sig. Adjustment	AntiIL6R NR - AntiTNF Effect r (95%CI)	AntiTNF NR - AntiIL6R Effect r (95%CI)
Default	-0.87 (-0.88, -0.86)	-0.76 (-0.79, -0.72)
SexAge	-0.87 (-0.88, -0.86)	-0.79 (-0.82, -0.77)
ConcomitantMTX	-0.86 (-0.87, -0.85)	-0.52 (-0.59, -0.45)
DaysToFollowup	-0.87 (-0.88, -0.86)	-0.74 (-0.78, -0.71)
ConcomitantOGC	-0.87 (-0.88, -0.86)	-
Disease DurationYears	-0.87 (-0.88, -0.86)	-0.77 (-0.8, -0.73)

Table S13. Clinical information of patients used for the in vitro characterization of anti-TNF effect in PBMCs (Parse single-cell RNASeq). Concomitant GCs = Concomitant treatment with oral glucocorticoids. Related to Figure 5.

	**RA**
**SexFemale, n (%)**	6 (100%)
**Age, Mean (SD)**	52.8 (13.4)
**BMI, Mean (SD)**	24.31 (4.8)
**ACPA positive, n (%)**	6 (100%)
**RF positive, n (%)**	5 (83.3%)
**Cigarette Frequency, n (%)**	
Daily	2 (33.3%)
Occasionally	0 (0%)
Never	4 (66.7%)
**Disease Duration Years, Mean (SD)**	4.6 (3.8)
**Concomitant GCs, n (%)**	6 (100%)
**Disease Activity, Median (Q1, Q3)**	4.65 (4.47, 7.60)

SUPPLEMENTAL INFORMATION

Data S1. Detailed patient eligibility and response criteria

For all cohorts, general patient requirements included having:

- Body Mass Index < 36
- Standardized diagnosis of the corresponding IMID and no other IMID or severe comorbidities such as cancer, cardiovascular disease or diabetes
- No more than one failure to previous biological therapies

For RA-AntiIL6R:

- Considered drugs: Tocilizumab, Sarilumab
- Minimum disease activity score at baseline: DAS28=3.2
- Response criteria: responders were good responders by the EULAR criteria , non-responders were non-responders by the EULAR criteria
- Patients were not considered if they had been previously treated with Rituximab

For RA-AntiTNF:

- Considered drugs: Adalimumab, Certolizumab, Etanercept and Golimumab
- Minimum disease activity score at baseline: DAS28=3.2
- Response criteria: responders were good responders by the EULAR criteria [61], non-responders were non-responders by the EULAR criteria
- Patients were not considered if they had been previously treated with Rituximab, or if they were negative for both rheumatoid factor and ACPA

For RA-JAKi:

- Considered drugs: Tofacitinib, Baricitinib
- Minimum disease activity score at baseline: DAS28=3.2
- Response criteria: responders were good responders by the EULAR criteria [61], non-responders were non-responders by the EULAR criteria
- Patients were not considered if they had been previously treated with Rituximab, or if they were negative for both rheumatoid factor and ACPA

For PsA-AntiTNF:

- Considered drugs: Adalimumab, Certolizumab, Etanercept, Golimumab, Infliximab
- Minimum disease activity score at baseline: DAS28=3.2
- Response criteria: responders were good responders by the EULAR criteria [61], non-responders were non-responders by the EULAR criteria
- Patients were not considered if they had been previously treated with Abatacept

For PsA-AntiIL17A:

- Considered drugs: Secukinumab, Ixekizumab
- Minimum disease activity score at baseline: DAS28=3.2
- Response criteria: responders were good responders by the EULAR criteria [61], non-responders were non-responders by the EULAR criteria
- Patients were not considered if they had been previously treated with abatacept

For PS-AntiIL12/23:

- Considered drugs: Ustekinumab
- Minimum disease activity score at baseline: PASI = 8
- Response criteria: responders met the PASI90 criteria [62], non-responders did not meet PASI75 criteria.

For PS-AntiIL17:

- Considered drugs: Brodalumab, Ixekizumab, Secukinumab
- Response criteria: responders met the PASI75 criteria [62], non-responders did not meet PASI75 criteria.

For CD-AntiTNF:

- Considered drugs: Infliximab, Adalimumab
- Patients should have an inflammatory disease behaviour, and have colonic or ileo-colonic disease location
- Minimum disease activity score at baseline: CDAI [63] of 50
- Response criteria: responders had a decrease in CDAI of at least 70 points and at least 25% in subjects with baseline score greater than or equal to 220

For UC-AntiTNF:

- Considered drugs: Infliximab, Adalimumab
- Minimum disease activity score at baseline: MAYO score [64] of 3
- Response criteria: responders needed to have a decrease of at least 2 points in the MAYO clinical score with MAYO stools score of 0 and global score less than or equal to 1 at follow-up

For IBD-AntiIL12/23:

- Considered drugs: Ustekinumab
- Diagnosis of Crohn's disease or Ulcerative Colitis
- Minimum disease activity score at baseline: MAYO score [64] of 3 for UC and CDAI [63] of 50 for CD
- Response criteria: CD responders had a decrease in CDAI of at least 70 points and at least 25% in subjects with baseline score greater than or equal to 220. UC responders needed to have a decrease of at least 2 points in the MAYO clinical score with MAYO stools score of 0 and global score less than or equal to 1 at follow-up

For SLE-AntiBLyS/BAFF:

- Considered drugs: Belimumab
- Minimum disease activity score at baseline: SELENA-SLEDAI [65] of 4
- Response criteria: responders met the SRI4 criteria [66], non-responders needed to have an improvement less than or equal to 2 points in SELENA-SLEDAI
- Males were excluded

List of participants of the IMID-Consortium

MªAngeles Aguirre Zamorano; Rheumatology Department, Hospital Universitario Reina Sofia; Córdoba, Spain.

Mercedes Alperiz; Rheumatology Department, Hospital Universitario Central de Asturias; Asturias, Spain.

José Luís Andreu; Rheumatology Department, Hospital Universitario Puerta de Hierro; Madrid, Spain.

Manuel Barreiro; Gastroenterology Department, University Hospital of Santiago de Compostela; Santiago de Compostela, Spain.

Isabel Belinchón; Dermatology Department, Hospital General Universitario de Alicante; Alicante, Spain.

Diego Benavente; Rheumatology Department, Hospital Universitari de Bellvitge; Barcelona, Spain.

Francisco Blanco; Rheumatology Department, Complejo Hospitalario Universitario de A Coruña; A Coruña, Spain.

Sara Blanco; Dermatology Department, Hospital Universitario de Salamanca; Salamanca, Spain.

Ricardo Blanco; Rheumatology Department, Hospital Universitario Marqués de Valdecilla; Santander, Spain.

Natalia Borruel; Gastroenterology Department, Hospital Universitari Vall d'Hebron; Barcelona, Spain.

Luis Bujanda; Gastroenterology Department, Hospital Universitario de Donostia; San Sebastián, Spain.

José Luís Cabriada; Gastroenterology Department, Hospital Universitario de Galdakao; Bilbao, Spain.

Jaime Calvo Alén; Rheumatology Department, Hospital Universitario Araaba; Alava, Spain.

Juan Cañete; Rheumatology Department, Fundació Clínic per a la Recerca Biomèdica; Barcelona, Spain.

Jose Manuel Carrascosa; Dermatology Department, Hospital Universitari Germans Trias i Pujol; Badalona, Spain.

Patricia Carreira; Rheumatology Department, Hospital Universitario 12 de Octubre; Madrid, Spain.

Santos Castañeda; Rheumatology Department, Hospital Universitario La Princesa; Madrid, Spain.

Hèctor Corominas; Rheumatology Department, Hospital de la Santa Creu i Sant Pau; Barcelona, Spain.

Josefina Cortés; Rheumatology Department, Hospital Universitari Vall d'Hebron; Barcelona, Spain.

Pablo de la Cueva; Dermatology Department, Hospital Universitario Infanta Leonor; Madrid, Spain.

Esteban Dauden; Dermatology Department, Hospital Universitario La Princesa; Madrid, Spain.

César Díaz; Rheumatology Department, Hospital de la Santa Creu i Sant Pau; Barcelona, Spain.

Eugeni Domènech; Gastroenterology Department, Hospital Universitari Germans Trias i Pujol; Badalona, Spain.

Elvira Díez; Rheumatology Department, Complejo Asistencial Universitario de León; León, Spain.

Alba Erra; Rheumatology Department, Hospital Sant Rafael; Barcelona, Spain.

Maria Esteve Comas; Gastroenterology Department, Hospital Universitari Mútua de Terrasa; Terrassa, Spain.

Benjamín Fernández; Rheumatology Department, Hospital Clínico San Carlos; Madrid, Spain.

Antonio Fernández-Nebro; Rheumatology Department, Hospital Regional Universitario Carlos Haya; Málaga, Spain.

Eduardo Fonseca; Dermatology Department, Complejo Hospitalario Universitario de A Coruña; A Coruña, Spain.

Mercedes Freire; Rheumatology Department, Complejo Hospitalario Universitario de A Coruña; A Coruña, Spain.

Esther García-Planella; Gastroenterology Department, Hospital de la Santa Creu i Sant Pau; Barcelona, Spain.

Vicenç García Patos; Dermatology Department, Hospital Universitari Vall d'Hebron; Barcelona, Spain.

Charo García de Vicuña; Rheumatology Department, Hospital Universitario La Princesa; Madrid, Spain.

María Jesús García Villanueva; Rheumatology Department, Hospital Universitario Ramón y Cajal

María Luz García Vivar; Rheumatology Department, Hospital Universitario de Basurto; Bilbao, Spain.

Fernando Gomollón García; Gastroenterology Department, Hospital Clínico Universitario Lozano Blesa; Zaragoza, Spain.

Carlos González; Rheumatology Department, Hospital Universitario Gregorio Marañón; Madrid, Spain.

Maria José González; Rheumatology Department, Hospital Universitario Quiron Dexeus; Barcelona, Spain.

Isidoro González; Rheumatology Department, Hospital Universitario La Princesa; Madrid, Spain.

Jordi Gratacós; Rheumatology Department, Hospital Parc Taulí; Sabadell, Spain.

Jordi Guardiola; Gastroenterology Department, Hospital Universitari de Bellvitge; Barcelona, Spain.

Iván Guerra; Gastroenterology Department, Hospital Universitario de Fuenlabrada; Madrid, Spain.

Ana Gutiérrez; Gastroenterology Department, Hospital General Universitario de Alicante; Alicante, Spain.

Enrique Herrera; Dermatology Department, Hospital Universitario Virgen de la Victoria; Málaga, Spain.

Susana Holgado; Rheumatology Department, Hospital Universitari Germans Trias i Pujol; Badalona, Spain.

Iago Rodríguez-Lago; Gastroenterology Department, Hospital Universitario de Galdakao; Bilbao, Spain.

Eva Iglesias; Gastroenterology Department, Hospital Universitario Reina Sofía; Córdoba, Spain.

Beatriz Joven; Rheumatology Department, Hospital Universitario 12 de Octubre

Ana Laiz; Rheumatology Department, Hospital de la Santa Creu i Sant Pau; Barcelona, Spain.

Laura López; Rheumatology Department, Hospital Sant Rafael; Barcelona, Spain.

Antonio Lopez-Sanromán; Gastroenterology Department, Hospital Universitario Ramón y Cajal; Madrid, Spain.

José Luís López Estebaranz; Dermatology Department, Hospital Universitario Fundación Alcorcón; Madrid, Spain.

Marta Loredo Martinez; Rheumatology Department, Hospital Universitario Central de Asturias; Asturias, Spain.

Berta Magallares; Rheumatology Department, Hospital de la Santa Creu i Sant Pau; Barcelona, Spain.

José Luís Marenco de la Fuente; Rheumatology Department, Hospital Universitario Virgen de Valme; Sevilla, Spain.

Nacho Marín; Gastroenterology Department, Hospital General Universitario Gregorio Marañón; Madrid, Spain.

Carlos Marras; Rheumatology Department, Hospital Universitario Virgen de la Arrixaca; Murcia, Spain.

Andrea Pluma; Rheumatology Department, Hospital Universitari Vall d'Hebron; Barcelona, Spain.

Mariló Martín Arranz; Gastroenterology Department, Hospital Universitario La Paz; Madrid, Spain.

Julia Martínez Barrio; Rheumatology Department, Hospital Universitario Gregorio Marañón; Madrid, Spain.

Víctor Martínez Taboada; Rheumatology Department, Hospital Universitario Marqués de Valdecilla; Santander, Spain.

Lourdes Mateo; Rheumatology Department, Hospital Universitari Germans Trias i Pujol; Badalona, Spain.

Juan Luís Mendoza; Gastroenterology Department, Hospital Clínico San Carlos; Madrid, Spain.

Francisco Mesonero; Gastroenterology Department, Hospital Universitario Ramón y Cajal; Madrid, Spain.

Joan Miquel Nolla; Rheumatology Department, Hospital Universitari de Bellvitge; Barcelona, Spain.

Jordi Monfort; Rheumatology Department, Hospital Universitari Parc de Salut Mar; Barcelona, Spain.

Carlos Montilla; Rheumatology Department, Hospital Universitario Virgen de la Vega; Salamanca, Spain.

Estefania Moreno; Rheumatology Department, Hospital Comarcal Alt Penedès; Vilafranca, Spain.

David Moreno Ramírez; Dermatology Department, Hospital Universitario Virgen Macarena; Sevilla, Spain.

Santiago Muñoz; Rheumatology Department, Hospital Universitario Infanta Sofía / Hospital Universitario La Paz; Madrid, Spain.

Fernando Muñoz; Gastroenterology Department, Hospital Universitario de Salamanca; Salamanca, Spain.

Antonio Naranjo; Rheumatology Department, Hospital Universitario de Gran Canaria Dr. Negrín; Gran Canarias, Spain.

Natividad Oreiro-Villar; Rheumatology Department, Complejo Hospitalario Universitario de A Coruña; A Coruña, Spain.

Pilar Nos Mateu; Gastroenterology Department, Hospital Universitario y Politécnico La Fe; Valencia, Spain.

Jaime Notario; Dermatology Department, Hospital Universitari de Bellvitge; Barcelona, Spain.

Ana Ortiz; Rheumatology Department, Hospital Universitario La Princesa; Madrid, Spain.

Pedro Zarco; Rheumatology Department, Fundación Hospital Alcorcón; Madrid, Spain.

Jose María Pego; Rheumatology Department, Hospital do Meixoeiro; Vigo, Spain.

Javier P. Gisbert; Gastroenterology Department, Hospital Universitario de la Princesa; Madrid, Spain.

José Javier Pérez-Venegas; Rheumatology Department, Hospital Universitario de Jerez de la Frontera; Cádiz, Spain.

Jordi Monfort; Rheumatology Department, Hospital Universitari Parc de Salut Mar; Barcelona, Spain.

José Antonio Pinto-Tasende; Rheumatology Department, Complejo Hospitalario Universitario de A Coruña; A Coruña, Spain.

Lluís Puig; Dermatology Department, Hospital de la Santa Creu i Sant Pau; Barcelona, Spain.

Ramon Pujol; Dermatology Department, Hospital Universitari Parc de Salut Mar; Barcelona, Spain.

Rubén Queiró; Rheumatology Department, Hospital Universitario Central de Asturias; Oviedo, Spain.

Delia Reina; Rheumatology Department, Hospital Moisès Broggi; Sant Joan Despí, Spain.

Elena Ricart; Gastroenterology Department, Fundació Clínic per a la Recerca Biomèdica; Barcelona, Spain.

Pilar del Río-Martínez; Rheumatology Department, Centro de Especialidades Médicas Inocencio Jiménez; Zaragoza, Spain.

Raquel Rivera; Dermatology Department, Hospital Universitario 12 de Octubre; Madrid, Spain.

Anne Riveros; Rheumatology Department, Hospital Universitari Germans Trias i Pujol; Badalona, Spain.

Esther Rodríguez; Rheumatology Department, Hospital Universitario 12 de Octubre; Madrid, Spain.

Daniel Roig; Rheumatology Department, Hospital Moisès Broggi; Sant Joan Despí, Spain.

Mónica Roncero Riesco; Dermatology Department, Hospital Universitario de Salamanca; Salamanca, Spain.

Iñigo Jesús Rua-Figueroa; Rheumatology Department, Hospital Universitario de Gran Canaria Doctor Negrín; Las Palmas de Gran Canaria, Spain.

Tarek Salman; Rheumatology Department, Hospital Universitari Parc de Salut Mar; Barcelona, Spain.

Georgina Salvador; Rheumatology Department, Hospital Universitari Mútua de Terrasa; Terrassa, Spain.

Simón Sánchez; Rheumatology Department, Hospital General La Mancha Centro; Ciudad Real, Spain.

Jose Luís Sánchez Carazo; Dermatology Department, Hospital General Universitario de Valencia; Valencia, Spain.

Raimon Sanmartí; Rheumatology Department, Fundació Clínic per a la Recerca Biomèdica; Barcelona, Spain.

Mónica Sierra; Gastroenterology Department, Complejo Asistencial Universitario de León; León, Spain.

Carles Tomàs; Rheumatology Department, Hospital comarcal d'Amposta; Amposta, Spain.

Jesús Tornero; Rheumatology Department, Hospital Universitario Guadalajara; Guadalajara, Spain.

Juan Carlos Torre-Alonso; Dermatology Department, Faculty of Medicine, Universidad de Oviedo; Oviedo, Spain.

Paloma Vela; Rheumatology Department, Hospital General Universitario de Alicante; Alicante, Spain.

Maribel Vera; Gastroenterology Department, Hospital Universitario Puerta de Hierro; Madrid, Spain.
